# Influence of an extreme event—the COVID-19 pandemic—On establishment of and data collection by a citizen science project

**DOI:** 10.1371/journal.pone.0303429

**Published:** 2024-05-31

**Authors:** Elizabeth Y. Zhang, Annika Baldwin, Calista Hundley, Eugena Chang, Susannah Auderset, Mia Bawendi, Thea V. Kristensen

**Affiliations:** Department of Biology, Amherst College, Amherst, MA, United States of America; PLOS ONE, UNITED KINGDOM

## Abstract

The recent rising incidence of extreme natural events may significantly influence the implementation of citizen science projects, including the success of outreach strategies and the quality and scope of data collection. The MassMammals Watch and subsidiary MassBears citizen science projects, initiated during the height of the pandemic, recruit volunteers to submit sightings of black bears and other mammals. In this study, we evaluated the methods we employed for engaging and retaining community volunteers during a period of intense social restrictions, and we assessed whether such conditions were associated with spatial biases in our collected data. Newspaper features were more likely to recruit volunteers who engaged with the project multiple times, but social media and internet presence were important for reaching a larger audience. Bear sighting submissions peaked in number and were more likely to be in forested areas during 2020, the height of the pandemic, compared to later years, a pattern which we suggest stems from an increased desire to participate in outdoor activities in light of social distancing measures during that year. Such shifts in patterns of data collection are likely to continue, particularly in response to increasing extreme weather events associated with climate change. Here, we both make recommendations on optimal outreach strategies for others initiating citizen science programs and illustrate the importance of assessing potential biases in data collection imposed by extreme circumstances.

## Introduction

### Citizen science projects and the COVID-19 pandemic

Citizen science projects partner researchers with the general population for collaborative data collection [1, 2]. This partnership fosters a learning opportunity for all involved groups, with volunteers gaining scientific literacy and knowledge of ecological issues and researchers gaining valuable data [3–7]. The data gathered, which often covers a greater geographical and/or temporal extent than a single team of scientists would be able to cover alone, provides important insight into species diversity, abundance, and behavior over time [8–13].

To stay engaged and continue participating, volunteers involved in citizen science programs need clear communication and expectations, reports of their work, and progress updates on the project [10, 14–17]. Maintaining a successful program also requires that researchers continually evaluate program outcomes, including measuring the recruitment and retention of volunteers, understanding volunteer interests and demographics, and considering the quality and quantity of data collected [14, 18]. Project evaluation can inform subsequent changes to the project that allow researchers to ensure their goals are met, in addition to supporting the community in which the project is based [10–12].

However, in many cases, volunteer engagement in citizen science projects and the data collected through these projects shifted during the COVID-19 pandemic [19–21]. Outdoor citizen science programs had greater success with recruitment and engagement than other citizen science programs during the pandemic [22], but even these varied with respect to participation. For instance, while some projects experienced a decline in overall participation [1, 19, 23], other biological monitoring citizen science projects saw either no overall impact or an increase in data collection from the volunteers they retained [24–26]. In both cases, observations typically came from a small subset of participants who either sustained participation or became more active during the pandemic, often in pursuit of a hobby or sense of normalcy [1, 19, 23–25]. Thus, in some instances, the pandemic became an opportunity for citizen science projects to harness the enthusiasm of some participants under quarantine toward field-based data collection; whereas in other cases, data collection declined [1, 19, 21].

In addition to altering patterns of participation and engagement, the pandemic influenced spatial trends of observations submitted. Anuran call surveys from Appalachia, USA and iNaturalist and eBird observations in Colombia were more prevalent on private land during the pandemic as public land became less accessible in these areas [20, 25]. Additionally, while the pandemic did not substantially disrupt the collection of bird data by citizen scientists in Italy, Spain, and the UK, a surge in urban observations over rural areas occurred [24]. A point of concern is that increased observations of species in urban spaces could either be due to an increase of urban participants out searching, a temporary shift in ecological niches due to the decrease in human activity associated with the pandemic, or simply an increase in the population of the target species itself [27, 28]. Overall, there have been a variety of spatial biases reported in the literature, each of which is likely related to the format of the project and location-specific restrictions [27, 28].

It has been suggested that changes in the climate influenced the emergence and scale of the COVID-19 pandemic [29, 30]. Such trends in climate change have also been linked to increased incidence of natural disasters, more broadly, including extreme weather events [31, 32]. As the effects of climate change escalate [33], this may impact the number and location of ecological citizen science observations. Identifying and correcting for these spatial biases before modeling and interpreting citizen science data is critical for avoiding erroneous inferences [34, 35]. Given the impact that such biases might have on data analysis, we use the pandemic as a case-study to evaluate and report changes in data collection due to a extreme circumstances.

### Initiating our citizen science project during the pandemic

In this case study, we demonstrate how establishment of a citizen science project–MassMammals Watch and its subsidiary MassBears–occurred as the COVID-19 pandemic peaked and then gradually subsided. We also address methods for effective volunteer collection and spatial patterns of data observations in response to the pandemic. Similarly to other citizen science projects, we used strategies for adapting our outreach strategy based on observed patterns of recruitment. Uniquely, however, project leads were undergraduates in collaboration with an instructor at Amherst College. Volunteers provided cost-effective sources of data—in-person and trail camera sightings of mammals—allowing the project team to answer novel questions about the changing mammal populations in Massachusetts. We initiated components of our project in Fall 2019 and Spring 2020, resulting in initial stages of project establishment overlapping the outset of the pandemic. The state-wide stay-at-home advisory in Massachusetts in 2020 [36] may have impacted recruitment and activity of volunteers as well as spatial distribution of observations, particularly given that many USA residents experienced an expansion of free time [37] and a subsequent increase in participation in outdoor activities [38].

A number of studies have evaluated the process of establishing and maintaining citizen science projects, while a separate group of studies has assessed the patterns of data collection during the pandemic, which differ across the specific circumstances of each project. However, to our knowledge, there has yet to be a study on both the establishment of and data collection by a citizen science project during an extreme event, though we anticipated both aspects of our project to be impacted. Therefore, we present an investigation into both (1) the specific strategies for developing a citizen science project (MassBears/MassMammals Watch) during the progression of the COVID-19 pandemic, including volunteer recruitment and retention, and (2) the data we collected through such strategies, including potential spatial biases observed during the pandemic. Consideration of both factors allowed us to evaluate how establishment of a citizen science program was impacted by a significant world event, both in terms of volunteer recruitment and spatial distribution of data. Given the likely increase in outdoor activities during pandemic restrictions, we hypothesized that there would be a larger number of submissions, focused on areas farther from neighborhoods during pandemic activity restriction. The results of this study can inform the design and evaluation of other citizen science projects amidst significant disruptions, including extreme weather and natural disaster events.

## Materials and methods

We established our citizen science program beginning in Fall 2019. The project is organized with MassMammals Watch as the umbrella program and MassBears as a subproject. Project leaders included an instructor in collaboration with undergraduate students (hereafter undergraduate project coordinators) at Amherst College, Amherst, MA, who are given latitude to drive the direction and execution of the project. In its initial recruitment phases in Fall 2019, the project primarily focused on reaching volunteers in the surrounding Pioneer Valley area of Massachusetts (S1 Fig). As the project gained footing by the summer of 2020, recruitment efforts expanded to include all of Massachusetts, to reach a larger geographical area. We have since built a volunteer base by experimenting with a variety of outreach methods.

Specifically, over Winter 2019 and Spring 2020, we began to advertise the project to potential participants in the Pioneer Valley and then the larger Massachusetts area (S2 Fig). To make implementation more manageable, we began by focusing on MassBears. We established the MassBears website in winter 2019, and included information about the project, links to other educational sites about black bears, and a bear sighting submission form open to the public. This project operated adjacently to empirical research that the project lead was conducting on black bear population abundance in Massachusetts. In spring 2020, we established the full MassMammals Watch website, with a second sighting submission form and resources on how to identify not only black bears, but additional mammals native to Massachusetts that were of interest. In fall 2020, we began educational outreach with local schools, each of which contributed trail camera sightings to the project [39]. We retain the MassBears title as a separate subproject which falls under the MassMammals Watch. Though the projects are related, we refer to them separately throughout this evaluation due to the different contexts in which they were initiated and the separate platforms that we maintain for them.

On the MassBears site, we incorporated a map of the submitted bear sightings that is updated weekly, allowing volunteers to see how their contributions fit into the larger data set. To reach an initial volunteer base in the Pioneer Valley area, before expanding to greater Massachusetts, we reached out to local media who published four articles about the project in local newspapers through July 2020. In October 2021, undergraduate project coordinators also posted fliers at trailheads and public libraries around the Pioneer Valley. We further created Facebook and Instagram accounts in July 2020 and March 2021, respectively, to provide information on mammal ecology, recognize volunteers who submitted frequent or exemplary sightings, and keep volunteers updated on project progress. With similar goals in mind, we began emailing monthly newsletters to all volunteers and posting to our website in November 2021. Newsletters highlight a volunteer “photo of the month”, provide brief educational materials, and describe the work of the undergraduate project coordinators. Over spring 2022, we examined the map of bear sightings and recognized areas where we knew bears existed but had conspicuously few sightings. We wanted to ensure that we had a volunteer base in those areas, which would help us confirm that gaps in coverage were due to true patterns in population distribution, rather than absence of volunteers. We then targeted outreach efforts to those areas by sending fliers to local schools, libraries, and conservation areas and contacting educators. In May 2022 and Fall 2022, newspapers from around the state reached out to us and subsequently published articles that reached a broader audience.

This study was exempt from IRB review, as per the Amherst College IRB committee, due to use of anonymized and aggregated nature of the data, which was provided optionally and voluntarily by participants. Additionally, disclosing responses would not reasonably place respondents at any risk.

### Data collection and source of recruitment

MassMammals Watch and MassBears websites each include a sightings link which directs users to a Google Form where community members can submit a sighting of a mammal or bear, with the respective sighting date and location, as well as photos if available. Community members are also asked how they learned about the project (open response). Undergraduate project coordinators reviewed photo submissions to verify species identification and added bear sightings to a map of Massachusetts, which is color coded by year to visualize species trends over time. The MassMammals Watch and MassBears websites also direct interested volunteers to a separate Google Form where they can express interest in greater involvement by becoming a registered volunteer; this form includes the option to report open-response answers of how they learned about the project, what interests them about the project, their age, experience with animal identification, and how much time they spend hiking. Volunteers were also asked to select options of how they would like to be involved: collecting data on hikes, submitting trail camera photos/videos, and/or submitting mammal sightings. Registered volunteers are differentiated from sightings volunteers based on whether or not they filled out this Google Form. Their sightings are included in our analysis of total sightings (S1 Data).

### Volunteer recruitment and retention

In the initial stages of the project, we were interested in evaluating volunteer recruitment and participation. Therefore, we tracked the numbers of website visits, contributions of responses, and participants over time. These factors provide evidence of our project’s ability to connect with the community and engage volunteers to contribute data.

#### Source of recruitment

To track the impact of recruitment and outreach efforts, we created a list of volunteers who have engaged with each website, based on unique email addresses, which were collected as an optional question on all Google Forms (both sightings and registered volunteers). We then anonymized submissions and examined open responses to the question asking where volunteers had heard about the project. We categorized recruitment sources into 5 major categories (friend/community, internet search/advertising from Amherst College, newspaper, project member/contributor, and social media) and determined the proportion of people selecting each category. For the MassMammals Watch website, we assessed overall recruitment sources from all levels of participation. For the MassBears website, we categorized volunteers by three levels of participation by counting the number of submissions that they had submitted from the inception of the project until August 15, 2022. We then looked at recruitment sources across those three levels of participation to assess whether certain outreach strategies were more effective for reaching participants with more sustained levels of engagement. We also looked at the counts of volunteers within each level of participation to help assess how effectively our project retained long-term volunteers.

#### Volunteer engagement over time

Patterns in sighting submissions, levels of visitorship to our websites, and patterns in recruitment of registered volunteers each contributed to our evaluation of volunteer engagement. We tracked patterns of submissions to each of our MassBears and MassMammals Watch websites by visualizing the number of sightings reported per month from the inception of the project. We ran a two-way ANOVA to assess whether there were differences in the number of weekly sightings by year and season. We categorized the start of the week as a Sunday, and we assigned season based on that start date. Seasons were assigned based on months (winter = 12, 1, 2; spring = 3, 4, 5; summer = 6, 7, 8; autumn = 9, 10, 11). Finally, years were assigned based on season, so December of one year would be categorized with the following year. We employed post-hoc Tukey’s HSD tests to assess differences across year and season.

Additionally, we tracked traffic to the MassBears and MassMammals Watch websites over time by employing Google Analytics to count the number of visitors per month from June 2019 and June 2020 to the MassBears and MassMammals Watch websites respectively. We noted peaks in visitorship and sightings that appeared within 1–2 months of an outreach initiative, such as publication of a newspaper article or posting of fliers. The cumulative number of registered volunteers who signed up through the form on the MassMammals Watch website by month allowed us to track patterns in volunteer recruitment. Registered volunteers regularly participate in the project by submitting data from trail cameras, so we further assessed patterns in their recruitment to measure our project’s ability to reach more committed volunteers.

#### Registered volunteer demographics and interest

Finally, we assessed volunteer demographics and interests. Registered volunteers responded to optional questions asking their age and the frequency with which they hike, walk, or participate in other outdoor activities. We report volunteers’ average age, the proportion of volunteers over 50, and the proportion of volunteers who indicate that they participate in outdoor activities several times a week. To measure the range of interests expressed by volunteers, we analyzed open responses on the registered volunteer form for MassMammals Watch to the question: What interests you about the project(s)? We grouped responses into the five following categories: experience observing wildlife; passion for nature, animals, and wildlife protection; protecting/coexisting with wildlife; learning about nearby wildlife; and contributing to conservation research. Some responses fit into multiple categories as volunteers mentioned more than one interest. As we did not want to reduce the information from these responses, we counted instances where a single respondent selected multiple categories in one response as multiple sources of interest.

### Changes in sightings due to the pandemic

We examined the pattern of location of sightings each year in two ways, to assess whether difference may have occurred in response to conditions associated with the pandemic. First, we used self-reported categories of locations, which included five options: yard or neighborhood, forest, farm or field, road, or other (fill-in description of location). If other was chosen, we manually categorized filled-in locations in one of the listed categories or left it as the generalized “other.” We chose only to retain data from 2020–2022 as we received only 23 sightings in 2019; we also removed sightings submitted outside of active bear season (which we defined yearly as April 15th to October 15th), as we observed fewer sightings with greater variation in location during this time period. We used a chi-squared test for homogeneity to assess whether the distribution of sightings self-reported from a yard or neighborhood, as compared to all other self-reported locations, remained constant across the years 2020–2022.

Second, we examined spatial locations of sightings using self-reported addresses or GPS coordinates. When participants submitted GPS coordinates for the sighting, we preferentially used those over addresses. Otherwise, we located the coordinates from the street address using GeoCode, which transforms street addresses into GPS coordinates using Google Maps’ search engine. We used a forest cover data layer provided by a collaborator (as per [40]). We visualized trends by grouping sightings into month-long periods, with each period starting on the 15^th^ of a given month. It should be noted that this and the chi-squared tests are the only analyses in which month-long periods begin on the 15^th^; all other visualizations define months as beginning on the 1^st^. We also ran a one-factor ANOVA analysis on forest cover at sightings between 2020, 2021, and 2022. We used a post-hoc Tukey multiple comparisons to determine which years differed with respect to forest cover. Prior to running analyses, we checked that the conditions for both the chi-squared test of homogeneity and the one-factor ANOVA analysis were met.

## Results

### Volunteer recruitment and retention

#### Source of recruitment

A total of 1052 sightings have been reported through MassBears since the project inception and 178 via MassMammals Watch. The MassBears site had 453 responses that indicated how they found out about the project. Online searches about bears brought us 32.5% of participants, followed by social media and newspapers at 24.9% and 22.1% respectively for MassBears (S3 Fig). On MassMammals Watch, people may also become registered volunteers, who intend to contribute over time. Out of the 87 registered volunteers, 85 indicated where they learned about the project. Around 40% read about MassMammals Watch in a newspaper article, the most common being the Hampshire Gazette. These were followed by social media, which was dominated by Facebook. We only recently began accepting individual sightings on MassMammals Watch, but for those respondents, the most common source was newspapers at 40%, followed by friend/community and project member/contributor, both at 29%. In addition, there may be crossover between the participants who submitted responses with a sighting and those who submitted one as an official volunteer.

We had a total of 496 individuals with unique emails report bear or mammal sightings through our MassBears and MassMammals Watch projects. Across all levels of engagement, 38%, 22%, 21%, 18%, and 1% of volunteers heard about the project through internet searches/Amherst College, newspaper, social media, a friend/community member, and a project member/contributor, respectively. Web presence, through websites and social media, thus accounted for the recruitment of over half (59%) of our project’s participants.

**Fig 1:**
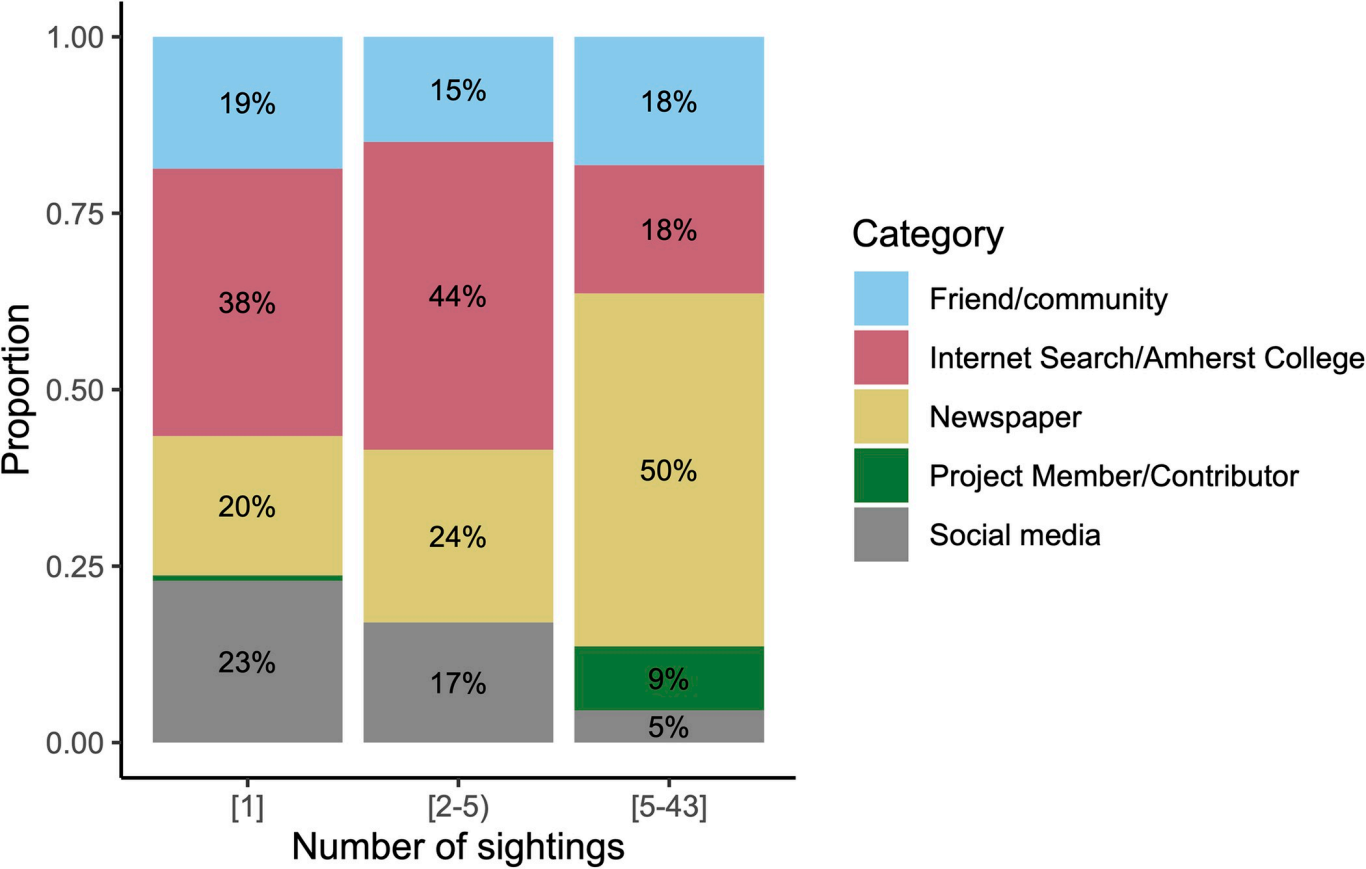
Recruitment methods for volunteers who submitted a number of sightings within the specified ranges. Individuals were identified by unique emails from an optional question on the MassBears sightings form. Recruitment methods were categorized from an optional question on the MassBears sightings form. (n = 380, n = 94, n = 22).

We also visually assessed the relationship between the source of recruitment and engagement level, which we measured through the number of sightings reported. 76.6% sightings volunteers contributed only one sighting (n = 380), 19.0% contributed 2–4 sightings (n = 94), and 4.4% contributed 5 or more sightings (n = 22). For those contributing multiple sightings, the newspaper becomes the most frequent source of recruitment, accounting for 20% of those who contributed only once up to 50% of those who contributed 5 or more times (Fig 1). Hearing of the organization from a project member/contributor accounts for a notable proportion of submissions only for those who have submitted five or more sightings. Internet searches and social media also tend to account for fewer recruitments as the number of sightings increases. The proportion who heard from friends or their community is relatively stable. Thus, it appears that more impactful sources of recruitment include newspaper articles and project members/contributors.

Unregistered volunteers submitted a median of 1 sighting with an interquartile range from 1 to 1 sighting and a maximum of 21 sightings (n = 353), while registered volunteers submitted a median of 2 sightings with an interquartile range from 1 to 5 sightings and a maximum of 43 sightings (n = 27) during the duration of the project. The numbers of sightings for both unregistered and registered volunteers are right skewed, but the distribution for unregistered volunteers has a much longer right tail.

#### Volunteer engagement over time

**Fig 2:**
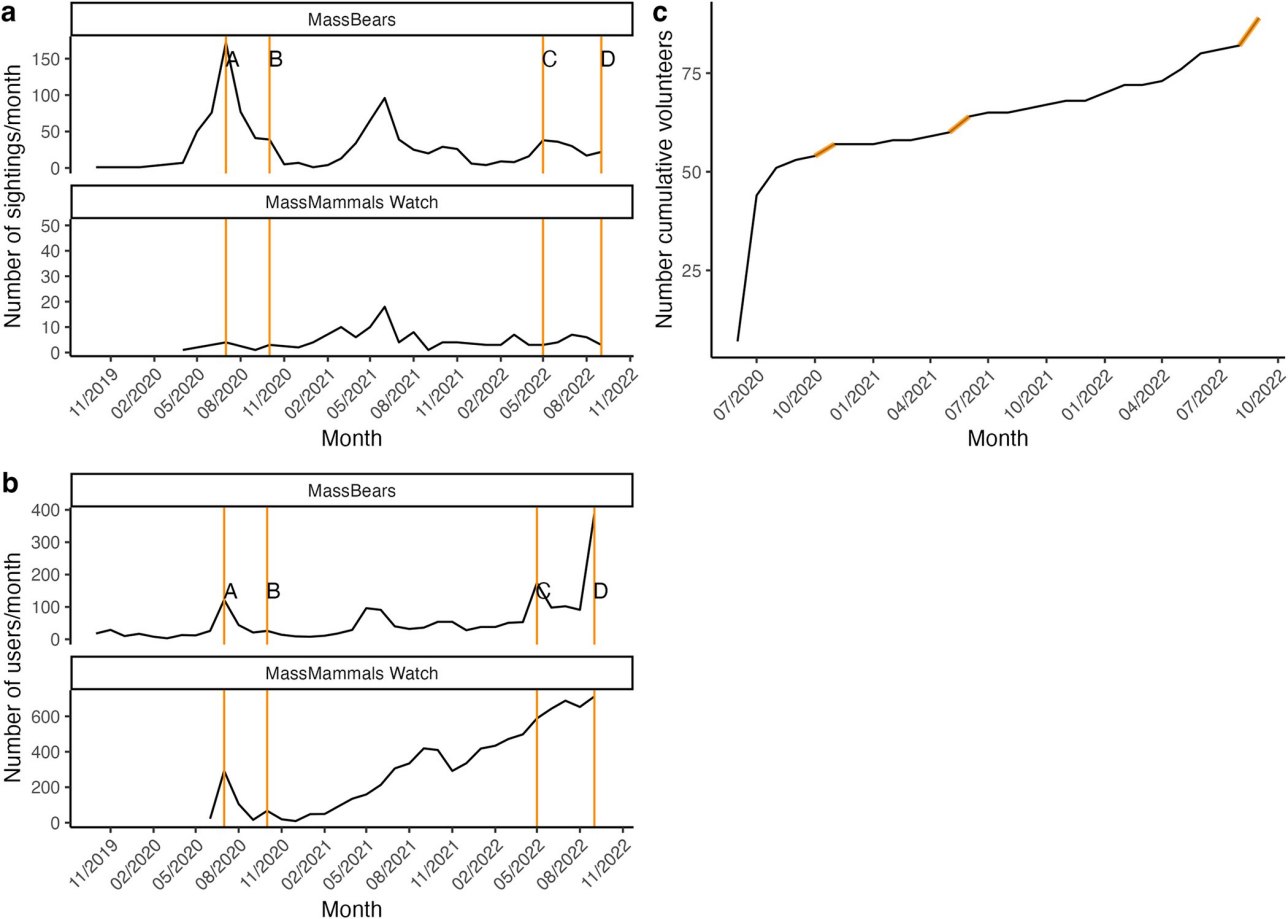
Number of sightings submitted per month (a) and number of website visitors per month (b) for the (1) MassBears and (2) MassMammals Watch projects, and cumulative number of registered volunteers for MassMammals Watch over time (c). Months are broken up by the first of each calendar month. Data was collected from the inception of each project until October 1st, 2022. Website visitorship was tracked using Google Analytics (MassMammals Watch n = 8809, MassBears n = 2139). Registered volunteers sign up through a form on the MassMammals Watch website (n final = 83). Vertical lines represent features in newspaper articles: (A) Daily Hampshire Gazette– 07/09/2020, MassLive– 07/14/2020, Berkshire Eagle– 07/25/2020; (B) Amherst College e-News 10/06/2020; (C) Boston Herald– 05/07/2022; (D) Boston Globe– 09/08/2022.

Throughout the entire study period, there were 1052 reported sightings through the MassBears website, and 178 reported sightings through the MassMammals Watch website (Fig 2A). The number of sightings submitted through the MassBears website varied across years (F = 21.79 P<0.001), with the highest number in 2020 compared to other years (S1 Table). The number of sightings also varied by season (F = 20.36, P<0.001), with the largest number in summer followed by spring, august, and winter (S1 Table). In both 2020 and 2021, the greatest number of MassBears sightings were reported in June or July, with observations rising with the start of March (Fig 2A). This suggests a seasonal pattern with respect to the number of sightings. Furthermore, the highest level of sightings occurred during 2020, in the peak of pandemic restrictions and quarantine. Since 2020, the number of peak sightings per year has successively declined. Similarly, for MassMammals Watch sightings, annual peaks occurred during the summer. However, here the years are reversed, with 2021 having more sightings than 2020. There do not appear to be sharp peaks in sightings during any of the months in which newspaper articles were published, except for submissions to the MassBears website during July 2020, which may also be attributed to the peak of the pandemic. We did observe subtle peaks in submissions to the MassBears website in the months of November 2020, May 2022, and November 2022 when newspaper articles were published, which could possibly be attributed to newspaper article publications (Fig 2A).

The number of visitors to the MassBears site had annual peaks during each summer, with a general trend of increasing visitorship from project conception in 2019 to 2022 and a maximum of 174 visitors in May, 2022 (Fig 2B). Though MassMammals Watch also experienced a slight increase of visitors in the summer, the peak is less noticeable than for the MassBears website. Moreover, the MassMammals Watch appears to have experienced a larger, steady increase of visitorship over time, with a maximum of 688 visitors per month in July, 2022, far surpassing maximum visitorship to MassBears (Fig 2B). It appears that MassMammals Watch did not experience the same dip in visitorship during the winter months as MassBears did. There also appeared to be moderate peaks in visitorship to the MassBears website in the months where the MassBears project was included in a newspaper (July 2020 and May 2022). A significant peak in visitorship to the MassBears website occurred in September 2022, when our project was featured in a Boston.com article. Though the MassMammals Watch website experienced a peak in July 2020, it did not experience peaks in the months of the other months in which articles were published.

Most of our registered volunteers (37, 42.5%) signed up in July of 2020 (Fig 2C). This may correspond to the timing of article publications about our project, as the Hampshire Gazette and MassLive published articles in July 2020 (Fig 2C). The number of registered volunteers has increased at a much slower rate since that time (within the range of 0–6 new volunteers per month).

The decrease in new registered volunteers does not correspond to a similar long-term decrease in observations, suggesting that either many of our participants are not consistent/registered volunteers, or that the same few registered volunteers submit the majority of sightings. In addition, those who have not accessed our website may not have known about becoming a registered volunteer.

#### Registered volunteer demographics and interests

As of October 15, 2022, 83 volunteers have filled out the Registered Volunteer Interest Form. Our registered volunteers’ ages range from 18 to 79. The typical age of a registered volunteer is 49.9 ± 16.22 SD, and 59.0% of our volunteers are 50 or older. 68.7% of our volunteers report that they hike, walk, or participate in other outdoor activities several times a week.

**Fig 3:**
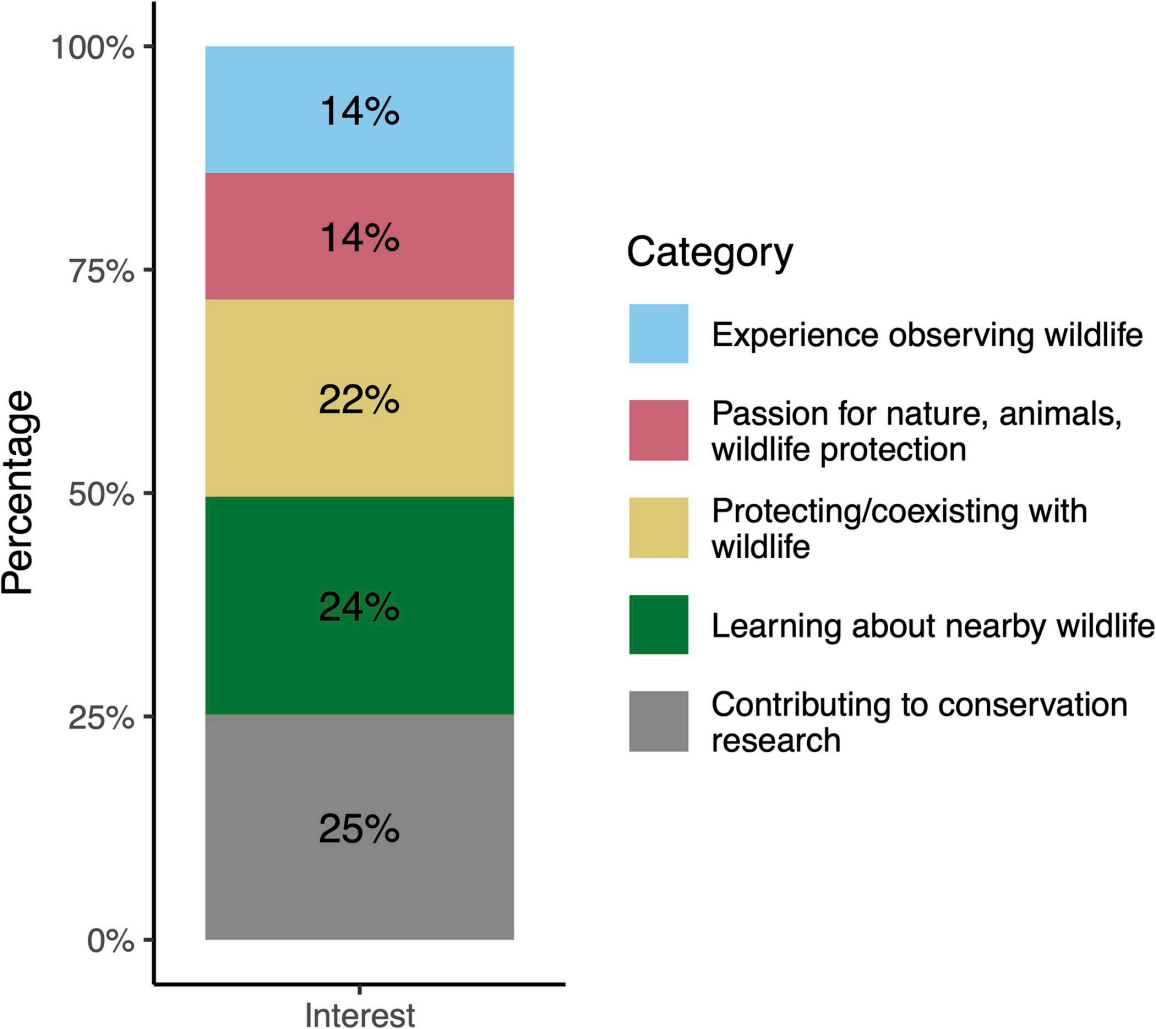
Categories for reported reasons of interest in the project from registered volunteer interest form. Responses which correlated to multiple categories were broken down into those categories, each of which then counted as one source of interest (n responses = 89, n interests = 117).

In response to the question about what interested them about the project, there was a similar frequency of the two top motivations for volunteering: contributing to conservation research and learning about wildlife (Fig 3). Volunteers reported interests in the experience observing wildlife category at the lowest frequency. Overall, there was a relatively even distribution of all categories of interest in the project.

### Changes in sightings due to the pandemic

#### Self reported locations

**Fig 4:**
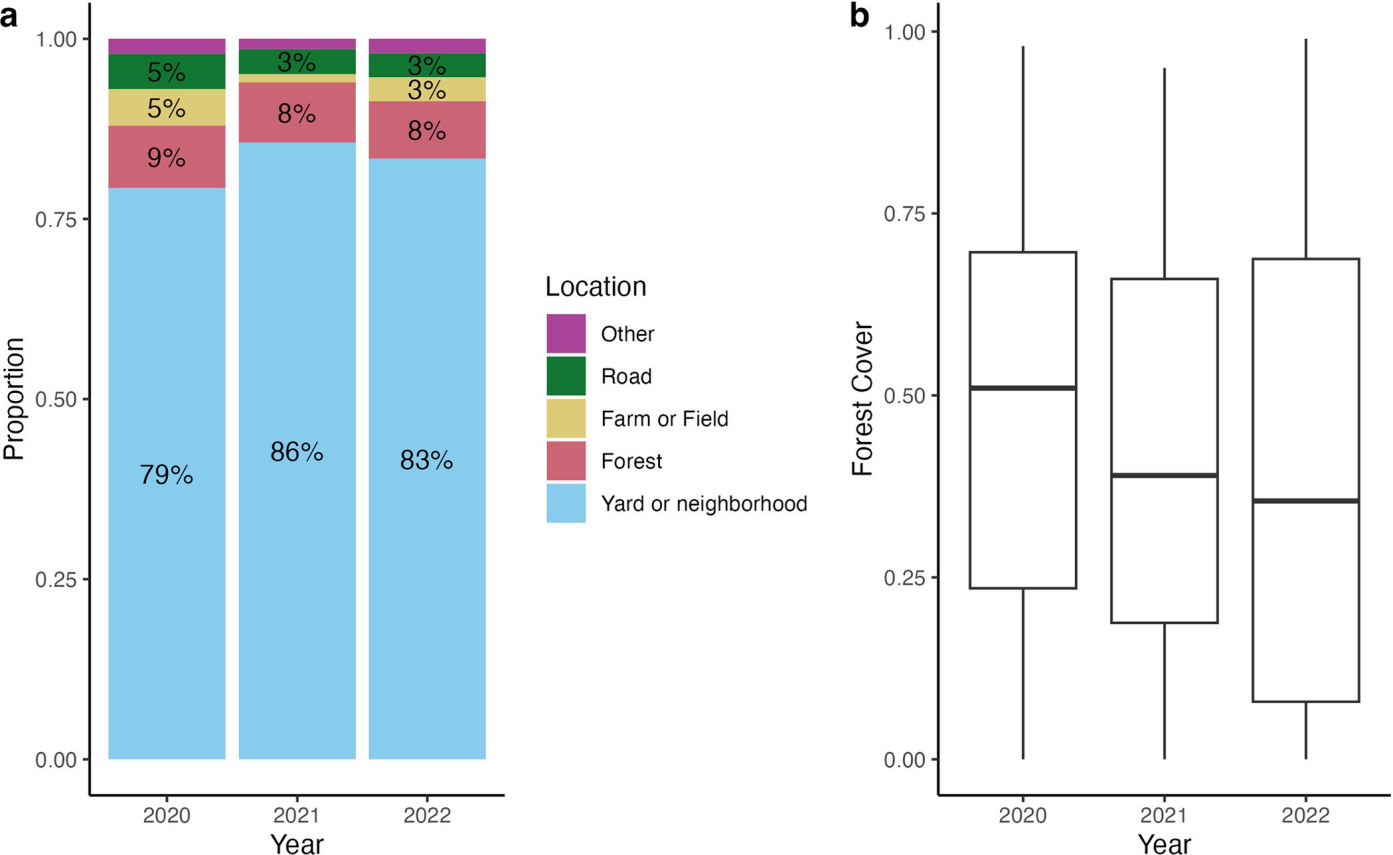
The percent frequency of self-reported bear sighting locations (a) and distribution of forest cover extracted from sighting locations (b) in 2020, 2021, and 2022 through October 15th. Volunteer responses in (a) were collected from an optional question on the MassBears sighting submission form (2020 n = 473, 2021 n = 347, 2022 n = 150). Percentages less than or equal to 3% are not labeled due to size of bars. Only data from the active bear season is retained in (b), which we define yearly as April 15th to October 15th (n 2020 = 442, n 2021 = 271, n 2022 = 143).

From 2019 to 2022 an average of 82.6% of sightings were self-reported to have come from yards or neighborhoods, 8.2% in forests, 4.1% in roads, 1.8% in blueberry fields, and 3.3% in other self-reported locations (Fig 4A). Since 2020, the proportion of sightings from farm, road, field, and other locations has fluctuated, while the proportion of forest sightings has remained fairly stable. In 2019, there were only 23 sightings from 2 types of locations reported: 95.7% from yards or neighborhoods and 4.3% on roads. Since 2020, yard and neighborhood sightings have fluctuated, from 79.1% in 2020, to 85.9% in 2021, and 83% in 2022 (which only included data up until October 15). Though there appears to be variation between years in the proportion of sightings reported in a yard or neighborhood, with more sightings submitted in yards or neighborhood after 2020, this difference is only marginally significant (χ^2^ = 5.6, p = 0.06, Fig 4A).

#### Locations extracted from geographic coordinates

Average forest cover for sightings tended to decrease throughout each active bear season (S4 Fig), and also on average from 2020 to 2022 as the pandemic progressed (Fig 4B). Based on an overall significance level of 0.05, the forest cover of sightings in 2020 was significantly higher than the forest cover of sightings in 2021 and in 2022. The forest cover of sightings did not differ between 2021 and 2022 (S2 Table).

## Discussion

In the process of establishing a citizen science program (MassBears/MassMammals Watch) during the COVID-19 pandemic, we identified multiple strategies for volunteer recruitment (1), and detected differences in sighting submissions due to pandemic restrictions (2). We found that newspaper features were associated with recruitment of more dedicated volunteers, but social media and internet presence remained important for reaching a wide audience. With respect to differences in observations during the pandemic, bear sighting submissions spiked in number and were more likely to be in forested areas during 2020, the height of the pandemic, as compared to later years. Thus, we observed that our citizen science program engaged new volunteers, even during a pandemic, but that the locations in which they participated changed when lockdown measures were lifted.

### Establishing a citizen science program during a pandemic

We established a citizen science program during the pandemic, with over 1000 sightings reported to date, a group of registered volunteers, and an educational outreach component reaching over 12 local schools [39]. Evaluating citizen science programs is challenging, but vital to continued success of projects [11, 12, 14]. By monitoring traffic to our websites and observing trends in sightings, we were able to identify strategies that worked with respect to volunteer recruitment as well as identify areas for improvement. Periodically monitoring citizen science programs allows researchers to adapt and adjust the program to address volunteer response, align with interests of participants, and meet research needs of the project [11, 12].

Obtaining coverage in newspaper articles facilitated traffic to our websites, increased numbers of volunteers, recruited our most active volunteers, and resulted in greater retention of volunteers over the long term. Noticeable spikes in visitation to our website occurred in the months where a newspaper article was published. Newspaper articles often contained detailed information about our project, quotes from project contributors, photo submissions from previous volunteers, and a link to our website. Though mass communication has increasingly shifted to social media in recent years, newspapers are a critical form of outreach for reaching audiences who do not engage with social media and presenting information in a more trustworthy manner [41]. We suggest that the credibility of newspapers may have encouraged readers to become more active, long-term volunteers than social media or internet searches might. Further, our most active volunteers tended to be over 50, which corresponds to the age group which has been reported to be the most frequent consumers of newspaper articles [42].

We also maintained an active internet presence through our websites and social media accounts. Both platforms allowed us to reach new volunteers and provide educational resources, with websites providing the means of data submission. Though social media and internet presence were less likely to recruit volunteers with sustained interest in our project, they accounted for the majority of participants across all levels of engagement. Moreover, monthly patterns in website visitation did not consistently correspond to patterns in submission, suggesting that visiting the website did not immediately lead individuals to participate in our project. Still, internet searches accounted for almost 40% of volunteers across all levels of engagement, so we emphasize the importance of maintaining an easily searchable website for reaching wide audiences. We affirm previous citizen science case studies that point to social media as a valuable source of outreach for reaching a wider audience [43], but recognize that alternative strategies are also required for promoting sustained engagement [6]. Ultimately, while internet presence and social media may help reach the largest number of volunteers, newspaper presence was most important for reaching long-term volunteers. A varied range of outreach strategies remains valuable for reaching a diverse group of participants.

A challenge we faced with our citizen science project was obtaining multiple sightings from more casual contributors and continuing to recruit registered volunteers. Around three quarters of MassBears sighting submission volunteers submitted only one sighting. Additionally, we recruited 37 registered volunteers within the first month of initiating our MassMammals Watch project but thereafter only recruited 0–6 additional volunteers each subsequent month. Personal communication encourages retention and commitment in citizen science projects [16], which was more challenging during the pandemic. Other projects have found that emailed newsletters were the preferred form of communication for long term volunteers [11]. These newsletters increased observations from participants [44], helped maintain participation during the pandemic [45], cultivated a community among volunteers [16], and contributed to knowledge acquisition [18]. As a result, we implemented emailed newsletters starting in 2021, and have anecdotally received positive feedback over email, Twitter, and in-person. To work to improve communication with volunteers and encourage continued participation, we are incorporating educational elements, as well as personal stories about undergraduate and community participants in the project. Implementing such positive reinforcement in communications can help to address volunteer dropout [46, 47].

A desire to protect and learn about wildlife and to contribute to conservation were the strongest motivators among our registered volunteers. This aligns with other projects in which the motivations often included helping the environment/community, contributing to conservation, learning more about the topic, and contributing to scientific knowledge [48–53]. Volunteers want the outcome of their labor to directly affect the issue they are monitoring, suggesting the importance of communicating results to the volunteers to show that they are actively involved in the research process [49, 52]. Motivations may change over time for project participants and may be responsive to acknowledgement of contributions [53]. However, over the long term, helping the environment and gaining knowledge continue to be important for long-term volunteers in environmentally oriented projects [54]. Due to the desire to be kept apprised of the work on the project, we plan to incorporate these types of updates in further newsletter communications with volunteers and will reassess motivations periodically so that we can appropriately support volunteers.

Based on our experience establishing a citizen science program—and doing so during a challenging time frame—we recommend working to obtain newspaper coverage of the program, complementing that coverage with an online presence through social media and websites, and maintaining personalized communication with volunteers. Because motivations strongly impact volunteer experience, retention, and contribution [55, 56], we will develop a more robust structure for garnering feedback from our participants and learning about their motivations for contributing to the project. Past studies have found that in-person interactions with volunteers support volunteer retention and motivation [16], and we plan to implement opportunities for face-to-face meetings with participants now that COVID restrictions have ended. Finally, recognizing that our most active volunteers are over 50 similarly to other outdoor citizen science programs [57], we will endeavor to increase the diversity of participants by exploring methods of targeted advertising.

### Changes in sightings due to the pandemic

The initiation of our project during the height of the COVID-19 pandemic resulted in unique trends in data submissions as the pandemic progressed. We observed a peak in black bear sightings submissions during the summer of 2020, which later declined during 2021 and 2022. Some citizen science projects also maintained or increased contributions during the pandemic (i.e. observations collected through iNaturalist in Italy, Spain, and the United Kingdom [24], through both iNaturalist and eBird in Colombia [20], and through the Echidna Conservation Science Initiative in Australia [26]). However, others (i.e. The BioBlitz event in Tokyo, Japan [19], the Southern African Bird Atlas Project [1], and the Christmas Bird count in USA/Canada [23]) had a decline in participation. It is likely that varying restrictions during the pandemic influenced the levels of participation in these projects [1, 23, 26]. Moreover, during the height of the pandemic, residents in Massachusetts experienced fewer restrictions on outdoor movements [36] and gained greater amounts of free time [37], which may have contributed to increased submissions to our website. The subsequent reduction in sightings to our project, which we observed despite sustaining consistent outreach efforts, indicates a possible gradual decline in attention to outside projects as the pandemic progressed and subsided. Thus, we support prior findings of differences in data submissions during the pandemic [19, 24] and extend this work by observing that the initial impacts of the pandemic on volunteer participation did not persist over time.

We also observed that the average forest cover at black bear sighting locations was significantly higher in 2020 compared to 2021 and 2022. This is in contrast to prior findings that citizen science observations increased in urban spaces during the height of the pandemic [24, 27, 28]. We attribute this to a change in volunteer behavior during the pandemic in our study area. Vermont, USA residents reported increased participation in certain outdoor activities during the pandemic: walking, hiking, and watching wildlife [38]. Because Western Massachusetts is an area with similar demographics to Vermont; we suggest that our volunteers in Western Massachusetts likely spent more recreation time outdoors observing nature during the height of the pandemic, which could explain the larger proportion of forested sightings we received. As such, we confirm previous literature that detected spatial biases in data collected in Citizen Science projects during the pandemic and highlight the importance of considering that bias when interpreting results [24, 27, 28]; however, we report a novel pattern of increased sightings in more forested/rural areas in contrast to increased urban observations detected elsewhere.

Collecting data through such citizen science initiatives allows researchers to address scientific questions with a broader range and scope of data in comparison to most empirical methods [8–13]. However, a consistent concern with citizen science projects is the impact of bias due to human survey patterns on the data collected [34, 35]. Here, we identified differences in the number and spatial distribution of observations in response to social restrictions imposed around the Covid-19 pandemic. Climate change has been associated with increasing numbers of weather events [32, 58] that may continue to impact citizen science data collection. For instance, there has been an increase in wildfires [59] and corresponding number of days with low air quality [60, 61], days with extreme heat [62, 63], and increase in precipitation in some areas [64]. There is evidence that while people increase outdoor activity in warm weather, this tendency is reversed in response to air quality warnings or when the temperature exceeds the comfort zone [65–68]. These behavioral changes may influence patterns of data submitted. As such, our findings suggest that review of data collected under such circumstances can help identify spatial biases due to unique project contexts.

### Limitations

We initiated outreach for our citizen science project a couple months before the COVID-19 pandemic led to abrupt shut-down measures. Thus, we do not have substantial data from our project prior to the pandemic for comparison, though we offer insights as to how the nature of our data have changed as the pandemic progressed. We assume that the trends we see in volunteer participation in our project can be primarily attributed to the changing nature of the pandemic. However, as our initiative was in its early stages during the height of the pandemic, it is not possible to fully disentangle whether observed trends are due to the pandemic or the conditions of our project’s initial expansion. Additionally, we were further limited by the pandemic in what strategies could be used for engaging with the community. Initially, all contact was remote, with limited opportunity to interact with individuals involved in the community. In person interactions are strong motivators for engagement with citizen science projects [16], so this may have negatively impacted our ability to engage members of the community in our project. As mentioned above, we will endeavor to increase this type of outreach in the future.

## Conclusion

By establishing a citizen science program during the pandemic, we were able to identify strategies for recruiting volunteers during a challenging time, as well as characterize differences in patterns of submissions during the height of the pandemic. Newspaper article features recruited the most long-term volunteers, but social media outreach and website maintenance account for the majority of volunteers across all levels of engagement with the project. Communication with volunteers, as with our newsletters, was advantageous. Volunteers’ motivations for joining the project included not only a passion for wildlife, but an interest in contributing to the preservation of local ecosystems. The challenges we faced with maintaining volunteer contributions highlight the importance of maintaining active modes of communication with participants. Finally, we observed heightened participation, with sightings in more forested areas, during the peak of the pandemic in summer 2020—patterns which subsequently diminished as the pandemic subsided. This observation differed from those reported in the literature and was thus likely a result of the project format and location-specific restrictions. As such, we recommend that other projects review data collected during the pandemic and other extreme circumstances to identify potential spatial biases and avoid misinterpreting findings. This evaluation of the strategies, challenges, and outcomes from our project may serve as a useful guide for others planning to implement citizen science initiatives, and we advocate for the importance and timeliness of scientific collaborations between research institutions and local communities.

## Supporting information

S1 Fig Timeline of project development for MassBears and MassMammals Watch citizen science projects in Massachusetts, USA (Fall 2019–2022). (TIF)

S2 Fig Map of project location for MassBears and MassMammals Watch citizen science projects (2019-present). The project was initially based in the Pioneer Valley, which is outlined in blue, and then expanded to the entire state. Base layer from OpenStreetMap 2022 [69]; reprinted under a CC BY license with permission from OpenStreetMap Foundation (OSMF), 2022.
(TIFF)

S3 Fig The percent of volunteers who learned of the project from each source. Volunteer responses were collected from an optional question on the (a) sightings form found on the MassBears website, (b) the MassMammals Watch volunteer form, ((a) n = 171, (b) n = 83). The label on project member/contributor is removed due to the small size of the category, but it equaled 1.20%. The category of “Friend/Community” includes contacts from work and school environments.
(TIF)

S4 Fig Average forest cover of bear sightings per month during active bear seasons. Active bear season is defined yearly as April 15th to October 15th. Month averages were calculated by designating the 15th of each month as the monthly break. Standard errors are shown in gray.
(TIF)

S1 Table Posthoc comparison of weekly number of sightings by year and by season. Sightings reported to MassBears Website from 2019–2022. Overall comparisons by year and season were both significant at an alpha level of 0.05 (F = 20.36, p<0.001 and F = 21.79, p<0.001, respectively).
(DOCX)

S2 Table Posthoc comparison of forest cover at sighting locations by year. Sightings reported to MassBears Website from 2020–2022. Overall comparisons by year were significant at an alpha level of 0.05 (F = 6.047, p = 0.002).
(DOCX)

S1 Data Data used for analyses in the manuscript. “MassMammals Website Visitors” and “MassBears Website Visitors” were used to analyze monthly visitation to websites. “Volunteer recruitment and motivation: MassMammals registered volunteers” includes anonymized coded responses to the registered volunteer form for MassMammals and is used to analyze their recruitment source and motivations. “Dates of volunteer reported mammal sightings (MassMammals)” indicate dates of mammal sightings reported to the MassMammals website. “Volunteer reported location of bear sightings (MassBears)” includes anonymized sighting submissions to MassBears, including date, self-reported location type, unique email ID, and source of recruitment, and is used to analyze level of participation and recruitment source and spatial biases in self-reported location. “Date of sightings and corresponding percent forest cover” is used to analyze spatial biases in forest cover of sightings due to the pandemic.
(DOCX)
