# Supplementary figures and images for: Influence of an extreme event—the COVID-19 pandemic—On establishment of and data collection by a citizen science project
Source: PLoS One. 2024 May 31;19(5):e0303429. doi: 10.1371/journal.pone.0303429 (PMC11142546; doi:10.1371/journal.pone.0303429)

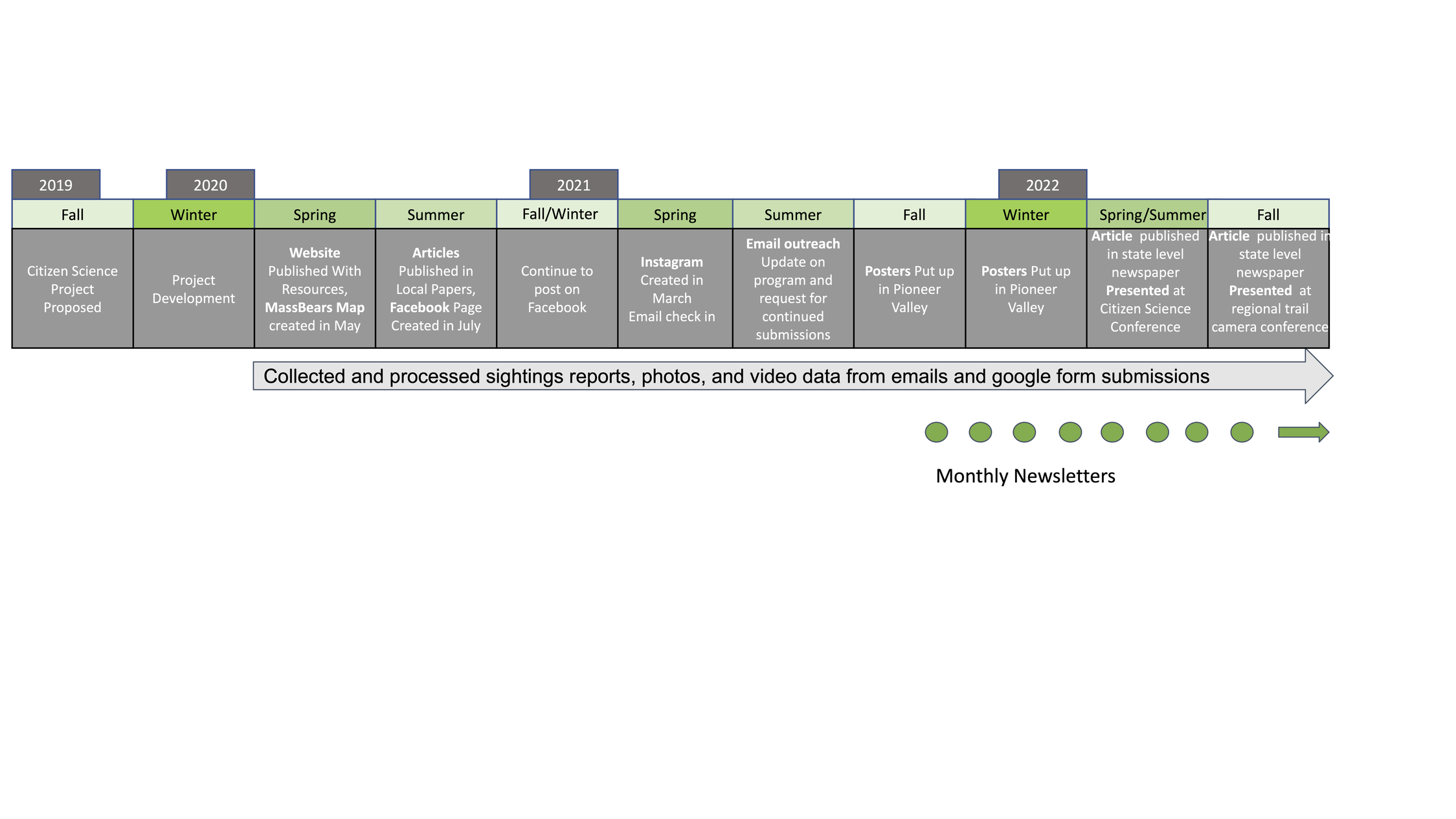

Supplement: S1 Fig — (TIF) [file pone.0303429.s001.tif]

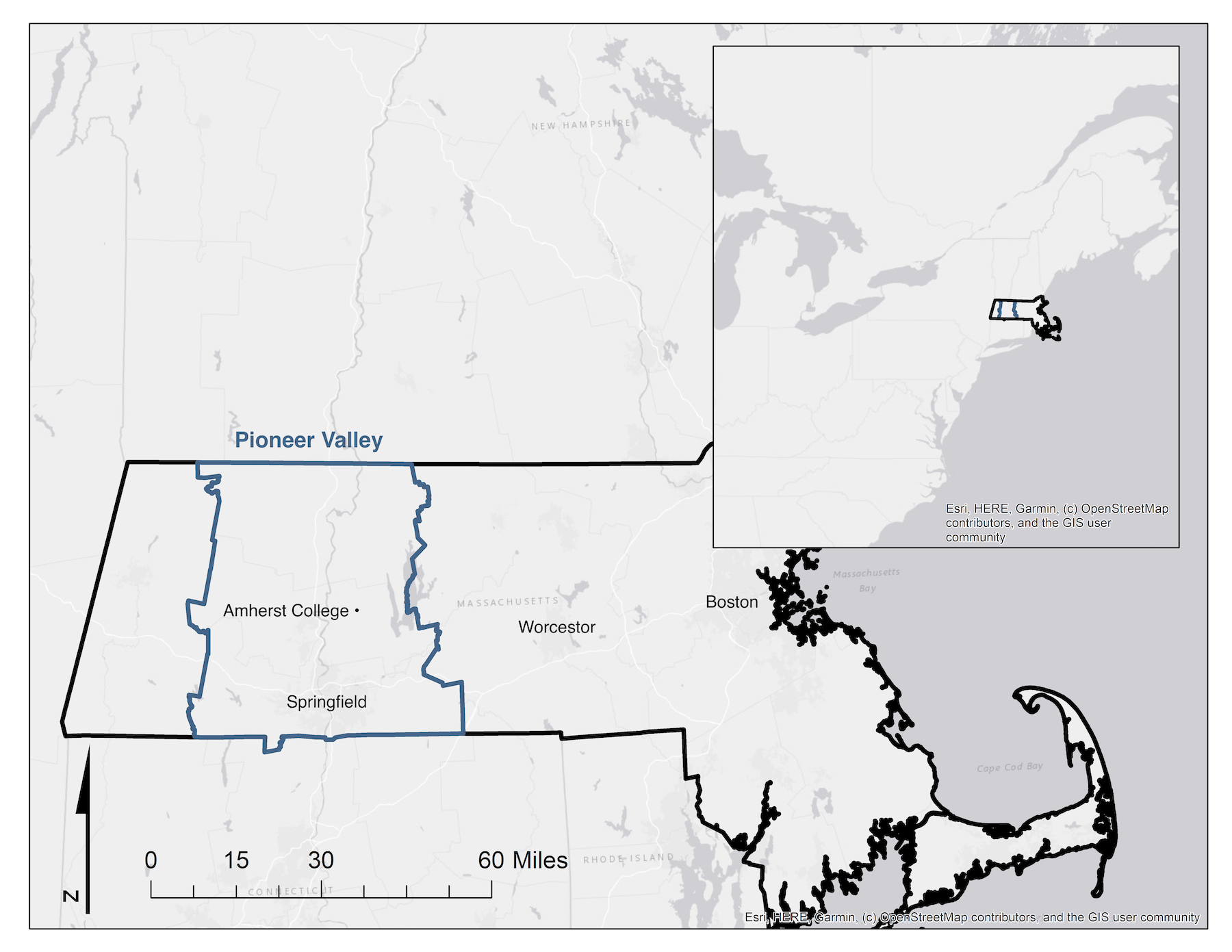

Supplement: S2 Fig — The project was initially based in the Pioneer Valley, which is outlined in blue, and then expanded to the entire state. Base layer from OpenStreetMap 2022 [69]; reprinted under a CC BY license with permission from OpenStreetMap Foundation (OSMF), 2022. (TIFF) [file pone.0303429.s002.tiff]

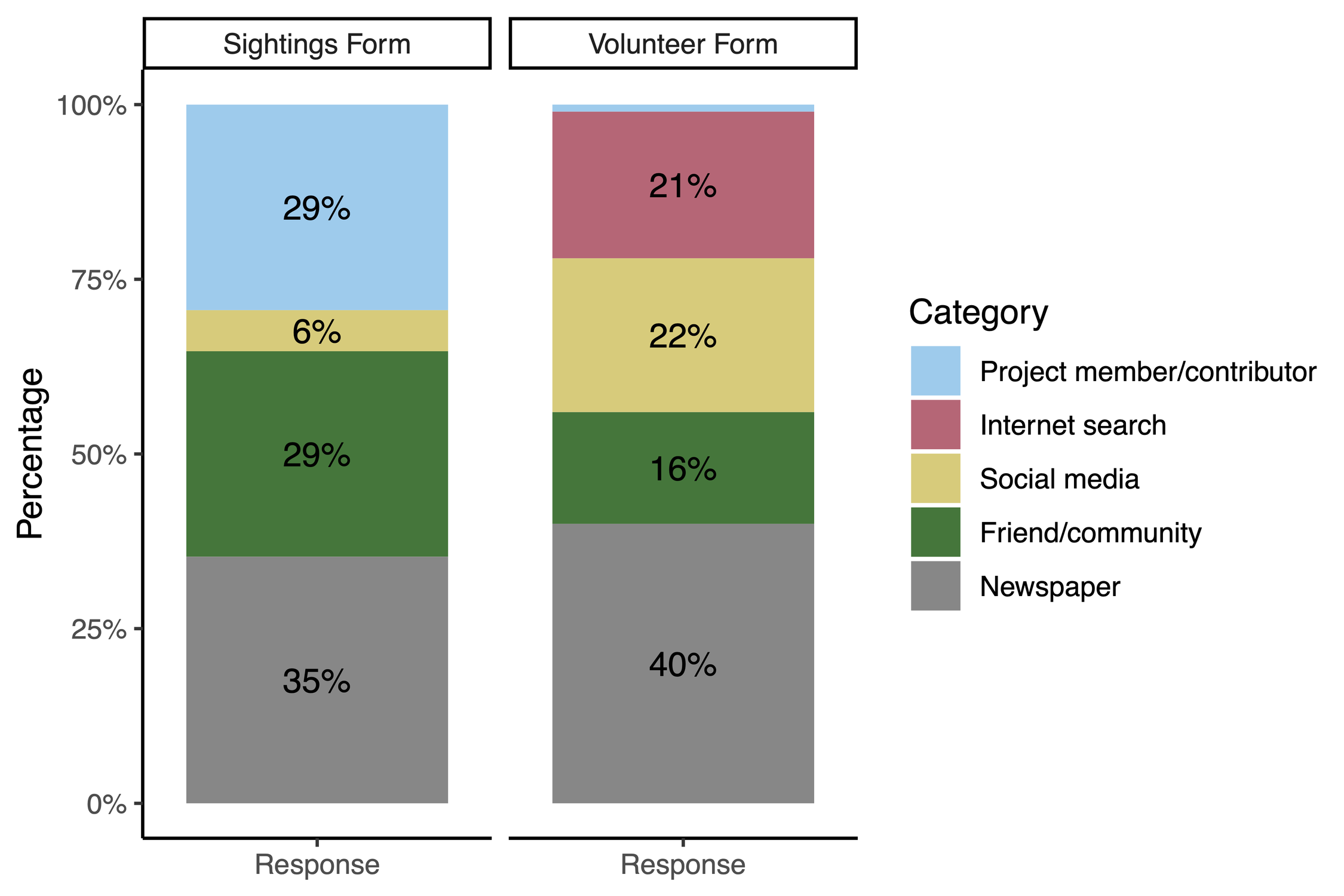

Supplement: S3 Fig — Volunteer responses were collected from an optional question on the (a) sightings form found on the MassBears website, (b) the MassMammals Watch volunteer form, ((a) n = 171, (b) n = 83). The label on project member/contributor is removed due to the small size of the category, but it equaled 1.20%. The category of “Friend/Community” includes contacts from work and school environments. (TIF) [file pone.0303429.s003.tif]

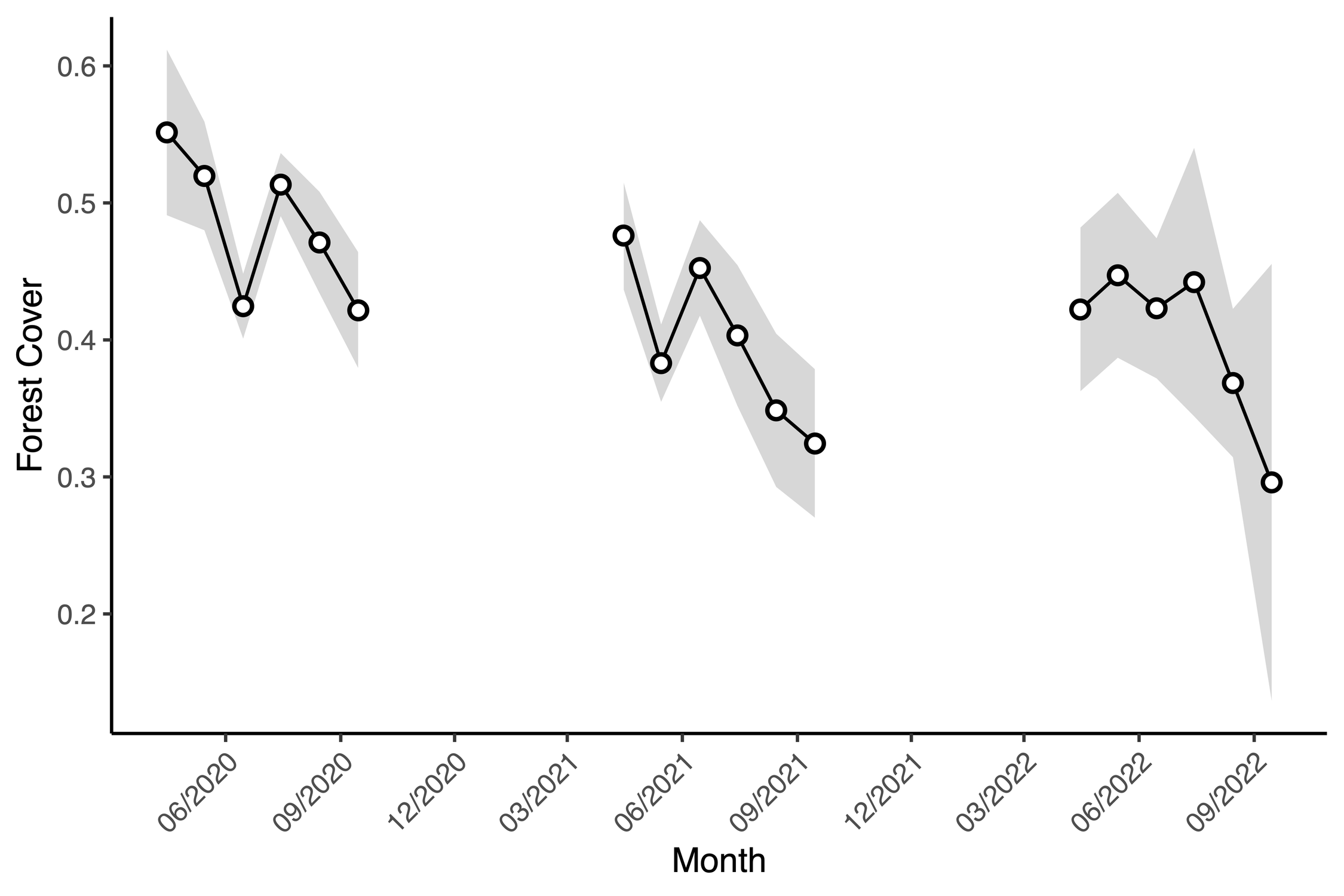

Supplement: S4 Fig — Active bear season is defined yearly as April 15th to October 15th. Month averages were calculated by designating the 15th of each month as the monthly break. Standard errors are shown in gray. (TIF) [file pone.0303429.s004.tif]
