# Supplementary material for: Influence of an extreme event—the COVID-19 pandemic—On establishment of and data collection by a citizen science project
Source: PLoS One. 2024 May 31;19(5):e0303429. doi: 10.1371/journal.pone.0303429 (PMC11142546; doi:10.1371/journal.pone.0303429)
Supplement: S1 Table — Sightings reported to MassBears Website from 2019–2022. Overall comparisons by year and season were both significant at an alpha level of 0.05 (F = 20.36, p<0.001 and F = 21.79, p<0.001, respectively). (DOCX) [file pone.0303429.s005.docx]

S5.

Season

|  | **diff** | **lwr** | **upr** | **p.adj** |
| --- | --- | --- | --- | --- |
| **spring-autumn** | 1.542208 | -1.79293 | 4.877348 | 0.628088 |
| **summer-autumn** | 7.055944 | 3.766635 | 10.34525 | 5.75E-07 |
| **winter-autumn** | -3.06585 | -6.5975 | 0.465797 | 0.113627 |
| **summer-spring** | 5.513736 | 2.316623 | 8.71085 | 8.07E-05 |
| **winter-spring** | -4.60806 | -8.054 | -1.16212 | 0.003646 |
| **winter-summer** | -10.1218 | -13.5234 | -6.72019 | 4.98E-12 |

Year

|  | **diff** | **lwr** | **upr** | **p.adj** |
| --- | --- | --- | --- | --- |
| **2020-2019** | 9.813479 | 6.315909 | 13.31105 | 6.40E-11 |
| **2021-2019** | 7.813942 | 4.303022 | 11.32486 | 2.05E-07 |
| **2022-2019** | 4.947089 | 1.310031 | 8.584147 | 0.002975 |
| **2021-2020** | -1.99954 | -5.13392 | 1.134846 | 0.350992 |
| **2022-2020** | -4.86639 | -8.14145 | -1.59133 | 0.000928 |
| **2022-2021** | -2.86685 | -6.15616 | 0.422456 | 0.111381 |
