# Supplementary material for: Influence of an extreme event—the COVID-19 pandemic—On establishment of and data collection by a citizen science project
Source: PLoS One. 2024 May 31;19(5):e0303429. doi: 10.1371/journal.pone.0303429 (PMC11142546; doi:10.1371/journal.pone.0303429)
Supplement: S2 Table — Sightings reported to MassBears Website from 2020–2022. Overall comparisons by year were significant at an alpha level of 0.05 (F = 6.047, p = 0.002). (DOCX) [file pone.0303429.s006.docx]

S6.

| year | difference | lower | upper | adjusted p-value |
| --- | --- | --- | --- | --- |
| 2021-2020 | -0.069 | -0.1203694 | -0.0176307 | 0.00474888 |
| 2022-2020 | -0.0643119 | -0.128367 | -2.57E-04 | 0.04882737 |
| 2022-2021 | 0.00468818 | -0.0641299 | 0.07350631 | 0.9859961 |
