# Supplementary material for: Influence of an extreme event—the COVID-19 pandemic—On establishment of and data collection by a citizen science project
Source: PLoS One. 2024 May 31;19(5):e0303429. doi: 10.1371/journal.pone.0303429 (PMC11142546; doi:10.1371/journal.pone.0303429)
Supplement: S1 Data — “MassMammals Website Visitors” and “MassBears Website Visitors” were used to analyze monthly visitation to websites. “Volunteer recruitment and motivation: MassMammals registered volunteers” includes anonymized coded responses to the registered volunteer form for MassMammals and is used to analyze their recruitment source and motivations. “Dates of volunteer reported mammal sightings (MassMammals)” indicate dates of mammal sightings reported to the MassMammals website. “Volunteer reported location of bear sightings (MassBears)” includes anonymized sighting submissions to MassBears, including date, self-reported location type, unique email ID, and source of recruitment, and is used to analyze level of participation and recruitment source and spatial biases in self-reported location. “Date of sightings and corresponding percent forest cover” is used to analyze spatial biases in forest cover of sightings due to the pandemic. (DOCX) [file pone.0303429.s007.docx]

S3 Anonymized Data

MassMammals Website Visitors

| **month** | **users** |
| --- | --- |
| 6/1/2020 | 22 |
| 7/1/2020 | 292 |
| 8/1/2020 | 105 |
| 9/1/2020 | 16 |
| 10/1/2020 | 67 |
| 11/1/2020 | 19 |
| 12/1/2020 | 9 |
| 1/1/2021 | 48 |
| 2/1/2021 | 49 |
| 3/1/2021 | 90 |
| 4/1/2021 | 135 |
| 5/1/2021 | 159 |
| 6/1/2021 | 213 |
| 7/1/2021 | 306 |
| 8/1/2021 | 334 |
| 9/1/2021 | 419 |
| 10/1/2021 | 410 |
| 11/1/2021 | 292 |
| 12/1/2021 | 335 |
| 1/1/2022 | 418 |
| 2/1/2022 | 434 |
| 3/1/2022 | 472 |
| 4/1/2022 | 498 |
| 5/1/2022 | 588 |
| 6/1/2022 | 643 |
| 7/1/2022 | 688 |
| 8/1/2022 | 653 |
| 9/1/2022 | 712 |
| 10/1/2022 | 383 |

MassBears Website Visitors

| **month** | **users** |
| --- | --- |
| 6/1/2019 | 106 |
| 7/1/2019 | 23 |
| 8/1/2019 | 41 |
| 9/1/2019 | 19 |
| 10/1/2019 | 18 |
| 11/1/2019 | 29 |
| 12/1/2019 | 10 |
| 1/1/2020 | 17 |
| 2/1/2020 | 8 |
| 3/1/2020 | 3 |
| 4/1/2020 | 13 |
| 5/1/2020 | 12 |
| 6/1/2020 | 26 |
| 7/1/2020 | 122 |
| 8/1/2020 | 44 |
| 9/1/2020 | 21 |
| 10/1/2020 | 26 |
| 11/1/2020 | 14 |
| 12/1/2020 | 9 |
| 1/1/2021 | 8 |
| 2/1/2021 | 11 |
| 3/1/2021 | 18 |
| 4/1/2021 | 29 |
| 5/1/2021 | 96 |
| 6/1/2021 | 91 |
| 7/1/2021 | 40 |
| 8/1/2021 | 32 |
| 9/1/2021 | 36 |
| 10/1/2021 | 54 |
| 11/1/2021 | 54 |
| 12/1/2021 | 28 |
| 1/1/2022 | 38 |
| 2/1/2022 | 38 |
| 3/1/2022 | 51 |
| 4/1/2022 | 53 |
| 5/1/2022 | 174 |
| 6/1/2022 | 98 |
| 7/1/2022 | 102 |
| 8/1/2022 | 91 |
| 9/1/2022 | 388 |
| 10/1/2022 | 48 |

Volunteer recruitment and motivation: MassMammals registered volunteers

| **volunteer_id** | **Recruitment Categories** | **Motivation Categories** |  |
| --- | --- | --- | --- |
| V001 | Friend/community | learning about nearby wildlife |  |
| V002 | Internet Search/Amherst College | contributing to conservation research |  |
| V003 | Internet Search/Amherst College | REPEAT |  |
| V004 | Internet Search/Amherst College | contributing to conservation research |  |
| V005 | Newspaper | experience observing wildlife |  |
| V006 | Newspaper | contributing to conservation research | protecting/coexisting with wildlife |
| V007 | Newspaper | contributing to conservation research |  |
| V008 | Newspaper | contributing to conservation research | protecting/coexisting with wildlife |
| V009 | Newspaper | learning about nearby wildlife |  |
| V010 | Newspaper |  |  |
| V011 | Internet Search/Amherst College | contributing to conservation research | learning about nearby wildlife |
| V012 | Project Member/Contributor | experience observing wildlife |  |
| V013 | Newspaper | experience observing wildlife |  |
| V014 | Newspaper | learning about nearby wildlife |  |
| V015 | Newspaper | contributing to conservation research | protecting/coexisting with wildlife |
| V016 | Newspaper | protecting/coexisting with wildlife | experience observing wildlife |
| V017 | Newspaper | love for nature, animals, wildlife protection |  |
| V018 | Social media | love for nature, animals, wildlife protection |  |
| V019 | Social media | learning about nearby wildlife |  |
| V020 | Social media | experience observing wildlife | contributing to conservation research |
| V021 | Social media | love for nature, animals, wildlife protection |  |
| V022 | Social media | contributing to conservation research |  |
| V023 | Social media |  |  |
| V024 | Social media | experience observing wildlife |  |
| V025 | Social media | contributing to conservation research |  |
| V026 | Social media | experience observing wildlife | contributing to conservation research |
| V027 | Social media | love for nature, animals, wildlife protection | learning about nearby wildlife |
| V028 | Social media | love for nature, animals, wildlife protection |  |
| V029 | Newspaper | experience observing wildlife |  |
| V030 | Friend/community | learning about nearby wildlife |  |
| V031 | Internet Search/Amherst College | love for nature, animals, wildlife protection |  |
| V032 | Newspaper |  |  |
| V033 | Newspaper | contributing to conservation research | learning about nearby wildlife |
| V034 | Newspaper | love for nature, animals, wildlife protection |  |
| V035 | Friend/community | love for nature, animals, wildlife protection |  |
| V036 | Internet Search/Amherst College | protecting/coexisting with wildlife |  |
| V037 | Internet Search/Amherst College | love for nature, animals, wildlife protection | learning about nearby wildlife |
| V038 | Newspaper | love for nature, animals, wildlife protection |  |
| V039 | Newspaper | learning about nearby wildlife |  |
| V040 | Internet Search/Amherst College | love for nature, animals, wildlife protection | protecting/coexisting with wildlife |
| V041 | Internet Search/Amherst College | contributing to conservation research |  |
| V042 | Newspaper | love for nature, animals, wildlife protection |  |
| V043 | Internet Search/Amherst College | experience observing wildlife | learning about nearby wildlife |
| V044 | Internet Search/Amherst College | experience observing wildlife | protecting/coexisting with wildlife |
| V045 | Internet Search/Amherst College | love for nature, animals, wildlife protection | learning about nearby wildlife |
| V046 | Social media |  |  |
| V047 | Project Member/Contributor | love for nature, animals, wildlife protection | protecting/coexisting with wildlife |
| V048 | Social media | learning about nearby wildlife |  |
| V049 | Newspaper | learning about nearby wildlife | contributing to conservation research |
| V050 | Newspaper | experience observing wildlife | protecting/coexisting with wildlife |
| V051 | Newspaper | love for nature, animals, wildlife protection | learning about nearby wildlife |
| V052 | Newspaper | contributing to conservation research |  |
| V053 | Friend/community | learning about nearby wildlife |  |
| V054 | Newspaper | contributing to conservation research |  |
| V055 | Newspaper | love for nature, animals, wildlife protection |  |
| V056 | Newspaper | learning about nearby wildlife |  |
| V057 | Newspaper | protecting/coexisting with wildlife |  |
| V058 | Newspaper | protecting/coexisting with wildlife | learning about nearby wildlife |
| V059 | Newspaper | learning about nearby wildlife |  |
| V060 | Newspaper | contributing to conservation research | experience observing wildlife |
| V061 | Social media | protecting/coexisting with wildlife | learning about nearby wildlife |
| V062 | Social media | contributing to conservation research | protecting/coexisting with wildlife |
| V063 | Newspaper | contributing to conservation research |  |
| V064 | Internet Search/Amherst College | contributing to conservation research | protecting/coexisting with wildlife |
| V065 | Internet Search/Amherst College | learning about nearby wildlife |  |
| V066 | NA | contributing to conservation research |  |
| V067 | Internet Search/Amherst College | protecting/coexisting with wildlife | learning about nearby wildlife |
| V068 | Project Member/Contributor | learning about nearby wildlife |  |
| V069 | Newspaper |  |  |
| V070 | Newspaper | learning about nearby wildlife |  |
| V071 | Project Member/Contributor | contributing to conservation research | protecting/coexisting with wildlife |
| V072 | Internet Search/Amherst College | learning about nearby wildlife |  |
| V073 | Social media | experience observing wildlife | learning about nearby wildlife |
| V074 | Internet Search/Amherst College | contributing to conservation research |  |
| V075 | Internet Search/Amherst College | contributing to conservation research | protecting/coexisting with wildlife |
| V076 | Friend/community | love for nature, animals, wildlife protection |  |
| V077 | Internet Search/Amherst College | contributing to conservation research |  |
| V078 | Social media | love for nature, animals, wildlife protection |  |
| V079 | Social media | learning about nearby wildlife | protecting/coexisting with wildlife |
| V080 | Internet Search/Amherst College | protecting/coexisting with wildlife |  |
| V081 | Internet Search/Amherst College | learning about nearby wildlife |  |
| V082 | Internet Search/Amherst College | contributing to conservation research |  |
| V083 | Newspaper | experience observing wildlife | learning about nearby wildlife |
| V084 | Newspaper | protecting/coexisting with wildlife |  |
| V085 | Newspaper | contributing to conservation research | protecting/coexisting with wildlife |
| V086 | Internet Search/Amherst College | experience observing wildlife | contributing to conservation research |
| V087 |  | protecting/coexisting with wildlife |  |
| V088 | Internet Search/Amherst College | experience observing wildlife | learning about nearby wildlife |
| V089 | Internet Search/Amherst College | protecting/coexisting with wildlife |  |
| V090 | Internet Search/Amherst College | contributing to conservation research | protecting/coexisting with wildlife |
| V091 | Internet Search/Amherst College | experience observing wildlife | contributing to conservation research |
| V092 | Internet Search/Amherst College | protecting/coexisting with wildlife |  |
| V093 |  | protecting/coexisting with wildlife |  |
| V094 | Social media | learning about nearby wildlife |  |

**Dates of volunteer reported mammal sightings (MassMammals)**

| Date_of_sighting |
| --- |
| 6/12/2020 |
| 7/5/2020 |
| 9/8/2020 |
| 1/20/2021 |
| 3/13/2021 |
| 3/23/2021 |
| 1/29/2021 |
| 12/6/2020 |
| 10/30/2020 |
| 1/22/2021 |
| 9/9/2019 |
| 3/14/2021 |
| 4/14/2021 |
| 4/12/2021 |
| 5/7/2021 |
| 7/12/2020 |
| 6/25/2020 |
| 4/19/2021 |
| 4/24/2021 |
| 4/28/2021 |
| 4/17/2020 |
| 4/28/2021 |
| 3/31/2021 |
| 5/7/2021 |
| 12/12/2020 |
| 7/17/2020 |
| 3/24/2021 |
| 3/9/2021 |
| 7/13/2020 |
| 3/10/2021 |
| 3/4/2021 |
| 3/6/2021 |
| 3/14/2021 |
| 8/25/2021 |
| 8/21/2021 |
| 5/27/2021 |
| 6/15/2021 |
| 10/26/2021 |
| 11/4/2021 |
| 6/25/2021 |
| 5/20/2021 |
| 5/26/2021 |
| 1/20/2021 |
| 6/1/2021 |
| 6/7/2021 |
| 5/28/2021 |
| 6/7/2021 |
| 6/6/2021 |
| 6/6/2021 |
| 6/14/2021 |
| 6/20/2021 |
| 7/20/2021 |
| 7/22/2021 |
| 7/29/2021 |
| 8/5/2021 |
| 8/16/2021 |
| 8/29/2021 |
| 9/3/2021 |
| 10/30/2021 |
| 10/22/2021 |
| 11/1/2021 |
| 11/3/2021 |
| 5/27/2021 |
| 6/7/2021 |
| 5/28/2021 |
| 5/31/2021 |
| 5/23/2021 |
| 6/18/2021 |
| 6/9/2021 |
| 6/10/2021 |
| 6/1/2021 |
| 6/18/2021 |
| 8/16/2021 |
| 6/1/2021 |
| 6/24/2021 |
| 6/20/2021 |
| 8/16/2021 |
| 8/5/2021 |
| 7/29/2021 |
| 10/22/2021 |
| 1/2/2022 |
| 2/16/2022 |
| 2/11/2022 |
| 2/22/2022 |
| 1/29/2022 |
| 3/9/2022 |
| 3/16/2022 |
| 3/18/2022 |
| 3/14/2022 |
| 1/22/2022 |
| 3/31/2022 |
| 3/30/2022 |
| 3/31/2022 |
| 4/14/2022 |
| 6/25/2020 |
| 10/24/2020 |
| 10/25/2020 |
| 11/16/2021 |
| 4/24/2022 |
| 5/6/2022 |
| 4/23/2022 |
| 5/3/2022 |
| 5/29/2022 |
| 6/25/2022 |
| 6/24/2022 |
| 6/21/2022 |
| 7/2/2022 |
| 6/29/2022 |
| 7/5/2022 |
| 7/4/2022 |
| 7/6/2022 |
| 7/14/2022 |
| 7/19/2022 |
| 7/26/2022 |
| 8/3/2022 |
| 8/5/2022 |
| 8/11/2022 |
| 8/21/2022 |
| 8/27/2022 |
| 8/29/2022 |
| 9/7/2022 |
| 9/16/2022 |
| 9/19/2022 |

**Volunteer reported location of bear sightings and recruitment category (MassBears)**

| sighting_id | date | location_redacted | email_id | recruitment_category |
| --- | --- | --- | --- | --- |
| S0001 | 2019-04-01 | Yard or neighborhood | E0009 | NA |
| S0002 | 2019-04-08 | Yard or neighborhood | E0234 | NA |
| S0003 | 2019-04-08 | Yard or neighborhood | E0234 | NA |
| S0004 | 2019-04-25 | Yard or neighborhood | E0047 | Social media |
| S0005 | 2019-05-18 | Yard or neighborhood | E0009 | NA |
| S0006 | 2019-06-09 | Yard or neighborhood | E0391 | NA |
| S0007 | 2019-06-12 | Yard or neighborhood | E0024 | NA |
| S0008 | 2019-06-12 | Yard or neighborhood | E0317 | NA |
| S0009 | 2019-06-12 | Yard or neighborhood | NA | NA |
| S0010 | 2019-06-13 | Yard or neighborhood | E0172 | NA |
| S0011 | 2019-06-14 | OTHER | E0117 | NA |
| S0012 | 2019-06-14 | Yard or neighborhood | E0413 | NA |
| S0013 | 2019-06-14 | Yard or neighborhood | NA | NA |
| S0014 | 2019-06-17 | Yard or neighborhood | E0602 | NA |
| S0015 | 2019-06-19 | Yard or neighborhood | E0144 | NA |
| S0016 | 2019-06-22 | Yard or neighborhood | NA | NA |
| S0017 | 2019-07-23 | Yard or neighborhood | E0424 | Social media |
| S0018 | 2019-07-25 | Yard or neighborhood | E0018 | NA |
| S0019 | 2019-07-25 | Yard or neighborhood | E0318 | NA |
| S0020 | 2019-07-26 | Yard or neighborhood | E0555 | NA |
| S0021 | 2019-08-06 | Yard or neighborhood | E0326 | NA |
| S0022 | 2019-08-07 | Yard or neighborhood | E0177 | NA |
| S0023 | 2019-08-14 | Yard or neighborhood | E0393 | NA |
| S0024 | 2019-10-01 | Yard or neighborhood | E0496 | Social media |
| S0025 | 2020-01-07 | OTHER | NA | NA |
| S0026 | 2020-04-12 | Yard or neighborhood | E0088 | NA |
| S0027 | 2020-04-24 | OTHER | E0447 | NA |
| S0028 | 2020-04-25 | Yard or neighborhood | NA | NA |
| S0029 | 2020-04-25 | Forest | E0461 | Newspaper |
| S0030 | 2020-04-26 | OTHER | E0557 | NA |
| S0031 | 2020-04-28 | Yard or neighborhood | E0577 | NA |
| S0032 | 2020-04-28 | Yard or neighborhood | E0577 | NA |
| S0033 | 2020-05-02 | Yard or neighborhood | E0234 | NA |
| S0034 | 2020-05-02 | Yard or neighborhood | E0326 | NA |
| S0035 | 2020-05-03 | Yard or neighborhood | E0085 | Social media |
| S0036 | 2020-05-03 | Yard or neighborhood | E0085 | Social media |
| S0037 | 2020-05-03 | Forest | E0401 | NA |
| S0038 | 2020-05-04 | Yard or neighborhood | E0039 | Social media |
| S0039 | 2020-05-06 | Business | E0017 | Social media |
| S0040 | 2020-05-06 | Business | E0127 | Internet Search/Amherst College |
| S0041 | 2020-05-06 | Yard or neighborhood | E0367 | Newspaper |
| S0042 | 2020-05-09 | Forest | E0608 | Newspaper |
| S0043 | 2020-05-10 | Yard or neighborhood | E0345 | NA |
| S0044 | 2020-05-11 | Forest | NA | NA |
| S0045 | 2020-05-12 | Yard or neighborhood | E0423 | Newspaper |
| S0046 | 2020-05-13 | Forest | E0491 | NA |
| S0047 | 2020-05-14 | Yard or neighborhood | NA | NA |
| S0048 | 2020-05-15 | Yard or neighborhood | E0069 | Newspaper |
| S0049 | 2020-05-15 | Yard or neighborhood | E0109 | NA |
| S0050 | 2020-05-15 | Yard or neighborhood | E0123 | Friend/community |
| S0051 | 2020-05-15 | Forest | NA | NA |
| S0052 | 2020-05-16 | Yard or neighborhood | E0338 | NA |
| S0053 | 2020-05-17 | Yard or neighborhood | E0102 | Newspaper |
| S0054 | 2020-05-17 | Yard or neighborhood | E0155 | NA |
| S0055 | 2020-05-17 | Yard or neighborhood | E0155 | NA |
| S0056 | 2020-05-17 | Yard or neighborhood | E0387 | NA |
| S0057 | 2020-05-18 | OTHER | E0181 | NA |
| S0058 | 2020-05-18 | Yard or neighborhood | E0244 | NA |
| S0059 | 2020-05-19 | Yard or neighborhood | E0069 | Newspaper |
| S0060 | 2020-05-19 | Yard or neighborhood | E0446 | Social media |
| S0061 | 2020-05-21 | Yard or neighborhood | E0178 | NA |
| S0062 | 2020-05-21 | Yard or neighborhood | E0216 | Newspaper |
| S0063 | 2020-05-21 | Yard or neighborhood | NA | NA |
| S0064 | 2020-05-22 | Yard or neighborhood | E0069 | Newspaper |
| S0065 | 2020-05-22 | Yard or neighborhood | E0372 | Social media |
| S0066 | 2020-05-22 | Yard or neighborhood | E0481 | NA |
| S0067 | 2020-05-23 | Yard or neighborhood | E0372 | Social media |
| S0068 | 2020-05-23 | OTHER | NA | NA |
| S0069 | 2020-05-24 | OTHER | E0253 | NA |
| S0070 | 2020-05-24 | Forest | E0336 | NA |
| S0071 | 2020-05-24 | Yard or neighborhood | E0374 | NA |
| S0072 | 2020-05-25 | Forest | E0361 | NA |
| S0073 | 2020-05-25 | OTHER | NA | NA |
| S0074 | 2020-05-26 | OTHER | E0032 | NA |
| S0075 | 2020-05-26 | Forest | E0228 | NA |
| S0076 | 2020-05-26 | Yard or neighborhood | NA | NA |
| S0077 | 2020-05-26 | Yard or neighborhood | E0608 | Newspaper |
| S0078 | 2020-05-27 | Yard or neighborhood | E0216 | Newspaper |
| S0079 | 2020-05-27 | Forest | E0224 | NA |
| S0080 | 2020-05-28 | Yard or neighborhood | E0164 | NA |
| S0081 | 2020-05-31 | Yard or neighborhood | E0069 | Newspaper |
| S0082 | 2020-05-31 | Yard or neighborhood | E0211 | Social media |
| S0083 | 2020-06-01 | Yard or neighborhood | E0069 | NA |
| S0084 | 2020-06-01 | OTHER | NA | NA |
| S0085 | 2020-06-02 | Yard or neighborhood | E0216 | Newspaper |
| S0086 | 2020-06-02 | Yard or neighborhood | NA | Newspaper |
| S0087 | 2020-06-05 | OTHER | E0058 | NA |
| S0088 | 2020-06-05 | Yard or neighborhood | E0058 | NA |
| S0089 | 2020-06-06 | Yard or neighborhood | E0464 | Social media |
| S0090 | 2020-06-07 | OTHER | E0059 | NA |
| S0091 | 2020-06-08 | Yard or neighborhood | E0148 | Newspaper |
| S0092 | 2020-06-08 | Yard or neighborhood | E0148 | Newspaper |
| S0093 | 2020-06-08 | Yard or neighborhood | E0305 | Internet Search/Amherst College |
| S0094 | 2020-06-08 | Yard or neighborhood | E0365 | Social media |
| S0095 | 2020-06-08 | Yard or neighborhood | E0410 | Newspaper |
| S0096 | 2020-06-09 | Yard or neighborhood | E0141 | Newspaper |
| S0097 | 2020-06-09 | Yard or neighborhood | E0189 | NA |
| S0098 | 2020-06-09 | Yard or neighborhood | E0262 | Newspaper |
| S0099 | 2020-06-09 | Yard or neighborhood | E0305 | Internet Search/Amherst College |
| S0100 | 2020-06-10 | Yard or neighborhood | E0192 | Newspaper |
| S0101 | 2020-06-10 | Yard or neighborhood | NA | NA |
| S0102 | 2020-06-10 | Yard or neighborhood | E0564 | NA |
| S0103 | 2020-06-10 | Yard or neighborhood | E0593 | Social media |
| S0104 | 2020-06-12 | Yard or neighborhood | E0148 | Newspaper |
| S0105 | 2020-06-12 | Yard or neighborhood | E0148 | Newspaper |
| S0106 | 2020-06-13 | Yard or neighborhood | E0058 | NA |
| S0107 | 2020-06-13 | Yard or neighborhood | E0213 | Newspaper |
| S0108 | 2020-06-13 | Yard or neighborhood | NA | NA |
| S0109 | 2020-06-14 | Yard or neighborhood | E0069 | Newspaper |
| S0110 | 2020-06-14 | Yard or neighborhood | E0420 | Friend/community |
| S0111 | 2020-06-15 | Yard or neighborhood | E0122 | Newspaper |
| S0112 | 2020-06-15 | Yard or neighborhood | E0248 | NA |
| S0113 | 2020-06-15 | Yard or neighborhood | E0278 | Friend/community |
| S0114 | 2020-06-15 | Yard or neighborhood | E0294 | Social media |
| S0115 | 2020-06-15 | Forest | E0306 | Social media |
| S0116 | 2020-06-17 | Yard or neighborhood | E0301 | Internet Search/Amherst College |
| S0117 | 2020-06-17 | Yard or neighborhood | E0522 | Newspaper |
| S0118 | 2020-06-17 | Yard or neighborhood | E0522 | Newspaper |
| S0119 | 2020-06-18 | Yard or neighborhood | E0054 | Social media |
| S0120 | 2020-06-18 | Yard or neighborhood | E0058 | NA |
| S0121 | 2020-06-18 | Yard or neighborhood | E0069 | Newspaper |
| S0122 | 2020-06-18 | Yard or neighborhood | E0500 | Internet Search/Amherst College |
| S0123 | 2020-06-19 | Yard or neighborhood | E0298 | Friend/community |
| S0124 | 2020-06-19 | Yard or neighborhood | E0583 | NA |
| S0125 | 2020-06-20 | OTHER | E0143 | Social media |
| S0126 | 2020-06-20 | Forest | E0415 | Social media |
| S0127 | 2020-06-21 | Yard or neighborhood | E0283 | Newspaper |
| S0128 | 2020-06-21 | Yard or neighborhood | NA | NA |
| S0129 | 2020-06-21 | Yard or neighborhood | E0468 | Social media |
| S0130 | 2020-06-22 | OTHER | E0058 | NA |
| S0131 | 2020-06-22 | Yard or neighborhood | E0606 | NA |
| S0132 | 2020-06-23 | Yard or neighborhood | E0257 | Friend/community |
| S0133 | 2020-06-23 | Yard or neighborhood | E0345 | Newspaper |
| S0134 | 2020-06-23 | Yard or neighborhood | E0404 | NA |
| S0135 | 2020-06-24 | OTHER | E0058 | NA |
| S0136 | 2020-06-24 | Yard or neighborhood | E0337 | Social media |
| S0137 | 2020-06-24 | Yard or neighborhood | E0388 | Newspaper |
| S0138 | 2020-06-24 | Street | E0392 | Social media |
| S0139 | 2020-06-24 | Yard or neighborhood | NA | NA |
| S0140 | 2020-06-24 | Yard or neighborhood | E0599 | Internet Search/Amherst College |
| S0141 | 2020-06-25 | Yard or neighborhood | E0074 | Newspaper |
| S0142 | 2020-06-25 | OTHER | E0246 | Friend/community |
| S0143 | 2020-06-25 | Yard or neighborhood | NA | NA |
| S0144 | 2020-06-26 | Yard or neighborhood | E0058 | NA |
| S0145 | 2020-06-26 | Yard or neighborhood | E0398 | Social media |
| S0146 | 2020-06-26 | Yard or neighborhood | E0410 | Newspaper |
| S0147 | 2020-06-26 | Yard or neighborhood | NA | NA |
| S0148 | 2020-06-26 | Yard or neighborhood | E0501 | Social media |
| S0149 | 2020-06-26 | Yard or neighborhood | E0558 | Newspaper |
| S0150 | 2020-06-27 | Yard or neighborhood | E0028 | Social media |
| S0151 | 2020-06-27 | Yard or neighborhood | E0154 | Internet Search/Amherst College |
| S0152 | 2020-06-27 | Yard or neighborhood | NA | NA |
| S0153 | 2020-06-28 | OTHER | E0058 | NA |
| S0154 | 2020-06-28 | Yard or neighborhood | NA | NA |
| S0155 | 2020-06-29 | Yard or neighborhood | E0455 | Social media |
| S0156 | 2020-06-30 | Yard or neighborhood | E0050 | Social media |
| S0157 | 2020-06-30 | Yard or neighborhood | E0245 | Social media |
| S0158 | 2020-06-30 | Yard or neighborhood | E0510 | Social media |
| S0159 | 2020-07-01 | Yard or neighborhood | E0014 | Friend/community |
| S0160 | 2020-07-01 | Yard or neighborhood | E0210 | Social media |
| S0161 | 2020-07-01 | Yard or neighborhood | E0247 | Friend/community |
| S0162 | 2020-07-01 | Yard or neighborhood | E0313 | NA |
| S0163 | 2020-07-01 | Yard or neighborhood | E0313 | NA |
| S0164 | 2020-07-01 | OTHER | E0354 | Friend/community |
| S0165 | 2020-07-02 | Yard or neighborhood | E0092 | Newspaper |
| S0166 | 2020-07-02 | Yard or neighborhood | E0146 | Newspaper |
| S0167 | 2020-07-02 | Yard or neighborhood | E0419 | NA |
| S0168 | 2020-07-02 | Yard or neighborhood | E0464 | Social media |
| S0169 | 2020-07-03 | OTHER | E0236 | Social media |
| S0170 | 2020-07-03 | Yard or neighborhood | E0410 | Newspaper |
| S0171 | 2020-07-03 | Yard or neighborhood | NA | NA |
| S0172 | 2020-07-03 | OTHER | E0518 | Newspaper |
| S0173 | 2020-07-04 | Yard or neighborhood | E0020 | Newspaper |
| S0174 | 2020-07-04 | Yard or neighborhood | NA | NA |
| S0175 | 2020-07-04 | Yard or neighborhood | E0583 | Social media |
| S0176 | 2020-07-05 | Yard or neighborhood | E0020 | Newspaper |
| S0177 | 2020-07-05 | Yard or neighborhood | E0210 | NA |
| S0178 | 2020-07-05 | Yard or neighborhood | E0274 | Newspaper |
| S0179 | 2020-07-05 | Yard or neighborhood | E0429 | NA |
| S0180 | 2020-07-06 | Yard or neighborhood | E0053 | Newspaper |
| S0181 | 2020-07-06 | Yard or neighborhood | E0131 | Newspaper |
| S0182 | 2020-07-06 | Yard or neighborhood | E0386 | NA |
| S0183 | 2020-07-07 | Yard or neighborhood | E0388 | Newspaper |
| S0184 | 2020-07-07 | Yard or neighborhood | E0432 | Newspaper |
| S0185 | 2020-07-07 | Yard or neighborhood | E0549 | Newspaper |
| S0186 | 2020-07-07 | Forest | E0553 | Newspaper |
| S0187 | 2020-07-07 | Yard or neighborhood | E0603 | Newspaper |
| S0188 | 2020-07-08 | Yard or neighborhood | E0127 | Internet Search/Amherst College |
| S0189 | 2020-07-08 | OTHER | E0273 | Friend/community |
| S0190 | 2020-07-08 | Yard or neighborhood | NA | Newspaper |
| S0191 | 2020-07-08 | Yard or neighborhood | NA | NA |
| S0192 | 2020-07-08 | Yard or neighborhood | E0533 | Social media |
| S0193 | 2020-07-08 | Park | E0546 | Newspaper |
| S0194 | 2020-07-08 | OTHER | E0584 | Social media |
| S0195 | 2020-07-09 | Yard or neighborhood | E0095 | Social media |
| S0196 | 2020-07-09 | Yard or neighborhood | E0240 | Social media |
| S0197 | 2020-07-09 | Yard or neighborhood | E0365 | Social media |
| S0198 | 2020-07-09 | Yard or neighborhood | E0379 | Newspaper |
| S0199 | 2020-07-09 | Yard or neighborhood | E0490 | NA |
| S0200 | 2020-07-10 | OTHER | E0128 | Newspaper |
| S0201 | 2020-07-10 | Yard or neighborhood | E0212 | Newspaper |
| S0202 | 2020-07-10 | Yard or neighborhood | E0239 | Newspaper |
| S0203 | 2020-07-10 | Yard or neighborhood | E0283 | Newspaper |
| S0204 | 2020-07-10 | Yard or neighborhood | E0378 | Newspaper |
| S0205 | 2020-07-10 | Yard or neighborhood | E0537 | Newspaper |
| S0206 | 2020-07-10 | Yard or neighborhood | E0571 | Newspaper |
| S0207 | 2020-07-10 | Forest | E0611 | Friend/community |
| S0208 | 2020-07-11 | Yard or neighborhood | E0029 | Newspaper |
| S0209 | 2020-07-11 | Yard or neighborhood | E0378 | Newspaper |
| S0210 | 2020-07-11 | Yard or neighborhood | E0439 | Friend/community |
| S0211 | 2020-07-12 | Yard or neighborhood | E0085 | NA |
| S0212 | 2020-07-12 | Yard or neighborhood | E0242 | Friend/community |
| S0213 | 2020-07-12 | Yard or neighborhood | E0302 | Social media |
| S0214 | 2020-07-12 | Yard or neighborhood | E0341 | Social media |
| S0215 | 2020-07-12 | Yard or neighborhood | E0574 | Social media |
| S0216 | 2020-07-13 | Yard or neighborhood | E0075 | Newspaper |
| S0217 | 2020-07-13 | Yard or neighborhood | E0121 | Newspaper |
| S0218 | 2020-07-13 | Yard or neighborhood | E0132 | Newspaper |
| S0219 | 2020-07-13 | Yard or neighborhood | E0134 | Newspaper |
| S0220 | 2020-07-13 | OTHER | E0463 | Newspaper |
| S0221 | 2020-07-14 | OTHER | E0251 | Newspaper |
| S0222 | 2020-07-14 | Yard or neighborhood | E0285 | Newspaper |
| S0223 | 2020-07-14 | Yard or neighborhood | E0490 | NA |
| S0224 | 2020-07-15 | Yard or neighborhood | E0216 | Newspaper |
| S0225 | 2020-07-15 | Yard or neighborhood | E0352 | NA |
| S0226 | 2020-07-15 | Yard or neighborhood | NA | NA |
| S0227 | 2020-07-15 | Yard or neighborhood | E0476 | Newspaper |
| S0228 | 2020-07-15 | Yard or neighborhood | E0534 | NA |
| S0229 | 2020-07-16 | Yard or neighborhood | E0147 | Friend/community |
| S0230 | 2020-07-16 | Yard or neighborhood | E0484 | Newspaper |
| S0231 | 2020-07-16 | OTHER | E0525 | Newspaper |
| S0232 | 2020-07-17 | Yard or neighborhood | E0013 | Newspaper |
| S0233 | 2020-07-17 | Yard or neighborhood | E0013 | Newspaper |
| S0234 | 2020-07-17 | Yard or neighborhood | E0067 | Social media |
| S0235 | 2020-07-17 | Yard or neighborhood | E0156 | Newspaper |
| S0236 | 2020-07-17 | Yard or neighborhood | E0170 | Internet Search/Amherst College |
| S0237 | 2020-07-17 | Yard or neighborhood | E0450 | Friend/community |
| S0238 | 2020-07-17 | Yard or neighborhood | E0476 | NA |
| S0239 | 2020-07-17 | Yard or neighborhood | E0595 | NA |
| S0240 | 2020-07-17 | Yard or neighborhood | E0598 | Social media |
| S0241 | 2020-07-18 | Yard or neighborhood | E0103 | Newspaper |
| S0242 | 2020-07-18 | Yard or neighborhood | E0190 | Newspaper |
| S0243 | 2020-07-18 | OTHER | E0466 | Newspaper |
| S0244 | 2020-07-18 | Forest | E0577 | Newspaper |
| S0245 | 2020-07-18 | Yard or neighborhood | E0606 | Newspaper |
| S0246 | 2020-07-18 | Yard or neighborhood | E0606 | Newspaper |
| S0247 | 2020-07-19 | Yard or neighborhood | E0149 | Newspaper |
| S0248 | 2020-07-19 | Forest | E0228 | NA |
| S0249 | 2020-07-19 | Yard or neighborhood | E0287 | Newspaper |
| S0250 | 2020-07-19 | Yard or neighborhood | E0303 | Friend/community |
| S0251 | 2020-07-19 | Yard or neighborhood | E0406 | Friend/community |
| S0252 | 2020-07-19 | Yard or neighborhood | NA | Social media |
| S0253 | 2020-07-20 | Yard or neighborhood | E0058 | NA |
| S0254 | 2020-07-20 | Yard or neighborhood | E0133 | Friend/community |
| S0255 | 2020-07-20 | Yard or neighborhood | E0141 | Newspaper |
| S0256 | 2020-07-20 | Yard or neighborhood | E0221 | NA |
| S0257 | 2020-07-20 | Yard or neighborhood | E0321 | Social media |
| S0258 | 2020-07-21 | Forest | E0026 | Newspaper |
| S0259 | 2020-07-21 | Yard or neighborhood | E0046 | Newspaper |
| S0260 | 2020-07-21 | Forest | E0056 | Newspaper |
| S0261 | 2020-07-21 | OTHER | E0164 | Newspaper |
| S0262 | 2020-07-21 | Yard or neighborhood | E0297 | NA |
| S0263 | 2020-07-21 | Yard or neighborhood | E0419 | Newspaper |
| S0264 | 2020-07-22 | Yard or neighborhood | E0058 | NA |
| S0265 | 2020-07-22 | Yard or neighborhood | E0367 | Newspaper |
| S0266 | 2020-07-22 | Yard or neighborhood | E0367 | Newspaper |
| S0267 | 2020-07-22 | Yard or neighborhood | E0367 | Newspaper |
| S0268 | 2020-07-22 | Yard or neighborhood | E0386 | Newspaper |
| S0269 | 2020-07-22 | Yard or neighborhood | E0425 | Social media |
| S0270 | 2020-07-22 | Yard or neighborhood | E0548 | Newspaper |
| S0271 | 2020-07-22 | OTHER | E0570 | x |
| S0272 | 2020-07-23 | Yard or neighborhood | E0012 | Social media |
| S0273 | 2020-07-23 | OTHER | E0058 | NA |
| S0274 | 2020-07-23 | Yard or neighborhood | E0073 | Newspaper |
| S0275 | 2020-07-23 | Yard or neighborhood | E0547 | Friend/community |
| S0276 | 2020-07-23 | Yard or neighborhood | E0610 | Newspaper |
| S0277 | 2020-07-24 | Yard or neighborhood | E0025 | Internet Search/Amherst College |
| S0278 | 2020-07-24 | Yard or neighborhood | E0445 | Newspaper |
| S0279 | 2020-07-25 | Yard or neighborhood | E0056 | Newspaper |
| S0280 | 2020-07-25 | Yard or neighborhood | E0065 | Newspaper |
| S0281 | 2020-07-25 | Forest | E0106 | Newspaper |
| S0282 | 2020-07-25 | Yard or neighborhood | E0356 | Newspaper |
| S0283 | 2020-07-26 | Yard or neighborhood | E0013 | Newspaper |
| S0284 | 2020-07-26 | Yard or neighborhood | E0013 | Newspaper |
| S0285 | 2020-07-26 | Yard or neighborhood | E0013 | Newspaper |
| S0286 | 2020-07-26 | Yard or neighborhood | E0073 | NA |
| S0287 | 2020-07-26 | Yard or neighborhood | E0195 | Social media |
| S0288 | 2020-07-26 | Yard or neighborhood | E0223 | NA |
| S0289 | 2020-07-26 | Yard or neighborhood | NA | NA |
| S0290 | 2020-07-26 | Yard or neighborhood | NA | NA |
| S0291 | 2020-07-26 | Yard or neighborhood | NA | NA |
| S0292 | 2020-07-26 | Yard or neighborhood | E0563 | Friend/community |
| S0293 | 2020-07-27 | Yard or neighborhood | E0278 | Newspaper |
| S0294 | 2020-07-27 | OTHER | E0370 | Social media |
| S0295 | 2020-07-27 | Yard or neighborhood | E0386 | Newspaper |
| S0296 | 2020-07-27 | Yard or neighborhood | E0422 | NA |
| S0297 | 2020-07-27 | Yard or neighborhood | E0428 | Newspaper |
| S0298 | 2020-07-27 | Yard or neighborhood | NA | NA |
| S0299 | 2020-07-27 | Yard or neighborhood | E0476 | NA |
| S0300 | 2020-07-27 | Yard or neighborhood | E0476 | NA |
| S0301 | 2020-07-27 | OTHER | E0483 | Friend/community |
| S0302 | 2020-07-28 | Yard or neighborhood | E0006 | Friend/community |
| S0303 | 2020-07-28 | Yard or neighborhood | E0135 | Newspaper |
| S0304 | 2020-07-28 | Yard or neighborhood | E0195 | Social media |
| S0305 | 2020-07-28 | Yard or neighborhood | E0357 | Social media |
| S0306 | 2020-07-28 | Yard or neighborhood | E0498 | Newspaper |
| S0307 | 2020-07-29 | Yard or neighborhood | E0010 | Newspaper |
| S0308 | 2020-07-29 | Yard or neighborhood | E0056 | NA |
| S0309 | 2020-07-29 | Yard or neighborhood | E0097 | Friend/community |
| S0310 | 2020-07-29 | Yard or neighborhood | E0146 | NA |
| S0311 | 2020-07-29 | Yard or neighborhood | E0266 | Social media |
| S0312 | 2020-07-29 | Yard or neighborhood | NA | NA |
| S0313 | 2020-07-29 | Yard or neighborhood | NA | NA |
| S0314 | 2020-07-30 | Yard or neighborhood | E0112 | NA |
| S0315 | 2020-07-30 | Yard or neighborhood | E0126 | Social media |
| S0316 | 2020-07-30 | Yard or neighborhood | E0126 | Social media |
| S0317 | 2020-07-30 | Yard or neighborhood | E0166 | Internet Search/Amherst College |
| S0318 | 2020-07-30 | Yard or neighborhood | E0234 | NA |
| S0319 | 2020-07-30 | Yard or neighborhood | E0345 | NA |
| S0320 | 2020-07-30 | Yard or neighborhood | NA | Newspaper |
| S0321 | 2020-07-30 | Yard or neighborhood | E0591 | NA |
| S0322 | 2020-07-31 | OTHER | E0116 | NA |
| S0323 | 2020-07-31 | OTHER | E0241 | Social media |
| S0324 | 2020-07-31 | OTHER | E0241 | Social media |
| S0325 | 2020-07-31 | OTHER | E0241 | Social media |
| S0326 | 2020-07-31 | Yard or neighborhood | E0297 | Newspaper |
| S0327 | 2020-07-31 | OTHER | E0402 | Friend/community |
| S0328 | 2020-07-31 | Yard or neighborhood | E0405 | Social media |
| S0329 | 2020-07-31 | Yard or neighborhood | NA | NA |
| S0330 | 2020-07-31 | Forest | NA | NA |
| S0331 | 2020-08-01 | OTHER | E0058 | NA |
| S0332 | 2020-08-01 | OTHER | E0058 | NA |
| S0333 | 2020-08-01 | Yard or neighborhood | E0199 | Friend/community |
| S0334 | 2020-08-01 | Forest | E0307 | Friend/community |
| S0335 | 2020-08-01 | Forest | NA | Social media |
| S0336 | 2020-08-01 | Yard or neighborhood | E0476 | NA |
| S0337 | 2020-08-02 | OTHER | E0016 | Friend/community |
| S0338 | 2020-08-02 | OTHER | E0058 | NA |
| S0339 | 2020-08-02 | OTHER | E0058 | NA |
| S0340 | 2020-08-02 | Yard or neighborhood | E0195 | Social media |
| S0341 | 2020-08-02 | Yard or neighborhood | E0254 | Social media |
| S0342 | 2020-08-02 | Yard or neighborhood | E0254 | Social media |
| S0343 | 2020-08-02 | Yard or neighborhood | E0508 | Social media |
| S0344 | 2020-08-02 | Forest | E0545 | Friend/community |
| S0345 | 2020-08-03 | Yard or neighborhood | E0046 | NA |
| S0346 | 2020-08-03 | OTHER | E0058 | NA |
| S0347 | 2020-08-03 | OTHER | E0058 | NA |
| S0348 | 2020-08-03 | OTHER | E0145 | Internet Search/Amherst College |
| S0349 | 2020-08-03 | Yard or neighborhood | E0175 | Newspaper |
| S0350 | 2020-08-03 | Forest | NA | Social media |
| S0351 | 2020-08-03 | Yard or neighborhood | E0506 | NA |
| S0352 | 2020-08-05 | OTHER | E0058 | NA |
| S0353 | 2020-08-05 | OTHER | E0058 | NA |
| S0354 | 2020-08-05 | Yard or neighborhood | E0304 | Newspaper |
| S0355 | 2020-08-05 | Yard or neighborhood | E0360 | Friend/community |
| S0356 | 2020-08-05 | Yard or neighborhood | E0605 | Newspaper |
| S0357 | 2020-08-06 | Yard or neighborhood | E0052 | Newspaper |
| S0358 | 2020-08-08 | Yard or neighborhood | E0064 | Internet Search/Amherst College |
| S0359 | 2020-08-08 | Yard or neighborhood | E0174 | NA |
| S0360 | 2020-08-09 | OTHER | E0176 | NA |
| S0361 | 2020-08-09 | Yard or neighborhood | E0327 | Social media |
| S0362 | 2020-08-10 | OTHER | E0291 | Friend/community |
| S0363 | 2020-08-11 | Yard or neighborhood | E0157 | Internet Search/Amherst College |
| S0364 | 2020-08-11 | Forest | E0188 | Internet Search/Amherst College |
| S0365 | 2020-08-11 | Yard or neighborhood | E0320 | Newspaper |
| S0366 | 2020-08-12 | Yard or neighborhood | E0089 | Newspaper |
| S0367 | 2020-08-12 | Yard or neighborhood | E0114 | Social media |
| S0368 | 2020-08-12 | Yard or neighborhood | E0195 | Social media |
| S0369 | 2020-08-12 | Yard or neighborhood | E0271 | Social media |
| S0370 | 2020-08-12 | OTHER | NA | Friend/community |
| S0371 | 2020-08-13 | Yard or neighborhood | E0552 | NA |
| S0372 | 2020-08-16 | Yard or neighborhood | E0200 | NA |
| S0373 | 2020-08-16 | Yard or neighborhood | E0291 | NA |
| S0374 | 2020-08-16 | Yard or neighborhood | E0297 | Newspaper |
| S0375 | 2020-08-16 | Yard or neighborhood | E0416 | Friend/community |
| S0376 | 2020-08-16 | Yard or neighborhood | E0606 | Newspaper |
| S0377 | 2020-08-17 | Yard or neighborhood | E0118 | Friend/community |
| S0378 | 2020-08-17 | Yard or neighborhood | E0118 | Friend/community |
| S0379 | 2020-08-18 | OTHER | E0058 | NA |
| S0380 | 2020-08-18 | Yard or neighborhood | E0328 | Friend/community |
| S0381 | 2020-08-18 | Yard or neighborhood | E0509 | Internet Search/Amherst College |
| S0382 | 2020-08-19 | Yard or neighborhood | E0124 | Friend/community |
| S0383 | 2020-08-20 | OTHER | E0058 | NA |
| S0384 | 2020-08-20 | OTHER | E0329 | Internet Search/Amherst College |
| S0385 | 2020-08-20 | Yard or neighborhood | E0422 | Newspaper |
| S0386 | 2020-08-21 | Yard or neighborhood | E0124 | Friend/community |
| S0387 | 2020-08-21 | OTHER | E0590 | Friend/community |
| S0388 | 2020-08-21 | Yard or neighborhood | E0590 | Friend/community |
| S0389 | 2020-08-21 | Yard or neighborhood | E0606 | Newspaper |
| S0390 | 2020-08-22 | Yard or neighborhood | E0355 | Friend/community |
| S0391 | 2020-08-23 | Yard or neighborhood | E0051 | Friend/community |
| S0392 | 2020-08-24 | Yard or neighborhood | E0297 | Newspaper |
| S0393 | 2020-08-24 | Yard or neighborhood | E0605 | Newspaper |
| S0394 | 2020-08-26 | OTHER | E0058 | NA |
| S0395 | 2020-08-26 | OTHER | E0214 | Friend/community |
| S0396 | 2020-08-28 | OTHER | E0058 | NA |
| S0397 | 2020-08-28 | Yard or neighborhood | E0073 | NA |
| S0398 | 2020-08-28 | Forest | E0201 | NA |
| S0399 | 2020-08-28 | Forest | E0281 | NA |
| S0400 | 2020-08-28 | Yard or neighborhood | E0476 | NA |
| S0401 | 2020-08-29 | Yard or neighborhood | E0164 | NA |
| S0402 | 2020-08-29 | Forest | E0349 | Friend/community |
| S0403 | 2020-08-30 | Yard or neighborhood | E0031 | Internet Search/Amherst College |
| S0404 | 2020-08-30 | Yard or neighborhood | E0339 | Internet Search/Amherst College |
| S0405 | 2020-08-31 | Yard or neighborhood | E0012 | Social media |
| S0406 | 2020-08-31 | Yard or neighborhood | E0261 | Internet Search/Amherst College |
| S0407 | 2020-08-31 | Yard or neighborhood | E0261 | Internet Search/Amherst College |
| S0408 | 2020-09-01 | OTHER | E0058 | NA |
| S0409 | 2020-09-01 | Yard or neighborhood | E0124 | Friend/community |
| S0410 | 2020-09-01 | Yard or neighborhood | E0124 | Friend/community |
| S0411 | 2020-09-01 | Yard or neighborhood | E0191 | Friend/community |
| S0412 | 2020-09-02 | OTHER | E0346 | Project Member/Contributor |
| S0413 | 2020-09-03 | OTHER | E0058 | NA |
| S0414 | 2020-09-03 | Yard or neighborhood | E0386 | Newspaper |
| S0415 | 2020-09-04 | OTHER | E0058 | NA |
| S0416 | 2020-09-06 | Forest | E0058 | NA |
| S0417 | 2020-09-08 | Yard or neighborhood | E0142 | Internet Search/Amherst College |
| S0418 | 2020-09-08 | Yard or neighborhood | E0142 | Internet Search/Amherst College |
| S0419 | 2020-09-10 | Yard or neighborhood | E0393 | NA |
| S0420 | 2020-09-10 | Yard or neighborhood | E0399 | Internet Search/Amherst College |
| S0421 | 2020-09-11 | Yard or neighborhood | E0249 | NA |
| S0422 | 2020-09-11 | Yard or neighborhood | E0291 | NA |
| S0423 | 2020-09-14 | OTHER | E0098 | Internet Search/Amherst College |
| S0424 | 2020-09-14 | Yard or neighborhood | E0325 | Internet Search/Amherst College |
| S0425 | 2020-09-16 | Yard or neighborhood | E0290 | Friend/community |
| S0426 | 2020-09-16 | Yard or neighborhood | E0292 | Friend/community |
| S0427 | 2020-09-16 | Yard or neighborhood | NA | Internet Search/Amherst College |
| S0428 | 2020-09-17 | Yard or neighborhood | E0375 | NA |
| S0429 | 2020-09-19 | OTHER | E0096 | NA |
| S0430 | 2020-09-19 | Yard or neighborhood | E0295 | Internet Search/Amherst College |
| S0431 | 2020-09-19 | Yard or neighborhood | E0295 | Internet Search/Amherst College |
| S0432 | 2020-09-20 | Forest | E0058 | NA |
| S0433 | 2020-09-21 | Yard or neighborhood | E0046 | NA |
| S0434 | 2020-09-22 | OTHER | E0289 | Internet Search/Amherst College |
| S0435 | 2020-09-22 | Yard or neighborhood | E0289 | Internet Search/Amherst College |
| S0436 | 2020-09-23 | Yard or neighborhood | E0124 | Friend/community |
| S0437 | 2020-09-23 | Yard or neighborhood | E0247 | Friend/community |
| S0438 | 2020-09-23 | Forest | E0467 | Internet Search/Amherst College |
| S0439 | 2020-09-24 | Yard or neighborhood | E0261 | NA |
| S0440 | 2020-09-24 | Yard or neighborhood | E0492 | Internet Search/Amherst College |
| S0441 | 2020-09-25 | Yard or neighborhood | E0606 | Newspaper |
| S0442 | 2020-09-28 | OTHER | E0263 | Internet Search/Amherst College |
| S0443 | 2020-09-29 | Yard or neighborhood | E0163 | Internet Search/Amherst College |
| S0444 | 2020-09-29 | Yard or neighborhood | E0376 | Internet Search/Amherst College |
| S0445 | 2020-09-29 | Yard or neighborhood | E0376 | Internet Search/Amherst College |
| S0446 | 2020-09-29 | OTHER | E0408 | Project Member/Contributor |
| S0447 | 2020-09-30 | Yard or neighborhood | E0436 | Internet Search/Amherst College |
| S0448 | 2020-09-30 | Yard or neighborhood | E0436 | Newspaper |
| S0449 | 2020-10-01 | Yard or neighborhood | E0206 | NA |
| S0450 | 2020-10-01 | Yard or neighborhood | E0409 | Internet Search/Amherst College |
| S0451 | 2020-10-02 | Yard or neighborhood | E0008 | NA |
| S0452 | 2020-10-02 | Forest | E0035 | Internet Search/Amherst College |
| S0453 | 2020-10-02 | Yard or neighborhood | E0043 | Social media |
| S0454 | 2020-10-02 | Yard or neighborhood | E0139 | Friend/community |
| S0455 | 2020-10-02 | Yard or neighborhood | NA | Social media |
| S0456 | 2020-10-02 | Yard or neighborhood | NA | Social media |
| S0457 | 2020-10-03 | Yard or neighborhood | E0058 | NA |
| S0458 | 2020-10-04 | OTHER | E0116 | Newspaper |
| S0459 | 2020-10-04 | Park | NA | Internet Search/Amherst College |
| S0460 | 2020-10-04 | Park | NA | Internet Search/Amherst College |
| S0461 | 2020-10-04 | Yard or neighborhood | E0612 | NA |
| S0462 | 2020-10-05 | Yard or neighborhood | E0612 | NA |
| S0463 | 2020-10-06 | OTHER | E0058 | NA |
| S0464 | 2020-10-09 | Yard or neighborhood | E0080 | Internet Search/Amherst College |
| S0465 | 2020-10-09 | Yard or neighborhood | E0137 | NA |
| S0466 | 2020-10-09 | Forest | E0226 | Friend/community |
| S0467 | 2020-10-09 | Yard or neighborhood | E0297 | Newspaper |
| S0468 | 2020-10-10 | OTHER | E0116 | Newspaper |
| S0469 | 2020-10-12 | Yard or neighborhood | E0256 | Internet Search/Amherst College |
| S0470 | 2020-10-13 | Yard or neighborhood | E0247 | Friend/community |
| S0471 | 2020-10-13 | Yard or neighborhood | E0247 | Friend/community |
| S0472 | 2020-10-13 | Yard or neighborhood | E0247 | Friend/community |
| S0473 | 2020-10-14 | Yard or neighborhood | E0058 | NA |
| S0474 | 2020-10-14 | Yard or neighborhood | E0269 | Internet Search/Amherst College |
| S0475 | 2020-10-14 | Yard or neighborhood | E0297 | Newspaper |
| S0476 | 2020-10-17 | Yard or neighborhood | E0058 | NA |
| S0477 | 2020-10-17 | Yard or neighborhood | NA | Social media |
| S0478 | 2020-10-18 | Yard or neighborhood | E0005 | NA |
| S0479 | 2020-10-18 | Yard or neighborhood | E0234 | NA |
| S0480 | 2020-10-19 | Yard or neighborhood | E0340 | NA |
| S0481 | 2020-10-21 | Yard or neighborhood | E0116 | Newspaper |
| S0482 | 2020-10-22 | Yard or neighborhood | E0230 | Internet Search/Amherst College |
| S0483 | 2020-10-23 | Yard or neighborhood | E0234 | Friend/community |
| S0484 | 2020-10-23 | Yard or neighborhood | E0234 | Friend/community |
| S0485 | 2020-10-24 | Yard or neighborhood | E0204 | Internet Search/Amherst College |
| S0486 | 2020-10-30 | Yard or neighborhood | E0218 | Internet Search/Amherst College |
| S0487 | 2020-10-30 | Yard or neighborhood | E0218 | Internet Search/Amherst College |
| S0488 | 2020-11-05 | Yard or neighborhood | E0048 | Friend/community |
| S0489 | 2020-11-13 | Yard or neighborhood | E0204 | NA |
| S0490 | 2020-11-19 | Business | NA | NA |
| S0491 | 2020-11-24 | OTHER | E0193 | Newspaper |
| S0492 | 2020-11-27 | Yard or neighborhood | E0297 | Newspaper |
| S0493 | 2020-12-02 | Yard or neighborhood | E0340 | NA |
| S0494 | 2020-12-05 | Yard or neighborhood | E0082 | Internet Search/Amherst College |
| S0495 | 2020-12-06 | Yard or neighborhood | E0536 | Internet Search/Amherst College |
| S0496 | 2020-12-10 | Yard or neighborhood | E0297 | Newspaper |
| S0497 | 2020-12-14 | Forest | E0460 | Internet Search/Amherst College |
| S0498 | 2020-12-14 | Forest | E0460 | Internet Search/Amherst College |
| S0499 | 2020-12-27 | Yard or neighborhood | E0297 | Newspaper |
| S0500 | 2021-01-31 | Yard or neighborhood | E0605 | NA |
| S0501 | 2021-02-25 | Yard or neighborhood | E0308 | Friend/community |
| S0502 | 2021-02-25 | Yard or neighborhood | E0380 | Internet Search/Amherst College |
| S0503 | 2021-02-25 | Yard or neighborhood | E0605 | Newspaper |
| S0504 | 2021-02-26 | Yard or neighborhood | E0308 | Friend/community |
| S0505 | 2021-03-04 | Yard or neighborhood | E0308 | Friend/community |
| S0506 | 2021-03-10 | Yard or neighborhood | E0230 | Internet Search/Amherst College |
| S0507 | 2021-03-11 | Yard or neighborhood | E0605 | Newspaper |
| S0508 | 2021-03-14 | Yard or neighborhood | E0247 | Friend/community |
| S0509 | 2021-03-22 | Yard or neighborhood | E0605 | Newspaper |
| S0510 | 2021-03-23 | Yard or neighborhood | E0608 | NA |
| S0511 | 2021-03-24 | Forest | E0243 | NA |
| S0512 | 2021-03-24 | Yard or neighborhood | E0573 | NA |
| S0513 | 2021-03-25 | Yard or neighborhood | E0022 | Internet Search/Amherst College |
| S0514 | 2021-03-25 | Yard or neighborhood | E0058 | NA |
| S0515 | 2021-03-26 | OTHER | E0058 | Friend/community |
| S0516 | 2021-03-27 | Yard or neighborhood | NA | Internet Search/Amherst College |
| S0517 | 2021-03-29 | Yard or neighborhood | E0512 | Social media |
| S0518 | 2021-04-02 | Yard or neighborhood | E0576 | Internet Search/Amherst College |
| S0519 | 2021-04-03 | Yard or neighborhood | E0227 | Friend/community |
| S0520 | 2021-04-04 | Yard or neighborhood | E0037 | NA |
| S0521 | 2021-04-04 | Yard or neighborhood | E0037 | NA |
| S0522 | 2021-04-04 | OTHER | NA | NA |
| S0523 | 2021-04-04 | Forest | E0530 | Friend/community |
| S0524 | 2021-04-05 | Yard or neighborhood | E0058 | NA |
| S0525 | 2021-04-07 | Yard or neighborhood | E0058 | NA |
| S0526 | 2021-04-09 | Yard or neighborhood | E0001 | Friend/community |
| S0527 | 2021-04-13 | OTHER | E0063 | Internet Search/Amherst College |
| S0528 | 2021-04-13 | OTHER | E0063 | Internet Search/Amherst College |
| S0529 | 2021-04-13 | Yard or neighborhood | E0072 | Internet Search/Amherst College |
| S0530 | 2021-04-13 | Yard or neighborhood | E0072 | Internet Search/Amherst College |
| S0531 | 2021-04-13 | Yard or neighborhood | E0612 | Internet Search/Amherst College |
| S0532 | 2021-04-13 | Yard or neighborhood | E0612 | NA |
| S0533 | 2021-04-14 | Yard or neighborhood | E0578 | NA |
| S0534 | 2021-04-18 | Yard or neighborhood | E0297 | Newspaper |
| S0535 | 2021-04-18 | Yard or neighborhood | NA | NA |
| S0536 | 2021-04-19 | Yard or neighborhood | E0270 | Internet Search/Amherst College |
| S0537 | 2021-04-20 | Yard or neighborhood | E0306 | NA |
| S0538 | 2021-04-20 | Yard or neighborhood | E0386 | NA |
| S0539 | 2021-04-22 | Yard or neighborhood | E0305 | Internet Search/Amherst College |
| S0540 | 2021-04-24 | Yard or neighborhood | E0116 | NA |
| S0541 | 2021-04-24 | Yard or neighborhood | E0116 | NA |
| S0542 | 2021-04-25 | Yard or neighborhood | E0235 | Social media |
| S0543 | 2021-04-25 | Yard or neighborhood | E0258 | Friend/community |
| S0544 | 2021-04-25 | Yard or neighborhood | E0297 | Newspaper |
| S0545 | 2021-04-25 | Yard or neighborhood | E0386 | Newspaper |
| S0546 | 2021-04-25 | Yard or neighborhood | E0386 | Newspaper |
| S0547 | 2021-04-25 | Yard or neighborhood | E0544 | Internet Search/Amherst College |
| S0548 | 2021-04-27 | Yard or neighborhood | E0058 | NA |
| S0549 | 2021-04-27 | Forest | E0323 | Internet Search/Amherst College |
| S0550 | 2021-04-28 | Yard or neighborhood | E0116 | NA |
| S0551 | 2021-04-28 | OTHER | NA | NA |
| S0552 | 2021-05-02 | Yard or neighborhood | E0011 | Friend/community |
| S0553 | 2021-05-02 | Yard or neighborhood | E0197 | Internet Search/Amherst College |
| S0554 | 2021-05-03 | OTHER | NA | NA |
| S0555 | 2021-05-04 | Yard or neighborhood | E0058 | NA |
| S0556 | 2021-05-04 | Yard or neighborhood | E0058 | NA |
| S0557 | 2021-05-04 | OTHER | E0497 | Internet Search/Amherst College |
| S0558 | 2021-05-05 | Yard or neighborhood | E0215 | Social media |
| S0559 | 2021-05-05 | Yard or neighborhood | E0543 | Social media |
| S0560 | 2021-05-06 | Yard or neighborhood | E0151 | NA |
| S0561 | 2021-05-06 | Yard or neighborhood | E0215 | Social media |
| S0562 | 2021-05-06 | Yard or neighborhood | E0366 | Internet Search/Amherst College |
| S0563 | 2021-05-06 | OTHER | E0394 | x |
| S0564 | 2021-05-07 | Yard or neighborhood | E0055 | Internet Search/Amherst College |
| S0565 | 2021-05-07 | Yard or neighborhood | E0124 | Friend/community |
| S0566 | 2021-05-07 | Yard or neighborhood | E0333 | NA |
| S0567 | 2021-05-08 | Yard or neighborhood | E0055 | Internet Search/Amherst College |
| S0568 | 2021-05-08 | Yard or neighborhood | E0272 | Friend/community |
| S0569 | 2021-05-08 | Yard or neighborhood | E0333 | Internet Search/Amherst College |
| S0570 | 2021-05-09 | Yard or neighborhood | E0051 | Friend/community |
| S0571 | 2021-05-10 | Yard or neighborhood | E0033 | Internet Search/Amherst College |
| S0572 | 2021-05-10 | Yard or neighborhood | E0296 | Social media |
| S0573 | 2021-05-10 | Yard or neighborhood | E0485 | Friend/community |
| S0574 | 2021-05-13 | Yard or neighborhood | E0215 | Social media |
| S0575 | 2021-05-13 | Yard or neighborhood | NA | NA |
| S0576 | 2021-05-13 | Yard or neighborhood | E0567 | Internet Search/Amherst College |
| S0577 | 2021-05-13 | Yard or neighborhood | E0613 | Internet Search/Amherst College |
| S0578 | 2021-05-15 | Yard or neighborhood | NA | Social media |
| S0579 | 2021-05-16 | Forest | E0306 | NA |
| S0580 | 2021-05-16 | Yard or neighborhood | NA | NA |
| S0581 | 2021-05-16 | Yard or neighborhood | NA | NA |
| S0582 | 2021-05-16 | Yard or neighborhood | E0608 | NA |
| S0583 | 2021-05-17 | Yard or neighborhood | E0042 | Internet Search/Amherst College |
| S0584 | 2021-05-17 | Yard or neighborhood | E0386 | Newspaper |
| S0585 | 2021-05-18 | Yard or neighborhood | E0055 | NA |
| S0586 | 2021-05-18 | Yard or neighborhood | E0076 | Internet Search/Amherst College |
| S0587 | 2021-05-18 | Yard or neighborhood | NA | Internet Search/Amherst College |
| S0588 | 2021-05-18 | Yard or neighborhood | E0586 | NA |
| S0589 | 2021-05-19 | Yard or neighborhood | E0386 | Newspaper |
| S0590 | 2021-05-19 | Yard or neighborhood | E0604 | Social media |
| S0591 | 2021-05-21 | Yard or neighborhood | NA | Internet Search/Amherst College |
| S0592 | 2021-05-21 | Yard or neighborhood | NA | Internet Search/Amherst College |
| S0593 | 2021-05-22 | OTHER | E0071 | Social media |
| S0594 | 2021-05-22 | Yard or neighborhood | E0105 | NA |
| S0595 | 2021-05-22 | Yard or neighborhood | NA | NA |
| S0596 | 2021-05-23 | Yard or neighborhood | E0359 | Social media |
| S0597 | 2021-05-24 | Yard or neighborhood | E0066 | Internet Search/Amherst College |
| S0598 | 2021-05-24 | Yard or neighborhood | E0066 | Internet Search/Amherst College |
| S0599 | 2021-05-24 | Yard or neighborhood | E0066 | Internet Search/Amherst College |
| S0600 | 2021-05-24 | OTHER | E0284 | Newspaper |
| S0601 | 2021-05-24 | Yard or neighborhood | NA | NA |
| S0602 | 2021-05-24 | Yard or neighborhood | E0474 | Social media |
| S0603 | 2021-05-24 | Yard or neighborhood | E0494 | NA |
| S0604 | 2021-05-25 | Yard or neighborhood | NA | NA |
| S0605 | 2021-05-25 | Yard or neighborhood | E0544 | Internet Search/Amherst College |
| S0606 | 2021-05-26 | Yard or neighborhood | E0070 | Internet Search/Amherst College |
| S0607 | 2021-05-26 | Yard or neighborhood | E0385 | NA |
| S0608 | 2021-05-26 | Street | NA | NA |
| S0609 | 2021-05-26 | Yard or neighborhood | E0499 | Social media |
| S0610 | 2021-05-26 | Yard or neighborhood | E0526 | Social media |
| S0611 | 2021-05-27 | Yard or neighborhood | E0335 | Internet Search/Amherst College |
| S0612 | 2021-05-27 | Yard or neighborhood | NA | NA |
| S0613 | 2021-05-29 | Yard or neighborhood | NA | NA |
| S0614 | 2021-05-29 | Yard or neighborhood | E0524 | Friend/community |
| S0615 | 2021-05-31 | Yard or neighborhood | NA | Internet Search/Amherst College |
| S0616 | 2021-05-31 | Yard or neighborhood | NA | Newspaper |
| S0617 | 2021-06-01 | Yard or neighborhood | E0108 | Internet Search/Amherst College |
| S0618 | 2021-06-01 | Yard or neighborhood | E0120 | NA |
| S0619 | 2021-06-01 | Yard or neighborhood | E0159 | NA |
| S0620 | 2021-06-01 | Yard or neighborhood | E0373 | x |
| S0621 | 2021-06-01 | Yard or neighborhood | E0452 | Social media |
| S0622 | 2021-06-02 | Business | E0220 | Internet Search/Amherst College |
| S0623 | 2021-06-02 | Yard or neighborhood | E0330 | Social media |
| S0624 | 2021-06-02 | Yard or neighborhood | E0331 | Social media |
| S0625 | 2021-06-02 | Yard or neighborhood | E0375 | Internet Search/Amherst College |
| S0626 | 2021-06-02 | Yard or neighborhood | E0395 | Social media |
| S0627 | 2021-06-02 | Yard or neighborhood | E0538 | Social media |
| S0628 | 2021-06-02 | Yard or neighborhood | E0521 | Social media |
| S0629 | 2021-06-03 | Yard or neighborhood | E0184 | Social media |
| S0630 | 2021-06-03 | Yard or neighborhood | E0209 | Social media |
| S0631 | 2021-06-04 | Yard or neighborhood | E0353 | Internet Search/Amherst College |
| S0632 | 2021-06-04 | Forest | E0364 | Social media |
| S0633 | 2021-06-04 | Yard or neighborhood | NA | Social media |
| S0634 | 2021-06-04 | Yard or neighborhood | E0591 | NA |
| S0635 | 2021-06-04 | Yard or neighborhood | E0612 | NA |
| S0636 | 2021-06-05 | Yard or neighborhood | NA | Social media |
| S0637 | 2021-06-05 | Yard or neighborhood | NA | Social media |
| S0638 | 2021-06-05 | Yard or neighborhood | E0471 | Internet Search/Amherst College |
| S0639 | 2021-06-06 | Yard or neighborhood | E0168 | Internet Search/Amherst College |
| S0640 | 2021-06-06 | Forest | E0187 | Social media |
| S0641 | 2021-06-06 | Yard or neighborhood | E0282 | Internet Search/Amherst College |
| S0642 | 2021-06-06 | Forest | NA | NA |
| S0643 | 2021-06-07 | Yard or neighborhood | E0215 | Social media |
| S0644 | 2021-06-07 | Yard or neighborhood | E0310 | Newspaper |
| S0645 | 2021-06-07 | Yard or neighborhood | E0310 | NA |
| S0646 | 2021-06-08 | OTHER | E0015 | Internet Search/Amherst College |
| S0647 | 2021-06-08 | Yard or neighborhood | E0532 | Social media |
| S0648 | 2021-06-09 | Yard or neighborhood | E0167 | Social media |
| S0649 | 2021-06-09 | Yard or neighborhood | E0297 | Newspaper |
| S0650 | 2021-06-09 | Yard or neighborhood | E0386 | Newspaper |
| S0651 | 2021-06-09 | Yard or neighborhood | E0443 | Friend/community |
| S0652 | 2021-06-09 | Yard or neighborhood | NA | NA |
| S0653 | 2021-06-09 | Yard or neighborhood | E0528 | Social media |
| S0654 | 2021-06-10 | Yard or neighborhood | E0007 | Internet Search/Amherst College |
| S0655 | 2021-06-10 | Yard or neighborhood | E0161 | NA |
| S0656 | 2021-06-10 | Yard or neighborhood | E0169 | Social media |
| S0657 | 2021-06-10 | OTHER | E0371 | Internet Search/Amherst College |
| S0658 | 2021-06-10 | Yard or neighborhood | E0473 | Social media |
| S0659 | 2021-06-10 | Yard or neighborhood | E0607 | NA |
| S0660 | 2021-06-11 | Yard or neighborhood | E0044 | Social media |
| S0661 | 2021-06-11 | Yard or neighborhood | E0136 | NA |
| S0662 | 2021-06-11 | Yard or neighborhood | E0451 | Friend/community |
| S0663 | 2021-06-11 | Yard or neighborhood | NA | NA |
| S0664 | 2021-06-11 | Yard or neighborhood | E0462 | x |
| S0665 | 2021-06-11 | Yard or neighborhood | E0601 | Social media |
| S0666 | 2021-06-11 | Yard or neighborhood | E0612 | NA |
| S0667 | 2021-06-12 | Yard or neighborhood | E0454 | Social media |
| S0668 | 2021-06-14 | Yard or neighborhood | E0044 | Social media |
| S0669 | 2021-06-14 | Yard or neighborhood | E0062 | Newspaper |
| S0670 | 2021-06-15 | Yard or neighborhood | E0004 | Internet Search/Amherst College |
| S0671 | 2021-06-15 | Yard or neighborhood | E0259 | Internet Search/Amherst College |
| S0672 | 2021-06-15 | Yard or neighborhood | NA | Internet Search/Amherst College |
| S0673 | 2021-06-15 | Yard or neighborhood | NA | NA |
| S0674 | 2021-06-16 | Yard or neighborhood | E0005 | NA |
| S0675 | 2021-06-16 | Yard or neighborhood | E0351 | NA |
| S0676 | 2021-06-16 | OTHER | E0592 | Social media |
| S0677 | 2021-06-17 | Yard or neighborhood | E0217 | Newspaper |
| S0678 | 2021-06-17 | Forest | E0575 | Newspaper |
| S0679 | 2021-06-18 | Yard or neighborhood | E0312 | Internet Search/Amherst College |
| S0680 | 2021-06-18 | Yard or neighborhood | NA | NA |
| S0681 | 2021-06-18 | Yard or neighborhood | E0527 | Internet Search/Amherst College |
| S0682 | 2021-06-19 | Forest | E0038 | Newspaper |
| S0683 | 2021-06-19 | Yard or neighborhood | E0478 | Internet Search/Amherst College |
| S0684 | 2021-06-20 | Forest | E0228 | Internet Search/Amherst College |
| S0685 | 2021-06-20 | Forest | E0228 | NA |
| S0686 | 2021-06-20 | Forest | E0228 | NA |
| S0687 | 2021-06-20 | Yard or neighborhood | E0231 | Internet Search/Amherst College |
| S0688 | 2021-06-20 | Yard or neighborhood | E0343 | Internet Search/Amherst College |
| S0689 | 2021-06-20 | Yard or neighborhood | E0493 | Social media |
| S0690 | 2021-06-21 | Yard or neighborhood | E0140 | NA |
| S0691 | 2021-06-21 | Yard or neighborhood | E0179 | Social media |
| S0692 | 2021-06-21 | Yard or neighborhood | E0386 | NA |
| S0693 | 2021-06-21 | Yard or neighborhood | E0471 | Internet Search/Amherst College |
| S0694 | 2021-06-21 | OTHER | E0514 | Internet Search/Amherst College |
| S0695 | 2021-06-21 | Forest | E0514 | Internet Search/Amherst College |
| S0696 | 2021-06-21 | Yard or neighborhood | E0576 | Internet Search/Amherst College |
| S0697 | 2021-06-23 | Yard or neighborhood | E0055 | NA |
| S0698 | 2021-06-23 | Yard or neighborhood | E0368 | NA |
| S0699 | 2021-06-24 | Yard or neighborhood | E0021 | NA |
| S0700 | 2021-06-24 | Yard or neighborhood | E0250 | Internet Search/Amherst College |
| S0701 | 2021-06-24 | Yard or neighborhood | E0229 | NA |
| S0702 | 2021-06-25 | Yard or neighborhood | E0217 | Newspaper |
| S0703 | 2021-06-25 | OTHER | E0612 | NA |
| S0704 | 2021-06-26 | OTHER | NA | NA |
| S0705 | 2021-06-27 | Yard or neighborhood | E0237 | Friend/community |
| S0706 | 2021-06-27 | Yard or neighborhood | E0343 | Internet Search/Amherst College |
| S0707 | 2021-06-27 | Yard or neighborhood | E0591 | NA |
| S0708 | 2021-06-28 | OTHER | E0083 | NA |
| S0709 | 2021-06-29 | Forest | E0158 | Internet Search/Amherst College |
| S0710 | 2021-06-29 | Yard or neighborhood | NA | NA |
| S0711 | 2021-06-30 | Yard or neighborhood | NA | Social media |
| S0712 | 2021-06-30 | Business | E0486 | NA |
| S0713 | 2021-07-01 | Yard or neighborhood | E0171 | NA |
| S0714 | 2021-07-01 | Yard or neighborhood | NA | NA |
| S0715 | 2021-07-02 | Yard or neighborhood | E0217 | Newspaper |
| S0716 | 2021-07-02 | Yard or neighborhood | E0286 | Newspaper |
| S0717 | 2021-07-02 | Yard or neighborhood | E0469 | Internet Search/Amherst College |
| S0718 | 2021-07-04 | OTHER | E0034 | Internet Search/Amherst College |
| S0719 | 2021-07-04 | Yard or neighborhood | NA | NA |
| S0720 | 2021-07-04 | Yard or neighborhood | E0566 | Friend/community |
| S0721 | 2021-07-05 | Yard or neighborhood | E0277 | NA |
| S0722 | 2021-07-05 | Yard or neighborhood | E0407 | Social media |
| S0723 | 2021-07-06 | Yard or neighborhood | E0078 | Internet Search/Amherst College |
| S0724 | 2021-07-06 | OTHER | E0198 | Social media |
| S0725 | 2021-07-07 | Yard or neighborhood | E0182 | Internet Search/Amherst College |
| S0726 | 2021-07-07 | Yard or neighborhood | E0182 | Internet Search/Amherst College |
| S0727 | 2021-07-07 | OTHER | E0431 | NA |
| S0728 | 2021-07-08 | Yard or neighborhood | E0268 | Internet Search/Amherst College |
| S0729 | 2021-07-08 | Yard or neighborhood | E0600 | Internet Search/Amherst College |
| S0730 | 2021-07-12 | Yard or neighborhood | E0541 | Friend/community |
| S0731 | 2021-07-13 | Yard or neighborhood | E0587 | Friend/community |
| S0732 | 2021-07-15 | Yard or neighborhood | E0104 | NA |
| S0733 | 2021-07-15 | Forest | NA | Internet Search/Amherst College |
| S0734 | 2021-07-15 | Forest | NA | Internet Search/Amherst College |
| S0735 | 2021-07-16 | Yard or neighborhood | NA | NA |
| S0736 | 2021-07-18 | Yard or neighborhood | E0232 | Internet Search/Amherst College |
| S0737 | 2021-07-18 | OTHER | E0384 | Friend/community |
| S0738 | 2021-07-18 | Yard or neighborhood | E0502 | Internet Search/Amherst College |
| S0739 | 2021-07-21 | Yard or neighborhood | E0115 | Internet Search/Amherst College |
| S0740 | 2021-07-22 | OTHER | E0276 | Friend/community |
| S0741 | 2021-07-23 | Yard or neighborhood | E0532 | Social media |
| S0742 | 2021-07-24 | OTHER | E0084 | Internet Search/Amherst College |
| S0743 | 2021-07-24 | Yard or neighborhood | E0414 | Friend/community |
| S0744 | 2021-07-24 | OTHER | E0293 | Internet Search/Amherst College |
| S0745 | 2021-07-26 | Yard or neighborhood | E0344 | Internet Search/Amherst College |
| S0746 | 2021-07-27 | Yard or neighborhood | NA | Internet Search/Amherst College |
| S0747 | 2021-07-30 | Yard or neighborhood | E0124 | NA |
| S0748 | 2021-07-30 | Yard or neighborhood | E0124 | NA |
| S0749 | 2021-07-30 | Yard or neighborhood | E0124 | NA |
| S0750 | 2021-07-30 | Yard or neighborhood | E0531 | Internet Search/Amherst College |
| S0751 | 2021-08-01 | Yard or neighborhood | NA | Internet Search/Amherst College |
| S0752 | 2021-08-02 | Yard or neighborhood | E0090 | Social media |
| S0753 | 2021-08-03 | Yard or neighborhood | E0324 | NA |
| S0754 | 2021-08-06 | Forest | E0275 | Internet Search/Amherst College |
| S0755 | 2021-08-06 | OTHER | E0362 | Friend/community |
| S0756 | 2021-08-06 | Yard or neighborhood | NA | Internet Search/Amherst College |
| S0757 | 2021-08-07 | Forest | E0027 | Internet Search/Amherst College |
| S0758 | 2021-08-07 | OTHER | E0435 | NA |
| S0759 | 2021-08-07 | Yard or neighborhood | E0550 | Internet Search/Amherst College |
| S0760 | 2021-08-08 | Yard or neighborhood | NA | NA |
| S0761 | 2021-08-08 | Yard or neighborhood | E0233 | Internet Search/Amherst College |
| S0762 | 2021-08-09 | Yard or neighborhood | E0225 | Project Member/Contributor |
| S0763 | 2021-08-09 | Forest | E0228 | NA |
| S0764 | 2021-08-09 | Forest | NA | NA |
| S0765 | 2021-08-10 | Yard or neighborhood | E0309 | Friend/community |
| S0766 | 2021-08-16 | Yard or neighborhood | E0515 | NA |
| S0767 | 2021-08-17 | Yard or neighborhood | E0129 | Internet Search/Amherst College |
| S0768 | 2021-08-18 | Yard or neighborhood | E0572 | Friend/community |
| S0769 | 2021-08-21 | OTHER | E0562 | Internet Search/Amherst College |
| S0770 | 2021-08-24 | Yard or neighborhood | E0587 | Friend/community |
| S0771 | 2021-08-25 | Yard or neighborhood | E0130 | NA |
| S0772 | 2021-08-27 | Yard or neighborhood | E0448 | Internet Search/Amherst College |
| S0773 | 2021-08-27 | Yard or neighborhood | NA | NA |
| S0774 | 2021-08-28 | Yard or neighborhood | E0324 | Internet Search/Amherst College |
| S0775 | 2021-08-30 | Yard or neighborhood | NA | Internet Search/Amherst College |
| S0776 | 2021-09-04 | Yard or neighborhood | E0437 | NA |
| S0777 | 2021-09-04 | Forest | NA | NA |
| S0778 | 2021-09-04 | Yard or neighborhood | E0540 | Friend/community |
| S0779 | 2021-09-06 | Yard or neighborhood | E0606 | NA |
| S0780 | 2021-09-08 | Yard or neighborhood | E0511 | NA |
| S0781 | 2021-09-11 | Yard or neighborhood | E0488 | Internet Search/Amherst College |
| S0782 | 2021-09-11 | Yard or neighborhood | E0488 | NA |
| S0783 | 2021-09-11 | Forest | E0523 | NA |
| S0784 | 2021-09-12 | OTHER | E0459 | NA |
| S0785 | 2021-09-15 | Forest | E0568 | Internet Search/Amherst College |
| S0786 | 2021-09-17 | Yard or neighborhood | E0442 | Internet Search/Amherst College |
| S0787 | 2021-09-21 | Yard or neighborhood | E0433 | Internet Search/Amherst College |
| S0788 | 2021-09-21 | Yard or neighborhood | E0434 | NA |
| S0789 | 2021-09-21 | Yard or neighborhood | E0542 | Social media |
| S0790 | 2021-09-22 | Yard or neighborhood | E0417 | Friend/community |
| S0791 | 2021-09-22 | Yard or neighborhood | E0520 | NA |
| S0792 | 2021-09-25 | Yard or neighborhood | E0448 | Internet Search/Amherst College |
| S0793 | 2021-09-28 | OTHER | E0384 | NA |
| S0794 | 2021-09-28 | OTHER | E0569 | Internet Search/Amherst College |
| S0795 | 2021-09-30 | Yard or neighborhood | E0393 | Newspaper |
| S0796 | 2021-10-01 | Yard or neighborhood | E0609 | Internet Search/Amherst College |
| S0797 | 2021-10-03 | Yard or neighborhood | E0315 | NA |
| S0798 | 2021-10-03 | Yard or neighborhood | E0456 | Internet Search/Amherst College |
| S0799 | 2021-10-04 | Yard or neighborhood | E0061 | NA |
| S0800 | 2021-10-04 | Yard or neighborhood | E0334 | Internet Search/Amherst College |
| S0801 | 2021-10-10 | Forest | E0384 | NA |
| S0802 | 2021-10-12 | Yard or neighborhood | E0049 | Internet Search/Amherst College |
| S0803 | 2021-10-13 | Yard or neighborhood | E0297 | Newspaper |
| S0804 | 2021-10-14 | Yard or neighborhood | E0110 | Social media |
| S0805 | 2021-10-14 | Yard or neighborhood | E0264 | Friend/community |
| S0806 | 2021-10-15 | Yard or neighborhood | E0165 | Friend/community |
| S0807 | 2021-10-15 | Yard or neighborhood | E0389 | Internet Search/Amherst College |
| S0808 | 2021-10-15 | Yard or neighborhood | E0554 | Social media |
| S0809 | 2021-10-16 | Yard or neighborhood | E0207 | Friend/community |
| S0810 | 2021-10-17 | Yard or neighborhood | E0238 | Social media |
| S0811 | 2021-10-18 | Yard or neighborhood | E0222 | Social media |
| S0812 | 2021-10-18 | OTHER | NA | Internet Search/Amherst College |
| S0813 | 2021-10-18 | Forest | NA | Social media |
| S0814 | 2021-10-20 | Yard or neighborhood | E0113 | Social media |
| S0815 | 2021-10-20 | Yard or neighborhood | NA | NA |
| S0816 | 2021-10-21 | Yard or neighborhood | E0165 | Friend/community |
| S0817 | 2021-10-22 | Yard or neighborhood | E0186 | Social media |
| S0818 | 2021-10-23 | Yard or neighborhood | E0411 | NA |
| S0819 | 2021-10-24 | Yard or neighborhood | E0267 | Friend/community |
| S0820 | 2021-10-27 | Forest | NA | NA |
| S0821 | 2021-10-27 | Yard or neighborhood | E0561 | Friend/community |
| S0822 | 2021-10-28 | Yard or neighborhood | NA | Social media |
| S0823 | 2021-10-29 | Yard or neighborhood | E0449 | NA |
| S0824 | 2021-11-01 | Yard or neighborhood | NA | NA |
| S0825 | 2021-11-02 | Yard or neighborhood | E0539 | Internet Search/Amherst College |
| S0826 | 2021-11-03 | Yard or neighborhood | E0297 | Newspaper |
| S0827 | 2021-11-04 | Yard or neighborhood | E0093 | Internet Search/Amherst College |
| S0828 | 2021-11-06 | Yard or neighborhood | NA | NA |
| S0829 | 2021-11-09 | Yard or neighborhood | E0077 | Internet Search/Amherst College |
| S0830 | 2021-11-09 | Forest | NA | NA |
| S0831 | 2021-11-14 | Yard or neighborhood | NA | NA |
| S0832 | 2021-11-15 | Yard or neighborhood | E0297 | Newspaper |
| S0833 | 2021-11-16 | Yard or neighborhood | E0383 | Friend/community |
| S0834 | 2021-11-16 | Yard or neighborhood | NA | NA |
| S0835 | 2021-11-17 | Yard or neighborhood | E0003 | NA |
| S0836 | 2021-11-18 | OTHER | E0030 | Internet Search/Amherst College |
| S0837 | 2021-11-18 | Yard or neighborhood | E0030 | NA |
| S0838 | 2021-11-20 | Forest | E0390 | NA |
| S0839 | 2021-11-21 | Yard or neighborhood | NA | NA |
| S0840 | 2021-11-26 | Yard or neighborhood | E0316 | Internet Search/Amherst College |
| S0841 | 2021-11-26 | Yard or neighborhood | E0316 | Internet Search/Amherst College |
| S0842 | 2021-11-26 | Yard or neighborhood | E0559 | NA |
| S0843 | 2021-11-26 | Yard or neighborhood | E0559 | NA |
| S0844 | 2021-11-26 | Yard or neighborhood | E0560 | x |
| S0845 | 2021-11-26 | Yard or neighborhood | E0560 | x |
| S0846 | 2021-11-29 | Yard or neighborhood | NA | Friend/community |
| S0847 | 2021-11-29 | Yard or neighborhood | NA | Friend/community |
| S0848 | 2021-11-30 | Yard or neighborhood | E0297 | Newspaper |
| S0849 | 2021-11-30 | Yard or neighborhood | E0297 | Newspaper |
| S0850 | 2021-12-08 | Yard or neighborhood | E0058 | NA |
| S0851 | 2021-12-08 | Yard or neighborhood | E0058 | NA |
| S0852 | 2021-12-08 | Yard or neighborhood | E0597 | Internet Search/Amherst College |
| S0853 | 2021-12-08 | Yard or neighborhood | E0597 | Internet Search/Amherst College |
| S0854 | 2021-12-27 | OTHER | E0576 | Internet Search/Amherst College |
| S0855 | 2021-12-27 | OTHER | E0576 | Internet Search/Amherst College |
| S0856 | 2022-01-05 | Yard or neighborhood | E0058 | NA |
| S0857 | 2022-01-05 | Yard or neighborhood | E0058 | NA |
| S0858 | 2022-01-24 | Yard or neighborhood | E0418 | Friend/community |
| S0859 | 2022-01-24 | Yard or neighborhood | E0418 | Friend/community |
| S0860 | 2022-02-01 | Yard or neighborhood | E0057 | Internet Search/Amherst College |
| S0861 | 2022-02-01 | Yard or neighborhood | E0057 | Internet Search/Amherst College |
| S0862 | 2022-02-06 | Yard or neighborhood | E0183 | Internet Search/Amherst College |
| S0863 | 2022-02-06 | Yard or neighborhood | E0183 | Internet Search/Amherst College |
| S0864 | 2022-02-09 | Yard or neighborhood | NA | Internet Search/Amherst College |
| S0865 | 2022-02-09 | Yard or neighborhood | NA | Internet Search/Amherst College |
| S0866 | 2022-02-13 | Yard or neighborhood | NA | NA |
| S0867 | 2022-02-15 | Yard or neighborhood | E0204 | NA |
| S0868 | 2022-02-22 | Yard or neighborhood | E0308 | NA |
| S0869 | 2022-03-05 | Yard or neighborhood | E0150 | NA |
| S0870 | 2022-03-07 | Forest | E0111 | Social media |
| S0871 | 2022-03-14 | Yard or neighborhood | E0203 | Newspaper |
| S0872 | 2022-03-15 | Yard or neighborhood | E0516 | Internet Search/Amherst College |
| S0873 | 2022-03-17 | Yard or neighborhood | E0535 | Newspaper |
| S0874 | 2022-03-19 | Yard or neighborhood | E0107 | Internet Search/Amherst College |
| S0875 | 2022-03-24 | Yard or neighborhood | E0041 | Friend/community |
| S0876 | 2022-03-31 | Forest | E0524 | NA |
| S0877 | 2022-04-02 | Yard or neighborhood | E0382 | Internet Search/Amherst College |
| S0878 | 2022-04-02 | Yard or neighborhood | E0440 | Internet Search/Amherst College |
| S0879 | 2022-04-05 | Yard or neighborhood | E0265 | Newspaper |
| S0880 | 2022-04-08 | Yard or neighborhood | E0519 | NA |
| S0881 | 2022-04-13 | Yard or neighborhood | E0340 | NA |
| S0882 | 2022-04-14 | Yard or neighborhood | E0019 | Newspaper |
| S0883 | 2022-04-19 | Yard or neighborhood | E0513 | NA |
| S0884 | 2022-04-21 | Yard or neighborhood | E0113 | Social media |
| S0885 | 2022-04-21 | Yard or neighborhood | E0465 | NA |
| S0886 | 2022-04-21 | Yard or neighborhood | E0479 | Internet Search/Amherst College |
| S0887 | 2022-04-22 | Yard or neighborhood | NA | NA |
| S0888 | 2022-04-26 | Yard or neighborhood | E0297 | Newspaper |
| S0889 | 2022-04-27 | Forest | NA | NA |
| S0890 | 2022-04-28 | Yard or neighborhood | E0430 | Internet Search/Amherst College |
| S0891 | 2022-04-28 | OTHER | E0577 | NA |
| S0892 | 2022-04-29 | Yard or neighborhood | E0505 | NA |
| S0893 | 2022-05-01 | Yard or neighborhood | E0023 | Internet Search/Amherst College |
| S0894 | 2022-05-01 | Yard or neighborhood | E0208 | Internet Search/Amherst College |
| S0895 | 2022-05-02 | Yard or neighborhood | E0470 | Internet Search/Amherst College |
| S0896 | 2022-05-03 | Yard or neighborhood | E0160 | Newspaper |
| S0897 | 2022-05-03 | Yard or neighborhood | NA | NA |
| S0898 | 2022-05-04 | Yard or neighborhood | E0119 | Friend/community |
| S0899 | 2022-05-04 | Yard or neighborhood | E0598 | Social media |
| S0900 | 2022-05-05 | Yard or neighborhood | E0503 | Friend/community |
| S0901 | 2022-05-06 | Yard or neighborhood | E0260 | Newspaper |
| S0902 | 2022-05-06 | OTHER | E0556 | Internet Search/Amherst College |
| S0903 | 2022-05-07 | Yard or neighborhood | E0517 | Newspaper |
| S0904 | 2022-05-07 | Yard or neighborhood | E0579 | Newspaper |
| S0905 | 2022-05-09 | Yard or neighborhood | E0205 | Social media |
| S0906 | 2022-05-09 | Yard or neighborhood | E0233 | NA |
| S0907 | 2022-05-10 | Yard or neighborhood | E0577 | NA |
| S0908 | 2022-05-11 | Yard or neighborhood | E0180 | NA |
| S0909 | 2022-05-12 | Yard or neighborhood | E0311 | Internet Search/Amherst College |
| S0910 | 2022-05-13 | Yard or neighborhood | E0403 | Internet Search/Amherst College |
| S0911 | 2022-05-14 | Yard or neighborhood | E0194 | Internet Search/Amherst College |
| S0912 | 2022-05-14 | Yard or neighborhood | E0403 | Internet Search/Amherst College |
| S0913 | 2022-05-14 | Yard or neighborhood | E0596 | Social media |
| S0914 | 2022-05-17 | Yard or neighborhood | E0091 | Internet Search/Amherst College |
| S0915 | 2022-05-17 | Yard or neighborhood | E0421 | Internet Search/Amherst College |
| S0916 | 2022-05-18 | Yard or neighborhood | E0588 | Internet Search/Amherst College |
| S0917 | 2022-05-19 | Yard or neighborhood | E0377 | Internet Search/Amherst College |
| S0918 | 2022-05-20 | Yard or neighborhood | E0280 | Friend/community |
| S0919 | 2022-05-20 | OTHER | E0507 | Internet Search/Amherst College |
| S0920 | 2022-05-20 | Forest | E0551 | Internet Search/Amherst College |
| S0921 | 2022-05-22 | Yard or neighborhood | E0351 | NA |
| S0922 | 2022-05-23 | Yard or neighborhood | E0180 | Internet Search/Amherst College |
| S0923 | 2022-05-24 | Yard or neighborhood | E0185 | Newspaper |
| S0924 | 2022-05-24 | Yard or neighborhood | E0585 | Internet Search/Amherst College |
| S0925 | 2022-05-25 | Yard or neighborhood | E0297 | Newspaper |
| S0926 | 2022-05-26 | Yard or neighborhood | E0164 | x |
| S0927 | 2022-05-26 | Yard or neighborhood | E0475 | Internet Search/Amherst College |
| S0928 | 2022-05-27 | Yard or neighborhood | E0489 | Internet Search/Amherst College |
| S0929 | 2022-05-30 | Yard or neighborhood | E0124 | NA |
| S0930 | 2022-05-31 | Yard or neighborhood | E0397 | Internet Search/Amherst College |
| S0931 | 2022-06-01 | Yard or neighborhood | E0040 | Internet Search/Amherst College |
| S0932 | 2022-06-02 | OTHER | E0228 | NA |
| S0933 | 2022-06-03 | Yard or neighborhood | E0388 | Newspaper |
| S0934 | 2022-06-06 | Yard or neighborhood | E0068 | Internet Search/Amherst College |
| S0935 | 2022-06-06 | Yard or neighborhood | E0152 | Friend/community |
| S0936 | 2022-06-07 | OTHER | E0482 | Friend/community |
| S0937 | 2022-06-08 | Business | E0348 | NA |
| S0938 | 2022-06-08 | Yard or neighborhood | E0363 | Internet Search/Amherst College |
| S0939 | 2022-06-10 | Yard or neighborhood | E0104 | NA |
| S0940 | 2022-06-14 | Yard or neighborhood | E0203 | NA |
| S0941 | 2022-06-15 | Yard or neighborhood | E0279 | Newspaper |
| S0942 | 2022-06-16 | Yard or neighborhood | E0101 | Friend/community |
| S0943 | 2022-06-16 | Yard or neighborhood | NA | NA |
| S0944 | 2022-06-17 | Yard or neighborhood | E0101 | Friend/community |
| S0945 | 2022-06-17 | Yard or neighborhood | E0196 | Internet Search/Amherst College |
| S0946 | 2022-06-17 | Yard or neighborhood | E0363 | Internet Search/Amherst College |
| S0947 | 2022-06-17 | Yard or neighborhood | E0388 | NA |
| S0948 | 2022-06-17 | Yard or neighborhood | E0585 | Internet Search/Amherst College |
| S0949 | 2022-06-19 | Yard or neighborhood | E0297 | Newspaper |
| S0950 | 2022-06-19 | OTHER | E0358 | Internet Search/Amherst College |
| S0951 | 2022-06-19 | Yard or neighborhood | NA | NA |
| S0952 | 2022-06-19 | Yard or neighborhood | E0580 | Internet Search/Amherst College |
| S0953 | 2022-06-20 | Yard or neighborhood | E0162 | Internet Search/Amherst College |
| S0954 | 2022-06-20 | Yard or neighborhood | E0314 | Internet Search/Amherst College |
| S0955 | 2022-06-20 | Yard or neighborhood | E0427 | Internet Search/Amherst College |
| S0956 | 2022-06-21 | Yard or neighborhood | E0125 | Internet Search/Amherst College |
| S0957 | 2022-06-21 | Forest | NA | NA |
| S0958 | 2022-06-24 | Yard or neighborhood | E0297 | Newspaper |
| S0959 | 2022-06-24 | Forest | E0472 | Internet Search/Amherst College |
| S0960 | 2022-06-26 | Yard or neighborhood | E0288 | Internet Search/Amherst College |
| S0961 | 2022-06-26 | Yard or neighborhood | NA | NA |
| S0962 | 2022-06-27 | Yard or neighborhood | E0369 | Internet Search/Amherst College |
| S0963 | 2022-06-30 | Yard or neighborhood | E0252 | NA |
| S0964 | 2022-07-02 | Yard or neighborhood | E0388 | NA |
| S0965 | 2022-07-04 | Yard or neighborhood | E0194 | Internet Search/Amherst College |
| S0966 | 2022-07-06 | Yard or neighborhood | E0381 | Internet Search/Amherst College |
| S0967 | 2022-07-06 | Yard or neighborhood | E0581 | Newspaper |
| S0968 | 2022-07-07 | Yard or neighborhood | E0202 | Internet Search/Amherst College |
| S0969 | 2022-07-07 | OTHER | E0228 | NA |
| S0970 | 2022-07-08 | OTHER | E0228 | NA |
| S0971 | 2022-07-09 | Forest | E0086 | Internet Search/Amherst College |
| S0972 | 2022-07-09 | Yard or neighborhood | NA | Internet Search/Amherst College |
| S0973 | 2022-07-09 | Yard or neighborhood | E0458 | Internet Search/Amherst College |
| S0974 | 2022-07-10 | Yard or neighborhood | E0228 | NA |
| S0975 | 2022-07-10 | Yard or neighborhood | NA | NA |
| S0976 | 2022-07-11 | OTHER | E0319 | Internet Search/Amherst College |
| S0977 | 2022-07-12 | Yard or neighborhood | E0582 | Internet Search/Amherst College |
| S0978 | 2022-07-13 | Forest | E0299 | NA |
| S0979 | 2022-07-15 | Forest | E0582 | Internet Search/Amherst College |
| S0980 | 2022-07-17 | OTHER | E0228 | NA |
| S0981 | 2022-07-19 | OTHER | E0060 | Internet Search/Amherst College |
| S0982 | 2022-07-20 | Yard or neighborhood | E0495 | Internet Search/Amherst College |
| S0983 | 2022-07-23 | OTHER | E0228 | NA |
| S0984 | 2022-07-24 | Yard or neighborhood | E0577 | NA |
| S0985 | 2022-07-26 | Yard or neighborhood | E0453 | NA |
| S0986 | 2022-07-28 | Yard or neighborhood | E0153 | Internet Search/Amherst College |
| S0987 | 2022-07-29 | Yard or neighborhood | E0594 | NA |
| S0988 | 2022-07-31 | Yard or neighborhood | E0300 | Internet Search/Amherst College |
| S0989 | 2022-07-31 | Yard or neighborhood | E0400 | Internet Search/Amherst College |
| S0990 | 2022-08-03 | Forest | E0529 | Internet Search/Amherst College |
| S0991 | 2022-08-06 | Yard or neighborhood | E0081 | x |
| S0992 | 2022-08-10 | Yard or neighborhood | E0322 | Internet Search/Amherst College |
| S0993 | 2022-08-12 | Yard or neighborhood | E0079 | Internet Search/Amherst College |
| S0994 | 2022-08-19 | OTHER | E0412 | NA |
| S0995 | 2022-08-22 | Yard or neighborhood | E0176 | NA |
| S0996 | 2022-08-24 | Yard or neighborhood | E0480 | Internet Search/Amherst College |
| S0997 | 2022-08-25 | Yard or neighborhood | E0480 | NA |
| S0998 | 2022-08-27 | Yard or neighborhood | E0508 | NA |
| S0999 | 2022-08-28 | Yard or neighborhood | E0589 | NA |
| S1000 | 2022-08-30 | Yard or neighborhood | E0036 | NA |
| S1001 | 2022-08-30 | Yard or neighborhood | E0045 | Newspaper |
| S1002 | 2022-08-31 | Forest | E0219 | Newspaper |
| S1003 | 2022-08-31 | Yard or neighborhood | E0444 | NA |
| S1004 | 2022-09-01 | Yard or neighborhood | E0099 | NA |
| S1005 | 2022-09-03 | Yard or neighborhood | E0099 | Newspaper |
| S1006 | 2022-09-03 | Yard or neighborhood | E0173 | NA |
| S1007 | 2022-09-03 | Yard or neighborhood | E0332 | NA |
| S1008 | 2022-09-04 | Yard or neighborhood | E0094 | NA |
| S1009 | 2022-09-04 | Yard or neighborhood | E0255 | NA |
| S1010 | 2022-09-05 | Forest | E0565 | NA |
| S1011 | 2022-09-06 | Yard or neighborhood | E0002 | NA |
| S1012 | 2022-09-06 | Yard or neighborhood | E0350 | NA |
| S1013 | 2022-09-07 | Yard or neighborhood | E0138 | Internet Search/Amherst College |
| S1014 | 2022-09-08 | Yard or neighborhood | E0438 | NA |
| S1015 | 2022-09-08 | Yard or neighborhood | E0477 | NA |
| S1016 | 2022-09-09 | Yard or neighborhood | E0176 | NA |
| S1017 | 2022-09-09 | Yard or neighborhood | E0504 | NA |
| S1018 | 2022-09-09 | Yard or neighborhood | E0595 | NA |
| S1019 | 2022-09-10 | Yard or neighborhood | E0087 | NA |
| S1020 | 2022-09-11 | Yard or neighborhood | E0487 | NA |
| S1021 | 2022-09-13 | Yard or neighborhood | E0342 | Internet Search/Amherst College |
| S1022 | 2022-09-15 | Yard or neighborhood | E0441 | NA |
| S1023 | 2022-09-18 | Forest | E0396 | Newspaper |
| S1024 | 2022-09-22 | OTHER | E0100 | NA |
| S1025 | 2022-09-29 | Yard or neighborhood | E0426 | NA |
| S1026 | 2022-10-04 | Yard or neighborhood | E0426 | NA |

**Date of bear sightings and corresponding percent forest cover (MassBears)**

| **date** | **forestsValue** |
| --- | --- |
| 4/1/2019 | 0.53 |
| 4/8/2019 | 0.925 |
| 4/8/2019 | 0.925 |
| 4/25/2019 | 0.336667 |
| 5/18/2019 | 0.53 |
| 6/9/2019 | 0.79 |
| 6/12/2019 | 0.82 |
| 6/12/2019 | 0.89 |
| 6/12/2019 | 0.73 |
| 6/13/2019 | 0.87 |
| 6/14/2019 | 0.8 |
| 6/14/2019 | 0.766667 |
| 6/14/2019 | 0.775 |
| 6/19/2019 | 0.955 |
| 6/22/2019 | 0.675 |
| 7/23/2019 | 0.286667 |
| 7/25/2019 | 0.005 |
| 7/25/2019 | 0.9 |
| 7/26/2019 | 0.92 |
| 8/6/2019 | 0.87 |
| 8/7/2019 | 0.7 |
| 8/14/2019 | 0.39 |
| 10/1/2019 | 0.575 |
| 4/12/2020 | 0.03 |
| 4/24/2020 | 0.01 |
| 4/25/2020 | 0.325 |
| 4/25/2020 | 0.016667 |
| 4/26/2020 | 0.3 |
| 4/28/2020 | 0.89 |
| 4/28/2020 | 0.89 |
| 5/2/2020 | 0.925 |
| 5/2/2020 | 0.87 |
| 5/3/2020 | 0.71 |
| 5/3/2020 | 0.63 |
| 5/3/2020 | 0.63 |
| 5/4/2020 | 0.58 |
| 5/6/2020 | 0.65 |
| 5/6/2020 | 0.5025 |
| 5/6/2020 | 0.35 |
| 5/9/2020 | 0.78 |
| 5/10/2020 | 0.56 |
| 5/11/2020 | 0.35 |
| 5/12/2020 | 0.22 |
| 5/13/2020 | 0.826667 |
| 5/14/2020 | 0.565 |
| 5/15/2020 | 0.41 |
| 5/15/2020 | 0.21 |
| 5/15/2020 | 0.92 |
| 5/15/2020 | 0.856667 |
| 5/16/2020 | 0.275 |
| 5/17/2020 | 0.943333 |
| 5/17/2020 | 0.83 |
| 5/17/2020 | 0.885 |
| 5/17/2020 | 0.885 |
| 5/18/2020 | 0.895 |
| 5/18/2020 | 0.81 |
| 5/19/2020 | 0.41 |
| 5/19/2020 | 0.88 |
| 5/21/2020 | 0.105 |
| 5/21/2020 | 0.41 |
| 5/21/2020 | 0.88 |
| 5/22/2020 | 0.41 |
| 5/22/2020 | 0.66 |
| 5/22/2020 | 0.855 |
| 5/23/2020 | 0.235 |
| 5/23/2020 | 0.855 |
| 5/24/2020 | 0.6 |
| 5/24/2020 | 0.92 |
| 5/24/2020 | 0.61 |
| 5/25/2020 | 0.765 |
| 5/25/2020 | 0.935 |
| 5/26/2020 | 0.01 |
| 5/26/2020 | 0.89 |
| 5/26/2020 | 0.67 |
| 5/26/2020 | 0.965 |
| 5/27/2020 | 0.105 |
| 5/27/2020 | 0.64 |
| 5/28/2020 | 0.335 |
| 5/31/2020 | 0.41 |
| 5/31/2020 | 0.345 |
| 6/1/2020 | 0.41 |
| 6/1/2020 | 0.08 |
| 6/2/2020 | 0.2 |
| 6/2/2020 | 0.105 |
| 6/5/2020 | 0.785 |
| 6/5/2020 | 0.785 |
| 6/6/2020 | 0.11 |
| 6/7/2020 | 0.785 |
| 6/8/2020 | 0.08 |
| 6/8/2020 | 0.08 |
| 6/8/2020 | 0.355 |
| 6/8/2020 | 0.75 |
| 6/8/2020 | 0.69 |
| 6/9/2020 | 0.375 |
| 6/9/2020 | 0.235 |
| 6/9/2020 | 0.69 |
| 6/9/2020 | 0.64 |
| 6/10/2020 | 0.175 |
| 6/10/2020 | 0.02 |
| 6/10/2020 | 0.88 |
| 6/10/2020 | 0 |
| 6/12/2020 | 0.08 |
| 6/12/2020 | 0.08 |
| 6/13/2020 | 0.27 |
| 6/13/2020 | 0.685 |
| 6/13/2020 | 0.785 |
| 6/14/2020 | 0.41 |
| 6/14/2020 | 0.38 |
| 6/15/2020 | 0.54 |
| 6/15/2020 | 0.54 |
| 6/15/2020 | 0.69 |
| 6/15/2020 | 0.56 |
| 6/15/2020 | 0.765 |
| 6/17/2020 | 0.13 |
| 6/17/2020 | 0.13 |
| 6/17/2020 | 0.365 |
| 6/18/2020 | 0.41 |
| 6/18/2020 | 0 |
| 6/18/2020 | 0.785 |
| 6/18/2020 | 0.32 |
| 6/19/2020 | 0.53 |
| 6/19/2020 | 0.395 |
| 6/20/2020 | 0.67 |
| 6/20/2020 | 0.77 |
| 6/21/2020 | 0.67 |
| 6/21/2020 | 0.68 |
| 6/21/2020 | 0.903333 |
| 6/22/2020 | 0.08 |
| 6/22/2020 | 0.595 |
| 6/23/2020 | 0.56 |
| 6/23/2020 | 0.483333 |
| 6/23/2020 | 0.525 |
| 6/24/2020 | 0.235 |
| 6/24/2020 | 0.05 |
| 6/24/2020 | 0.16 |
| 6/24/2020 | 0.03 |
| 6/24/2020 | 0.785 |
| 6/25/2020 | 0.04 |
| 6/25/2020 | 0.776667 |
| 6/25/2020 | 0.27 |
| 6/26/2020 | 0.25 |
| 6/26/2020 | 0.015 |
| 6/26/2020 | 0.355 |
| 6/26/2020 | 0.79 |
| 6/26/2020 | 0.8 |
| 6/26/2020 | 0.93 |
| 6/27/2020 | 0.186667 |
| 6/27/2020 | 0.57 |
| 6/27/2020 | 0.555 |
| 6/28/2020 | 0.68 |
| 6/28/2020 | 0.785 |
| 6/29/2020 | 0.6 |
| 6/30/2020 | 0.035 |
| 6/30/2020 | 0.61 |
| 6/30/2020 | 0.685 |
| 7/1/2020 | 0.69 |
| 7/1/2020 | 0.34 |
| 7/1/2020 | 0.34 |
| 7/1/2020 | 0.33 |
| 7/1/2020 | 0.24 |
| 7/1/2020 | 0.255 |
| 7/2/2020 | 0.59 |
| 7/2/2020 | 0.11 |
| 7/2/2020 | 0.1025 |
| 7/2/2020 | 0.69 |
| 7/3/2020 | 0.17 |
| 7/3/2020 | 0.19 |
| 7/3/2020 | 0.355 |
| 7/3/2020 | 0.895 |
| 7/4/2020 | 0.59 |
| 7/4/2020 | 0.36 |
| 7/4/2020 | 0.665 |
| 7/5/2020 | 0.2 |
| 7/5/2020 | 0.33 |
| 7/5/2020 | 0.59 |
| 7/5/2020 | 0.255 |
| 7/6/2020 | 0.303333 |
| 7/6/2020 | 0.585 |
| 7/6/2020 | 0.64 |
| 7/7/2020 | 0.05 |
| 7/7/2020 | 0.2 |
| 7/7/2020 | 0.37 |
| 7/7/2020 | 0.16 |
| 7/7/2020 | 0.27 |
| 7/8/2020 | 0.2 |
| 7/8/2020 | 0.065 |
| 7/8/2020 | 0.6125 |
| 7/8/2020 | 0.12 |
| 7/8/2020 | 0.27 |
| 7/8/2020 | 0.59 |
| 7/8/2020 | 0.41 |
| 7/9/2020 | 0.69 |
| 7/9/2020 | 0.64 |
| 7/9/2020 | 0.665 |
| 7/9/2020 | 0.213333 |
| 7/9/2020 | 0.335 |
| 7/10/2020 | 0.67 |
| 7/10/2020 | 0.67 |
| 7/10/2020 | 0.065 |
| 7/10/2020 | 0.08 |
| 7/10/2020 | 0.22 |
| 7/10/2020 | 0.42 |
| 7/10/2020 | 0.72 |
| 7/10/2020 | 0.38 |
| 7/11/2020 | 0.07 |
| 7/11/2020 | 0.065 |
| 7/11/2020 | 0.81 |
| 7/12/2020 | 0.51 |
| 7/12/2020 | 0.47 |
| 7/12/2020 | 0.56 |
| 7/12/2020 | 0.32 |
| 7/12/2020 | 0.63 |
| 7/13/2020 | 0.46 |
| 7/13/2020 | 0.35 |
| 7/13/2020 | 0.07 |
| 7/13/2020 | 0.29 |
| 7/13/2020 | 0.43 |
| 7/14/2020 | 0.84 |
| 7/14/2020 | 0.38 |
| 7/14/2020 | 0.11 |
| 7/15/2020 | 0 |
| 7/15/2020 | 0.52 |
| 7/15/2020 | 0.845 |
| 7/15/2020 | 0.11 |
| 7/15/2020 | 0.235 |
| 7/16/2020 | 0.63 |
| 7/16/2020 | 0.65 |
| 7/16/2020 | 0.87 |
| 7/17/2020 | 0.915 |
| 7/17/2020 | 0.915 |
| 7/17/2020 | 0.56 |
| 7/17/2020 | 0.525 |
| 7/17/2020 | 0.52 |
| 7/17/2020 | 0.465 |
| 7/17/2020 | 0.035 |
| 7/17/2020 | 0.59 |
| 7/17/2020 | 0.23 |
| 7/18/2020 | 0.82 |
| 7/18/2020 | 0.16 |
| 7/18/2020 | 0.665 |
| 7/18/2020 | 0.15 |
| 7/18/2020 | 0.15 |
| 7/18/2020 | 0.62 |
| 7/19/2020 | 0.36 |
| 7/19/2020 | 0.19 |
| 7/19/2020 | 0.29 |
| 7/19/2020 | 0.83 |
| 7/19/2020 | 0.74 |
| 7/19/2020 | 0.885 |
| 7/20/2020 | 0.15 |
| 7/20/2020 | 0.375 |
| 7/20/2020 | 0.83 |
| 7/20/2020 | 0.785 |
| 7/20/2020 | 0.675 |
| 7/21/2020 | 0.305 |
| 7/21/2020 | 0.24 |
| 7/21/2020 | 0.44 |
| 7/21/2020 | 0 |
| 7/21/2020 | 0.59 |
| 7/21/2020 | 0.49 |
| 7/22/2020 | 0.566667 |
| 7/22/2020 | 0.303333 |
| 7/22/2020 | 0.536667 |
| 7/22/2020 | 0.19 |
| 7/22/2020 | 0.19 |
| 7/22/2020 | 0.19 |
| 7/22/2020 | 0.12 |
| 7/22/2020 | 0.785 |
| 7/23/2020 | 0.09 |
| 7/23/2020 | 0.35 |
| 7/23/2020 | 0.05 |
| 7/23/2020 | 0.18 |
| 7/23/2020 | 0.785 |
| 7/24/2020 | 0.675 |
| 7/24/2020 | 0.82 |
| 7/25/2020 | 0.51 |
| 7/25/2020 | 0.24 |
| 7/25/2020 | 0.175 |
| 7/25/2020 | 0.06 |
| 7/26/2020 | 0.696667 |
| 7/26/2020 | 0.696667 |
| 7/26/2020 | 0.696667 |
| 7/26/2020 | 0.915 |
| 7/26/2020 | 0.915 |
| 7/26/2020 | 0.915 |
| 7/26/2020 | 0.89 |
| 7/26/2020 | 0.645 |
| 7/26/2020 | 0.375 |
| 7/26/2020 | 0.93 |
| 7/27/2020 | 0.39 |
| 7/27/2020 | 0.305 |
| 7/27/2020 | 0.98 |
| 7/27/2020 | 0.52 |
| 7/27/2020 | 0.52 |
| 7/27/2020 | 0.555 |
| 7/27/2020 | 0.515 |
| 7/27/2020 | 0.04 |
| 7/27/2020 | 0.14 |
| 7/28/2020 | 0.743333 |
| 7/28/2020 | 0.615 |
| 7/28/2020 | 0.81 |
| 7/28/2020 | 0.93 |
| 7/28/2020 | 0.33 |
| 7/29/2020 | 0.59 |
| 7/29/2020 | 0.335 |
| 7/29/2020 | 0.2525 |
| 7/29/2020 | 0.2525 |
| 7/29/2020 | 0.125 |
| 7/29/2020 | 0.42 |
| 7/29/2020 | 0.24 |
| 7/30/2020 | 0.73 |
| 7/30/2020 | 0.56 |
| 7/30/2020 | 0.98 |
| 7/30/2020 | 0.635 |
| 7/30/2020 | 0.043333 |
| 7/30/2020 | 0.84 |
| 7/30/2020 | 0 |
| 7/30/2020 | 0 |
| 7/31/2020 | 0.305 |
| 7/31/2020 | 0.95 |
| 7/31/2020 | 0.5 |
| 7/31/2020 | 0.5 |
| 7/31/2020 | 0.5 |
| 7/31/2020 | 0.61 |
| 7/31/2020 | 0.7 |
| 7/31/2020 | 0.115 |
| 7/31/2020 | 0.585 |
| 8/1/2020 | 0.69 |
| 8/1/2020 | 0.52 |
| 8/1/2020 | 0.64 |
| 8/1/2020 | 0.85 |
| 8/1/2020 | 0.785 |
| 8/1/2020 | 0.785 |
| 8/2/2020 | 0.6 |
| 8/2/2020 | 0.645 |
| 8/2/2020 | 0.645 |
| 8/2/2020 | 0.645 |
| 8/2/2020 | 0.93 |
| 8/2/2020 | 0.835 |
| 8/2/2020 | 0.785 |
| 8/2/2020 | 0.785 |
| 8/3/2020 | 0.51 |
| 8/3/2020 | 0.275 |
| 8/3/2020 | 0.71 |
| 8/3/2020 | 0.59 |
| 8/3/2020 | 0.585 |
| 8/3/2020 | 0.785 |
| 8/3/2020 | 0.785 |
| 8/5/2020 | 0.24 |
| 8/5/2020 | 0.14 |
| 8/5/2020 | 0.315 |
| 8/5/2020 | 0.785 |
| 8/5/2020 | 0.785 |
| 8/6/2020 | 0.23 |
| 8/8/2020 | 0.415 |
| 8/8/2020 | 0.71 |
| 8/9/2020 | 0.01 |
| 8/9/2020 | 0.525 |
| 8/10/2020 | 0.83 |
| 8/11/2020 | 0.07 |
| 8/11/2020 | 0.766667 |
| 8/12/2020 | 0.565 |
| 8/12/2020 | 0.125 |
| 8/12/2020 | 0.33 |
| 8/12/2020 | 0.93 |
| 8/12/2020 | 0.75 |
| 8/13/2020 | 0.55 |
| 8/16/2020 | 0.305 |
| 8/16/2020 | 0.69 |
| 8/16/2020 | 0.28 |
| 8/16/2020 | 0.08 |
| 8/16/2020 | 0.885 |
| 8/17/2020 | 0.605 |
| 8/17/2020 | 0.605 |
| 8/18/2020 | 0.695 |
| 8/18/2020 | 0.636667 |
| 8/18/2020 | 0.785 |
| 8/19/2020 | 0.21 |
| 8/20/2020 | 0.305 |
| 8/20/2020 | 0.14 |
| 8/20/2020 | 0.785 |
| 8/21/2020 | 0.21 |
| 8/21/2020 | 0.08 |
| 8/21/2020 | 0.245 |
| 8/21/2020 | 0.245 |
| 8/22/2020 | 0.77 |
| 8/23/2020 | 0.42 |
| 8/24/2020 | 0.305 |
| 8/24/2020 | 0.715 |
| 8/26/2020 | 0.53 |
| 8/26/2020 | 0.916667 |
| 8/28/2020 | 0.325 |
| 8/28/2020 | 0.07 |
| 8/28/2020 | 0.52 |
| 8/28/2020 | 0.935 |
| 8/28/2020 | 0.785 |
| 8/29/2020 | 0.443333 |
| 8/30/2020 | 0.06 |
| 8/31/2020 | 0.485 |
| 8/31/2020 | 0.485 |
| 8/31/2020 | 0.18 |
| 9/1/2020 | 0.37 |
| 9/1/2020 | 0.21 |
| 9/1/2020 | 0.21 |
| 9/1/2020 | 0.785 |
| 9/2/2020 | 0.02 |
| 9/3/2020 | 0.303333 |
| 9/3/2020 | 0.785 |
| 9/4/2020 | 0.785 |
| 9/6/2020 | 0.85 |
| 9/8/2020 | 0.48 |
| 9/8/2020 | 0.48 |
| 9/10/2020 | 0.39 |
| 9/10/2020 | 0.47 |
| 9/11/2020 | 0.69 |
| 9/11/2020 | 0.69 |
| 9/14/2020 | 0.305 |
| 9/16/2020 | 0.69 |
| 9/16/2020 | 0.69 |
| 9/16/2020 | 0.585 |
| 9/17/2020 | 0.655 |
| 9/19/2020 | 0.426667 |
| 9/19/2020 | 0.426667 |
| 9/20/2020 | 0.85 |
| 9/21/2020 | 0.59 |
| 9/22/2020 | 0.835 |
| 9/22/2020 | 0.835 |
| 9/23/2020 | 0.69 |
| 9/23/2020 | 0.21 |
| 9/24/2020 | 0.185 |
| 9/24/2020 | 0.485 |
| 9/25/2020 | 0 |
| 9/28/2020 | 0 |
| 9/29/2020 | 0.685 |
| 9/29/2020 | 0.63 |
| 9/29/2020 | 0.63 |
| 9/29/2020 | 0.395 |
| 9/30/2020 | 0.135 |
| 9/30/2020 | 0.135 |
| 10/1/2020 | 0.19 |
| 10/1/2020 | 0.03 |
| 10/2/2020 | 0.005 |
| 10/2/2020 | 0.12 |
| 10/2/2020 | 0.19 |
| 10/2/2020 | 0.19 |
| 10/2/2020 | 0.1275 |
| 10/2/2020 | 0.965 |
| 10/3/2020 | 0.785 |
| 10/4/2020 | 0.225 |
| 10/4/2020 | 0.006667 |
| 10/4/2020 | 0.006667 |
| 10/4/2020 | 0.72 |
| 10/5/2020 | 0.225 |
| 10/6/2020 | 0.785 |
| 10/9/2020 | 0.305 |
| 10/9/2020 | 0 |
| 10/9/2020 | 0.575 |
| 10/9/2020 | 0.253333 |
| 10/10/2020 | 0.72 |
| 10/12/2020 | 0.105 |
| 10/13/2020 | 0.69 |
| 10/13/2020 | 0.69 |
| 10/13/2020 | 0.69 |
| 10/14/2020 | 0.305 |
| 10/14/2020 | 0.205 |
| 10/14/2020 | 0.785 |
| 10/17/2020 | 0 |
| 10/17/2020 | 0.785 |
| 10/18/2020 | 0.925 |
| 10/18/2020 | 0.285 |
| 10/19/2020 | 0.06 |
| 10/21/2020 | 0.72 |
| 10/22/2020 | 0.15 |
| 10/23/2020 | 0.66 |
| 10/23/2020 | 0.66 |
| 10/24/2020 | 0.14 |
| 10/30/2020 | 0.54 |
| 10/30/2020 | 0.54 |
| 11/5/2020 | 0.27 |
| 11/13/2020 | 0.3 |
| 11/19/2020 | 0.255 |
| 11/24/2020 | 0.83 |
| 11/27/2020 | 0.305 |
| 12/2/2020 | 0.06 |
| 12/5/2020 | 0.28 |
| 12/6/2020 | 0.805 |
| 12/10/2020 | 0.305 |
| 12/14/2020 | 0.575 |
| 12/14/2020 | 0.575 |
| 12/27/2020 | 0.305 |
| 1/31/2021 | 0.715 |
| 2/25/2021 | 0.23 |
| 2/25/2021 | 0.43 |
| 2/25/2021 | 0.715 |
| 2/26/2021 | 0.23 |
| 3/4/2021 | 0.23 |
| 3/10/2021 | 0.81 |
| 3/11/2021 | 0.715 |
| 3/14/2021 | 0.69 |
| 3/22/2021 | 0.715 |
| 3/23/2021 | 0.03 |
| 3/24/2021 | 0.36 |
| 3/24/2021 | 0.34 |
| 3/25/2021 | 0.47 |
| 3/25/2021 | 0.785 |
| 3/26/2021 | 0.93 |
| 3/27/2021 | 0.476667 |
| 3/29/2021 | 0.596667 |
| 4/2/2021 | 0.413333 |
| 4/3/2021 | 0.21 |
| 4/4/2021 | 0.915 |
| 4/4/2021 | 0.915 |
| 4/4/2021 | 0 |
| 4/4/2021 | 0.24 |
| 4/5/2021 | 0.975 |
| 4/7/2021 | 0.785 |
| 4/9/2021 | 0.82 |
| 4/13/2021 | 0.225 |
| 4/13/2021 | 0.86 |
| 4/13/2021 | 0.86 |
| 4/13/2021 | 0 |
| 4/13/2021 | 0.21 |
| 4/13/2021 | 0.21 |
| 4/14/2021 | 0.34 |
| 4/18/2021 | 0.305 |
| 4/18/2021 | 0.53 |
| 4/19/2021 | 0.28 |
| 4/20/2021 | 0.303333 |
| 4/20/2021 | 0 |
| 4/22/2021 | 0.69 |
| 4/24/2021 | 0.72 |
| 4/24/2021 | 0.72 |
| 4/25/2021 | 0.303333 |
| 4/25/2021 | 0.303333 |
| 4/25/2021 | 0.225 |
| 4/25/2021 | 0.6 |
| 4/25/2021 | 0.66 |
| 4/25/2021 | 0.22 |
| 4/27/2021 | 0.78 |
| 4/27/2021 | 0.93 |
| 4/28/2021 | 0.02 |
| 4/28/2021 | 0.72 |
| 5/2/2021 | 0.076667 |
| 5/2/2021 | 0.595 |
| 5/3/2021 | 0.67 |
| 5/4/2021 | 0.8 |
| 5/4/2021 | 0.785 |
| 5/4/2021 | 0.785 |
| 5/5/2021 | 0.455 |
| 5/5/2021 | 0.73 |
| 5/6/2021 | 0.455 |
| 5/6/2021 | 0.59 |
| 5/6/2021 | 0.9 |
| 5/6/2021 | 0.68 |
| 5/7/2021 | 0.66 |
| 5/7/2021 | 0.095 |
| 5/7/2021 | 0.24 |
| 5/8/2021 | 0.015 |
| 5/8/2021 | 0.66 |
| 5/8/2021 | 0.24 |
| 5/9/2021 | 0.42 |
| 5/10/2021 | 0.325 |
| 5/10/2021 | 0.015 |
| 5/10/2021 | 0.513333 |
| 5/13/2021 | 0.56 |
| 5/13/2021 | 0.396667 |
| 5/13/2021 | 0.525 |
| 5/13/2021 | 0.455 |
| 5/15/2021 | 0.496667 |
| 5/16/2021 | 0.045 |
| 5/16/2021 | 0.235 |
| 5/16/2021 | 0.715 |
| 5/16/2021 | 0.825 |
| 5/17/2021 | 0.303333 |
| 5/17/2021 | 0.8 |
| 5/18/2021 | 0.55 |
| 5/18/2021 | 0.736667 |
| 5/18/2021 | 0.335 |
| 5/18/2021 | 0.24 |
| 5/19/2021 | 0.303333 |
| 5/19/2021 | 0.595 |
| 5/21/2021 | 0.175 |
| 5/21/2021 | 0.095 |
| 5/22/2021 | 0.565 |
| 5/22/2021 | 0.14 |
| 5/22/2021 | 0.67 |
| 5/23/2021 | 0.115 |
| 5/24/2021 | 0.5325 |
| 5/24/2021 | 0.17 |
| 5/24/2021 | 0.225 |
| 5/24/2021 | 0.045 |
| 5/24/2021 | 0.78 |
| 5/24/2021 | 0.78 |
| 5/24/2021 | 0.78 |
| 5/25/2021 | 0.615 |
| 5/25/2021 | 0.22 |
| 5/26/2021 | 0.01 |
| 5/26/2021 | 0.01 |
| 5/26/2021 | 0.135 |
| 5/26/2021 | 0.7 |
| 5/26/2021 | 0.43 |
| 5/27/2021 | 0.05 |
| 5/27/2021 | 0.09 |
| 5/29/2021 | 0 |
| 5/29/2021 | 0.6 |
| 5/31/2021 | 0.515 |
| 6/1/2021 | 0.645 |
| 6/1/2021 | 0.06 |
| 6/1/2021 | 0.323333 |
| 6/1/2021 | 0 |
| 6/1/2021 | 0.855 |
| 6/2/2021 | 0.28 |
| 6/2/2021 | 0.28 |
| 6/2/2021 | 0 |
| 6/2/2021 | 0.745 |
| 6/2/2021 | 0.655 |
| 6/2/2021 | 0.2 |
| 6/2/2021 | 0.71 |
| 6/3/2021 | 0.24 |
| 6/3/2021 | 0.495 |
| 6/4/2021 | 0.63 |
| 6/4/2021 | 0.225 |
| 6/4/2021 | 0.73 |
| 6/4/2021 | 0.27 |
| 6/4/2021 | 0.55 |
| 6/5/2021 | 0.24 |
| 6/5/2021 | 0.345 |
| 6/5/2021 | 0.36 |
| 6/6/2021 | 0.895 |
| 6/6/2021 | 0.056667 |
| 6/6/2021 | 0.063333 |
| 6/6/2021 | 0.035 |
| 6/7/2021 | 0.175 |
| 6/7/2021 | 0.175 |
| 6/7/2021 | 0.455 |
| 6/8/2021 | 0.505 |
| 6/8/2021 | 0.66 |
| 6/9/2021 | 0.305 |
| 6/9/2021 | 0.303333 |
| 6/9/2021 | 0.25 |
| 6/9/2021 | 0.6 |
| 6/9/2021 | 0.72 |
| 6/9/2021 | 0.55 |
| 6/10/2021 | 0.225 |
| 6/10/2021 | 0.455 |
| 6/10/2021 | 0.07 |
| 6/10/2021 | 0.095 |
| 6/10/2021 | 0.18 |
| 6/10/2021 | 0.825 |
| 6/11/2021 | 0.225 |
| 6/11/2021 | 0.255 |
| 6/11/2021 | 0.445 |
| 6/11/2021 | 0.035 |
| 6/11/2021 | 0.77 |
| 6/11/2021 | 0.056667 |
| 6/11/2021 | 0.48 |
| 6/12/2021 | 0 |
| 6/14/2021 | 0.755 |
| 6/14/2021 | 0.77 |
| 6/15/2021 | 0.13 |
| 6/15/2021 | 0.219485 |
| 6/15/2021 | 0.51 |
| 6/15/2021 | 0.285 |
| 6/16/2021 | 0.225 |
| 6/16/2021 | 0.225 |
| 6/16/2021 | 0.47 |
| 6/17/2021 | 0.95 |
| 6/17/2021 | 0.845 |
| 6/18/2021 | 0.1 |
| 6/18/2021 | 0.72 |
| 6/18/2021 | 0.83 |
| 6/19/2021 | 0.445 |
| 6/19/2021 | 0.7625 |
| 6/20/2021 | 0.433333 |
| 6/20/2021 | 0.06 |
| 6/20/2021 | 0.715 |
| 6/20/2021 | 0.546667 |
| 6/20/2021 | 0.546667 |
| 6/20/2021 | 0.546667 |
| 6/21/2021 | 0.81 |
| 6/21/2021 | 0.26 |
| 6/21/2021 | 0.26 |
| 6/21/2021 | 0.303333 |
| 6/21/2021 | 0.13 |
| 6/21/2021 | 0.476667 |
| 6/21/2021 | 0.36 |
| 6/23/2021 | 0.275 |
| 6/23/2021 | 0.24 |
| 6/24/2021 | 0.86 |
| 6/24/2021 | 0.02 |
| 6/24/2021 | 0.43 |
| 6/25/2021 | 0.89 |
| 6/25/2021 | 0.845 |
| 6/26/2021 | 0.67 |
| 6/27/2021 | 0.725 |
| 6/27/2021 | 0.73 |
| 6/27/2021 | 0.715 |
| 6/28/2021 | 0.003333 |
| 6/29/2021 | 0.085 |
| 6/29/2021 | 0.7 |
| 6/30/2021 | 0.25 |
| 6/30/2021 | 0 |
| 7/1/2021 | 0.885 |
| 7/1/2021 | 0.72 |
| 7/2/2021 | 0.46 |
| 7/2/2021 | 0.635 |
| 7/2/2021 | 0.845 |
| 7/4/2021 | 0.53 |
| 7/4/2021 | 0.195 |
| 7/4/2021 | 0.045 |
| 7/5/2021 | 0.075 |
| 7/5/2021 | 0.27 |
| 7/6/2021 | 0.235 |
| 7/6/2021 | 0.465 |
| 7/7/2021 | 0.67 |
| 7/7/2021 | 0.15 |
| 7/7/2021 | 0.15 |
| 7/8/2021 | 0.445 |
| 7/8/2021 | 0.57 |
| 7/12/2021 | 0.45 |
| 7/13/2021 | 0.655 |
| 7/15/2021 | 0.47 |
| 7/15/2021 | 0.9 |
| 7/15/2021 | 0.9 |
| 7/16/2021 | 0.09 |
| 7/18/2021 | 0.055 |
| 7/18/2021 | 0.1 |
| 7/18/2021 | 0.63 |
| 7/21/2021 | 0.82 |
| 7/22/2021 | 0.1 |
| 7/23/2021 | 0.455 |
| 7/24/2021 | 0.71 |
| 7/24/2021 | 0.465 |
| 7/24/2021 | 0.3 |
| 7/26/2021 | 0.896667 |
| 7/27/2021 | 0.7 |
| 7/30/2021 | 0.25 |
| 7/30/2021 | 0.21 |
| 7/30/2021 | 0.21 |
| 7/30/2021 | 0.21 |
| 8/1/2021 | 0.4675 |
| 8/2/2021 | 0.176667 |
| 8/3/2021 | 0.2 |
| 8/6/2021 | 0.63 |
| 8/6/2021 | 0.18 |
| 8/6/2021 | 0.71 |
| 8/7/2021 | 0 |
| 8/7/2021 | 0.675 |
| 8/7/2021 | 0 |
| 8/8/2021 | 0.003333 |
| 8/8/2021 | 0.06 |
| 8/9/2021 | 0.23 |
| 8/9/2021 | 0.546667 |
| 8/9/2021 | 0.885 |
| 8/10/2021 | 0.475 |
| 8/16/2021 | 0.14 |
| 8/17/2021 | 0.795 |
| 8/18/2021 | 0.45 |
| 8/21/2021 | 0.465 |
| 8/24/2021 | 0.345 |
| 8/25/2021 | 0.01 |
| 8/27/2021 | 0.483333 |
| 8/27/2021 | 0.36 |
| 8/28/2021 | 0.2 |
| 8/30/2021 | 0.235 |
| 9/4/2021 | 0.265 |
| 9/4/2021 | 0.265 |
| 9/4/2021 | 0.635 |
| 9/6/2021 | 0.08 |
| 9/8/2021 | 0.6 |
| 9/11/2021 | 0.34 |
| 9/11/2021 | 0.06 |
| 9/11/2021 | 0.06 |
| 9/12/2021 | 0.835 |
| 9/15/2021 | 0.21 |
| 9/17/2021 | 0.21 |
| 9/21/2021 | 0.165 |
| 9/21/2021 | 0.165 |
| 9/21/2021 | 0.79 |
| 9/22/2021 | 0.255 |
| 9/22/2021 | 0.33 |
| 9/25/2021 | 0.115 |
| 9/28/2021 | 0.583333 |
| 9/28/2021 | 0.38 |
| 9/30/2021 | 0.39 |
| 10/1/2021 | 0.67 |
| 10/3/2021 | 0.065 |
| 10/3/2021 | 0.113333 |
| 10/4/2021 | 0.035 |
| 10/4/2021 | 0.785 |
| 10/10/2021 | 0.515 |
| 10/12/2021 | 0.635 |
| 10/13/2021 | 0.305 |
| 10/14/2021 | 0.015 |
| 10/14/2021 | 0.08 |
| 10/15/2021 | 0.065 |
| 10/15/2021 | 0.39 |
| 10/15/2021 | 0.395 |
| 10/16/2021 | 0.35 |
| 10/17/2021 | 0.52 |
| 10/18/2021 | 0.28 |
| 10/18/2021 | 0.4 |
| 10/18/2021 | 0.345 |
| 10/20/2021 | 0.76 |
| 10/20/2021 | 0.56 |
| 10/21/2021 | 0.395 |
| 10/22/2021 | 0.18 |
| 10/23/2021 | 0.516667 |
| 10/24/2021 | 0.03 |
| 10/27/2021 | 0.3 |
| 10/27/2021 | 0.06 |
| 10/28/2021 | 0.755 |
| 10/29/2021 | 0.843333 |
| 11/1/2021 | 0.195 |
| 11/2/2021 | 0.515 |
| 11/3/2021 | 0.305 |
| 11/4/2021 | 0.16 |
| 11/6/2021 | 0.43 |
| 11/9/2021 | 0.53 |
| 11/9/2021 | 0.94 |
| 11/14/2021 | 0.16 |
| 11/15/2021 | 0.305 |
| 11/16/2021 | 0.42 |
| 11/16/2021 | 0.165 |
| 11/17/2021 | 0.45 |
| 11/18/2021 | 0.903333 |
| 11/18/2021 | 0.903333 |
| 11/20/2021 | 0.485 |
| 11/21/2021 | 0.705 |
| 11/26/2021 | 0.335 |
| 11/26/2021 | 0.335 |
| 11/26/2021 | 0.46 |
| 11/26/2021 | 0.46 |
| 11/26/2021 | 0.46 |
| 11/26/2021 | 0.46 |
| 11/29/2021 | 0.7 |
| 11/29/2021 | 0.7 |
| 11/30/2021 | 0.305 |
| 11/30/2021 | 0.305 |
| 12/8/2021 | 0.095 |
| 12/8/2021 | 0.095 |
| 12/8/2021 | 0.93 |
| 12/8/2021 | 0.93 |
| 12/27/2021 | 0.4375 |
| 12/27/2021 | 0.4375 |
| 1/5/2022 | 0.93 |
| 1/5/2022 | 0.93 |
| 1/24/2022 | 0.15 |
| 1/24/2022 | 0.15 |
| 2/1/2022 | 0.36 |
| 2/1/2022 | 0.36 |
| 2/6/2022 | 0.175 |
| 2/6/2022 | 0.175 |
| 2/9/2022 | 0.2 |
| 2/9/2022 | 0.2 |
| 2/13/2022 | 0.24 |
| 2/15/2022 | 0.09 |
| 2/22/2022 | 0.23 |
| 3/5/2022 | 0.595 |
| 3/7/2022 | 0.165 |
| 3/14/2022 | 0.845 |
| 3/15/2022 | 0.5 |
| 3/17/2022 | 0.483333 |
| 3/19/2022 | 0.81 |
| 3/24/2022 | 0.38 |
| 3/31/2022 | 0.24 |
| 4/2/2022 | 0.195 |
| 4/2/2022 | 0.4575 |
| 4/5/2022 | 0.653333 |
| 4/8/2022 | 0.74 |
| 4/13/2022 | 0.06 |
| 4/14/2022 | 0.025 |
| 4/19/2022 | 0.555 |
| 4/21/2022 | 0.675 |
| 4/21/2022 | 0.336667 |
| 4/21/2022 | 0.56 |
| 4/22/2022 | 0 |
| 4/26/2022 | 0.175294 |
| 4/27/2022 | 0.95 |
| 4/28/2022 | 0.99 |
| 4/28/2022 | 0.125 |
| 4/29/2022 | 0.453333 |
| 5/1/2022 | 0.55 |
| 5/1/2022 | 0.88 |
| 5/2/2022 | 0.14 |
| 5/3/2022 | 0.05 |
| 5/3/2022 | 0.14 |
| 5/4/2022 | 0.046667 |
| 5/4/2022 | 0.253333 |
| 5/5/2022 | 0.2025 |
| 5/6/2022 | 0.125 |
| 5/6/2022 | 0 |
| 5/7/2022 | 0.62 |
| 5/7/2022 | 0.49 |
| 5/9/2022 | 0.06 |
| 5/9/2022 | 0.24 |
| 5/10/2022 | 0.98 |
| 5/11/2022 | 0.86 |
| 5/12/2022 | 0.01 |
| 5/13/2022 | 0.78 |
| 5/14/2022 | 0.78 |
| 5/14/2022 | 0.765 |
| 5/14/2022 | 0.295 |
| 5/17/2022 | 0.435 |
| 5/17/2022 | 0.003333 |
| 5/18/2022 | 0.425 |
| 5/19/2022 | 0.156667 |
| 5/20/2022 | 0.543333 |
| 5/20/2022 | 0 |
| 5/20/2022 | 0.06 |
| 5/22/2022 | 0.795 |
| 5/23/2022 | 0.895 |
| 5/24/2022 | 0.125 |
| 5/24/2022 | 0.83 |
| 5/25/2022 | 0.305 |
| 5/26/2022 | 0.335 |
| 5/26/2022 | 0.545 |
| 5/27/2022 | 0.955 |
| 5/30/2022 | 0.21 |
| 5/31/2022 | 0.3 |
| 6/1/2022 | 0.91 |
| 6/2/2022 | 0.795 |
| 6/3/2022 | 0.05 |
| 6/6/2022 | 0 |
| 6/6/2022 | 0.67 |
| 6/7/2022 | 0.38 |
| 6/8/2022 | 0.385 |
| 6/8/2022 | 0.65 |
| 6/10/2022 | 0.47 |
| 6/14/2022 | 0.845 |
| 6/15/2022 | 0.056667 |
| 6/16/2022 | 0.465 |
| 6/16/2022 | 0.285 |
| 6/17/2022 | 0.05 |
| 6/17/2022 | 0.65 |
| 6/17/2022 | 0.285 |
| 6/17/2022 | 0.01 |
| 6/17/2022 | 0.855 |
| 6/19/2022 | 0.305 |
| 6/19/2022 | 0.87 |
| 6/19/2022 | 0.045 |
| 6/19/2022 | 0 |
| 6/20/2022 | 0.355 |
| 6/20/2022 | 0.77 |
| 6/20/2022 | 0.685 |
| 6/21/2022 | 0.71 |
| 6/21/2022 | 0.77 |
| 6/24/2022 | 0.65 |
| 6/24/2022 | 0.305 |
| 6/26/2022 | 0 |
| 6/26/2022 | 0.35 |
| 6/30/2022 | 0.053333 |
| 7/2/2022 | 0.05 |
| 7/4/2022 | 0.765 |
| 7/6/2022 | 0.675 |
| 7/6/2022 | 0.34 |
| 7/7/2022 | 0.765 |
| 7/7/2022 | 0.8175 |
| 7/8/2022 | 0.8175 |
| 7/9/2022 | 0.345 |
| 7/9/2022 | 0.836667 |
| 7/9/2022 | 0.175 |
| 7/10/2022 | 0 |
| 7/10/2022 | 0.8175 |
| 7/11/2022 | 0.34 |
| 7/12/2022 | 0.235 |
| 7/13/2022 | 0.15 |
| 7/15/2022 | 0.815 |
| 7/17/2022 | 0.8175 |
| 7/19/2022 | 0.64 |
| 7/20/2022 | 0.01 |
| 7/23/2022 | 0.8175 |
| 7/24/2022 | 0.89 |
| 7/26/2022 | 0.765 |
| 7/28/2022 | 0.133333 |
| 7/29/2022 | 0.04 |
| 7/31/2022 | 0.056667 |
| 7/31/2022 | 0.69 |
| 8/3/2022 | 0 |
| 8/6/2022 | 0.0825 |
| 8/10/2022 | 0.805 |
| 8/12/2022 | 0.07 |
| 8/19/2022 | 0.326667 |
| 8/22/2022 | 0.65 |
| 8/24/2022 | 0.625 |
| 8/25/2022 | 0.07 |
| 8/27/2022 | 0.396667 |
| 8/28/2022 | 0.65 |
| 8/30/2022 | 0.49 |
| 8/30/2022 | 0.645 |
| 8/31/2022 | 0.63 |
| 8/31/2022 | 0 |
| 9/1/2022 | 0.265 |
| 9/3/2022 | 0.67 |
| 9/3/2022 | 0.636667 |
| 9/3/2022 | 0.52 |
| 9/4/2022 | 0.52 |
| 9/4/2022 | 0 |
| 9/5/2022 | 0 |
| 9/6/2022 | 0 |
| 9/6/2022 | 0.065 |
| 9/7/2022 | 0.09 |
| 9/8/2022 | 0.74 |
| 9/8/2022 | 0.04 |
| 9/9/2022 | 0.325 |
| 9/9/2022 | 0.235 |
| 9/9/2022 | 0.665 |
| 9/10/2022 | 0.08 |
| 9/11/2022 | 0.078258 |
| 9/13/2022 | 0.905 |
| 9/15/2022 | 0.06 |
| 9/18/2022 | 0.77 |
| 9/22/2022 | 0.59 |
| 9/29/2022 | 0.045 |
| 10/4/2022 | 0.015 |
